# A Robust Framework for Maize Elite Line Genome Editing Through Enhanced HI‐Edit via LbCas12a Activity Optimization

**DOI:** 10.1111/pbi.70715

**Published:** 2026-07-04

**Authors:** Dawei Liang, Huanhuan Guo, Juan Wei, Fugui Zhu, Yuguo Zhang, Julie Green, Yun Ji, Huaibing Jin, Xiujuan Zhang, Huaping Gui, Hongmei Dan, Yubo Liu, Yu Zhang, Han Wang, Yutong Jiang, Lizhao Geng, Jian Lv, Wen Cai, Weibin Song, Timothy Kelliher, Xi Chen, Rachel Egger

**Affiliations:** ^1^ State Key Laboratory of Maize Bio‐breeding, National Maize Improvement Center, Department of Plant Genetics and Breeding China Agricultural University Beijing China; ^2^ State Key Laboratory of Crop Germplasm Innovation and Molecular Breeding Syngenta Biotechnology (China) Co., Ltd Beijing China; ^3^ Syngenta Seeds Research & Development Research Triangle Park North Carolina USA; ^4^ State Key Laboratory of Crop Gene Resources and Breeding, National Key Facility for Crop Gene Resources and Genetic Improvement, Institute of Crop Sciences Chinese Academy of Agricultural Sciences Beijing China; ^5^ Genome Editing Center North Carolina State University Raleigh North Carolina USA

**Keywords:** genome editing, haploid editing rate (HER), haploid induction (HI), UBA2 fusion, zygote editing rate (ZER)

## Abstract

Haploid induction coupled with genome editing (HI‐Edit) enables direct modification of commercial crop varieties, bypassing the need for trait introgression or direct transformation of elite lines with CRISPR machinery. However, its widespread application has been constrained by low haploid editing rates (HER), the proportion of haploids carrying edits within the short window between double fertilization and uniparental chromosome elimination. Here, we report substantial improvements in maize HI‐Edit efficiency through three complementary strategies: (1) driving an optimized LbCas12a variant (LbCas12aV) using promoters that are highly active in sperm cells and early zygotes; (2) applying a post‐pollination heat treatment; and (3) fusing LbCas12aV with the UBA2 domain (ubiquitin‐associated domain‐2 of *Arabidopsis thaliana* RAD23) to enhance protein stability during haploid induction. Post‐pollination heat treatment alone increased HER to 19.1% (up to 12‐fold improvement depending on the target site), providing a simple and effective method to boost the yield of edited doubled haploid (DH) plants. UBA2 fusion improved HER by 6‐fold at the *Waxy1* (*Wx1*) locus and 4.5‐fold at the *Glossy2* (*Gl2*) locus under normal conditions. Strikingly, combining UBA2 fusion with heat treatment raised the average HER to 25% across multiple events targeting *Wx1*, with the highest HER reaching 33%. Collectively, these findings demonstrate that increasing CRISPR‐Cas protein abundance and modulating environmental conditions can overcome key bottlenecks in HI‐Edit. We establish a robust, scalable framework that is readily transferable to other crops for elite‐line genome editing.

## 1 Introduction

**FIGURE 1:**
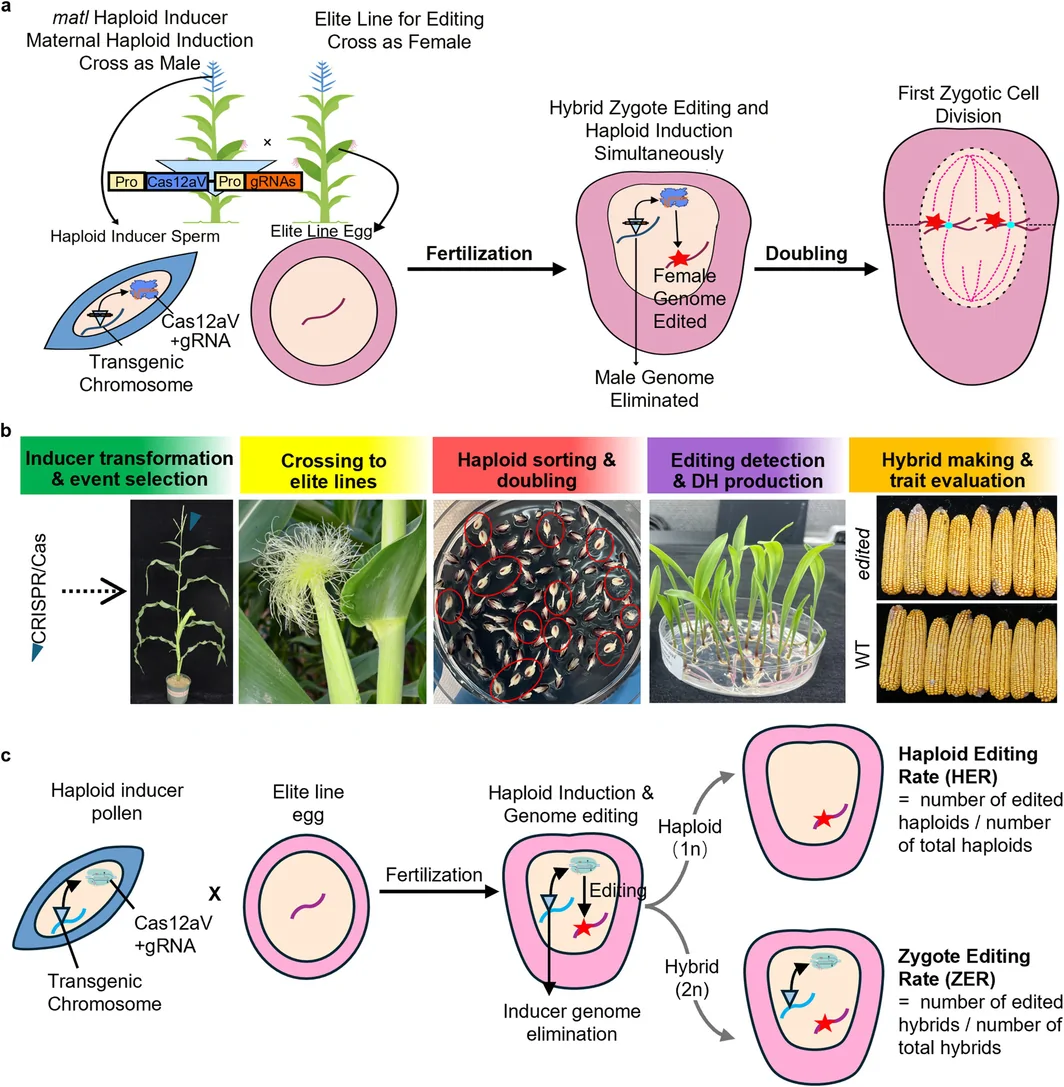
Establishment of CRISPR‐Cas12aV mediated HI‐Edit technology. (a) Technical principle of HI‐Edit. The transformable haploid inducer carrying the editing machinery is used as a male to pollinate the target elite line. Haploid induction and editing of the elite line genome simultaneously occur after fertilization, and then the edited double haploid elite line is generated after chromosome doubling. The blue and pink/red indicate the chromosome or cytoplasm from haploid inducer and target elite line, respectively. The triangle indicates the editing machinery, and the red star indicates the edited gene. (b) HI‐Edit implementation process. The inducer is transformed with Cas12aV vectors and T0 1‐copy events are selected. T1 Cas‐homozygous plants are used as male to cross with elite lines. Putative haploids are sorted by embryo colour and double haploid (DH) plants are recovered through embryo rescue followed by chromosome doubling. Edited DHs are identified by molecular characterization (transgene presence, genotype of target gene). Then the trait evaluation of edited DHs of elite lines is conducted. The blue triangle indicates the editing machinery; the red circles highlight the putative haploids. (c) Detection of haploid editing rate (HER) and zygote editing rate (ZER). Embryos isolated from F1 ears were used and haploids or diploids (hybrids) were identified by colour sorting and molecular confirmation. HER and ZER were calculated by dividing the total haploid or hybrid number by edited haploid or zygotic edited hybrid number, respectively.

Genome editing holds great promise for introducing valuable trait variants into crop plants (Blankenagel et al. 2022; Li et al. 2024; Liu et al. 2021). However, a major bottleneck remains how to efficiently deploy these genetic improvements across the diverse elite germplasm which are mostly recalcitrant to genetic transformation (Li, Geng, et al. 2025). Haploid induction coupled with genome editing, termed HI‐Edit, offers a compelling solution (Kelliher et al. 2019). Unlike traditional marker‐assisted introgression or direct transformation of elite varieties, HI‐Edit uses a single cross with a haploid inducer line carrying the CRISPR machinery to simultaneously induce haploids and introduce targeted edits (Figure 1a). This strategy bypasses the need for elite line transformation and avoids the lengthy backcrossing required to remove unlinked transgenes (Figure S1).

In typical transformation‐based editing, editing occurs in embryogenic callus or regenerating shoots. By contrast, HI‐Edit operates in the zygote shortly after sperm‐egg fusion, before the haploid inducer genome is eliminated. Chromosome loss is thought to happen very early, usually within the first one or two cell divisions, in both *MATRILINEAL* and *CENTROMERIC HISTONE3* based induction systems (Kelliher et al. 2017; Liu et al. 2017; Marimuthu et al. 2021). Due to this narrow editing window, potentially low expression of the T‐DNA‐encoded CRISPR components, and possible differences in chromatin accessibility at target sites, reported haploid editing rates (HER, the proportion of haploids carrying the desired edit) have averaged between 1% and 7%, although using the most efficient editing tools (Kelliher et al. 2019; Li, Fu, et al. 2025; Tian et al. 2024; Wang et al. 2019). This low efficiency has become the primary constraint limiting the widespread application of HI‐Edit in both academic research and commercial breeding.

In this study, we hypothesized that three major factors could enhance HER: (1) the expression level of CRISPR‐Cas in sperm cells and early zygotes, (2) the stability of the Cas protein, and (3) the environmental conditions during fertilization and early embryogenesis. We therefore set out to optimize these variables using a streamlined HI‐Edit pipeline (Figure 1b) and a newly developed elite transformable haploid inducer line, SYN‐INBD56 (Brent et al. 2024), combined with an efficient LbCas12a variant (LbCas12aV) that harbours a long linker and two amino‐acid substitutions (D156R and C965S) (Xu et al. 2024). We used haploid editing rate (HER) and zygote editing rate (ZER) as key performance indicators (Figure 1c). To improve HI‐Edit efficiency, we pursued three complementary approaches. First, we mined maize transcriptomic data to identify novel promoters with strong activity in sperm cells and early zygotes and used them to drive LbCas12aV expression. Second, we made the Cas protein more stable by fusing it with the UBA2 domain (ubiquitin‐associated‐2 domain of *Arabidopsis thaliana* RAD23) of protein, which is known to protect against ubiquitin‐proteasome degradation. Third, we applied a short, moderate heat treatment to the ear after pollination, based on the rationale that elevated temperature could enhance Cas12a enzymatic activity. When combined, the UBA2 fusion and the optimized heat treatment elevated HER to as high as 33%, representing a simple, scalable, and robust technological advance that can be readily transferred to other crop species.

## 2 Results

### 2.1 Male‐Gamete/Zygote Promoter Discovery and Haploid Editing Validation

**FIGURE 2:**
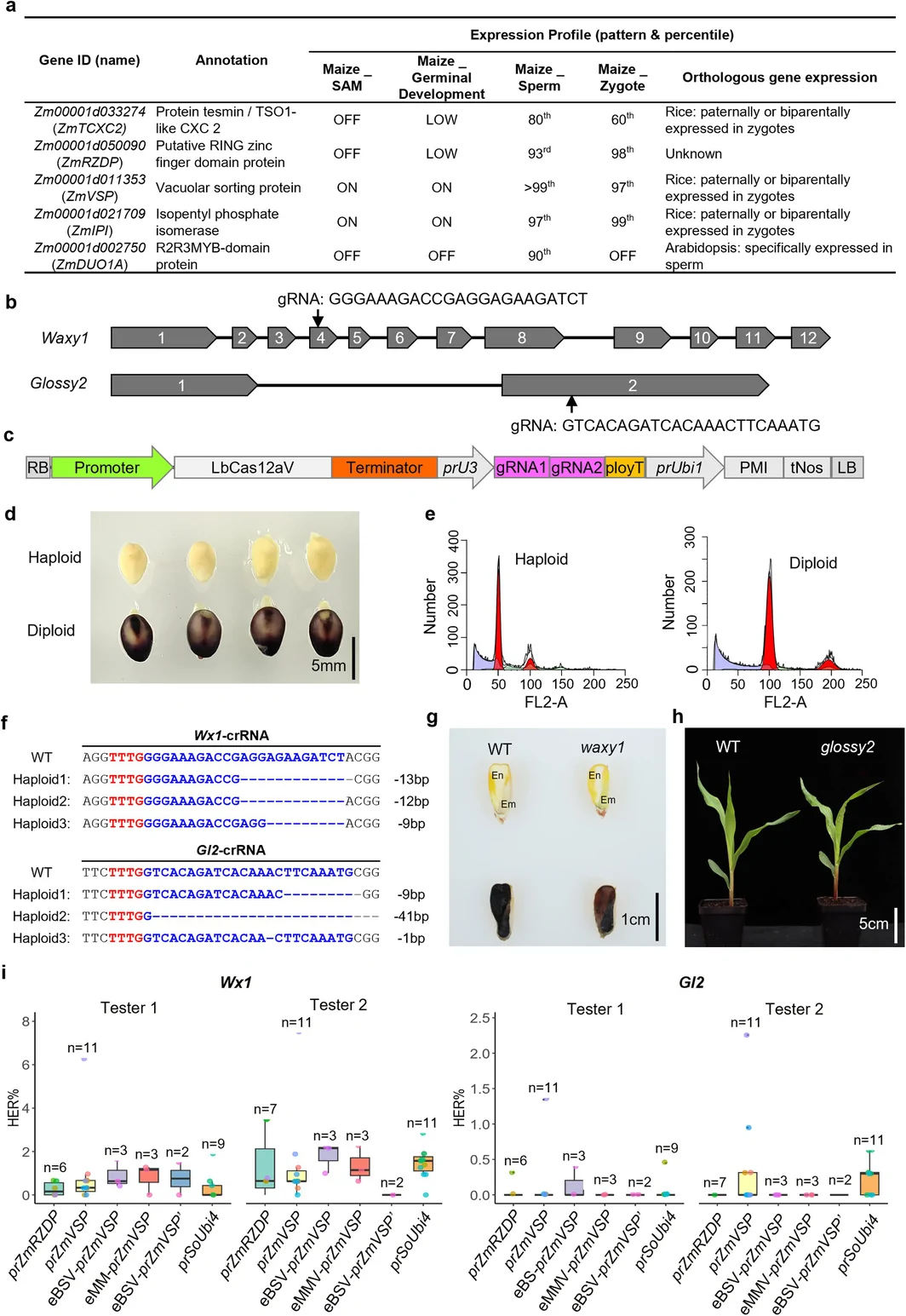
LbCas12aV mediated HI‐Edit by using male‐gamete/zygote promoters. (a) Candidate promoters for HI‐Edit. (b) Structure of gene targets and gRNA target sites. (c) Schematic T‐DNA vector for HI‐Edit test. LbCas12aV was driven by the selected promoters (*prZmrRZDP*, *prZmVSP*, *prSoUbi4*) combined with corresponding terminators. tNOS, NOS terminator; *prU3*, rice U3 Pol III promoter; gRNA1, guide RNA targeting *Glossy2* (*Gl2*); gRNA2, guide RNA targeting *Waxy1* (*Wx1*); *prUbi1*, maize ubiquitin1 promoter; RB/LB, T‐DNA right/left border; PMI (phosphomannose isomerase) was used as a plant selectable marker. (d) Identification of haploids and diploids (hybrids) by embryo colour sorting of F1 ears. (e) Ploidy confirmation using flow cytometry. (f) Genotypes of target gene *Wx1* and *Gl2* in edited haploids. The target sequence and PAM are highlighted in blue and red, respectively. (g) Phenotype of *Wx1* mutation in recovered double haploids of tester 1. Endosperm of *wx1* mutants containing almost exclusively amylopectin showed a red/brown phenotype after iodine staining; while in wild‐type endosperm, the presence of amylose forms a blue‐black complex with iodine; En, endosperm; Em, embryo. (h) Phenotype of *Gl2* mutants recovered from edited double haploids of tester 1. *Gl2* mutant seedlings showed glossy leaf phenotype (water droplets as discrete beads on the leaf surface) compared to wild‐type waxy leaf surface (water droplets spreading or sliding off). (i) The haploid editing rate (HER) on gene targets *Wx1* (left) and *Gl2* (right) were evaluated by event and tester. Events used for outcross were generated from vectors which LbCas12aV were driven by promoters of *ZmRZDP* (*prZmRZDP*), *ZmVSP* (*prZmVSP*), combining *prZmVSP* with viral enhancers (eBSV‐pr*ZmVSP*, eMMV‐*prZmVSP*), and TATA box mutations of *ZmVSP* (*prZmVSP’*), *SoUbi4* (*prSoUbi4*). Different colour dots indicate different events. Sample sizes (n) are indicated above boxes. Boxplots show medians, quartiles and whiskers (1.5× IQR).

To test whether increasing Cas expression during fertilization improves haploid editing efficiency (HER), we first identified promoters with strong activity in maize sperm cells and early zygotes. By analysing maize RNA‐Seq data (Chen et al. 2017; Dukowic‐Schulze et al. 2014; Hoopes et al. 2019) and rice zygotic transcription data (Anderson et al. 2017), we selected five candidate promoters: *ZmTCXC2* (tesmin/TSO1‐like CXC2), *ZmRZDP* (putative RING zinc finger protein), *ZmVSP* (vacuolar sorting protein), *ZmIPI* (isopentyl phosphate isomerase) and *ZmDUO1‐A* (ortholog of Arabidopsis DUO1, sperm‐specifically expressed) (Figure 2a). The constitutive promoters *prOsActin1* and *prSoUbi4* were also tested (Figure S2a). These promoters were used to drive expression of an optimized LbCas12a variant (LbCas12aV) (Xu et al. 2024) in a transformable haploid inducer line SYN‐INBD56 (Brent et al. 2024).

Quantitative RT‐PCR on T1 homozygous plants revealed that all tested promoters except *ZmDUO1‐A* drove detectable LbCas12aV expression in maize pollen. The highest pollen expression levels were observed for *prZmVSP*, *prZmRZDP* and *prSoUbi4* (relative expression: 5817, 2366 and 2016, respectively; Figure S2b). These three promoters were therefore selected for HI‐Edit validation. To further enhance expression and test HER, another batch of binary vectors targeting *Waxy1* and *Glossy2* were constructed, containing viral enhancers (eBSV or eMMV) and a modified TATA box upstream of *prZmVSP* (Figure 2b,c and Figure S3).

Binary vectors were transformed into SYN‐INBD56 and yielded an average transformation frequency of 5.76% (Table S1), suggesting the advantage of this inducer for more efficient introduction of editing machinery compared to previous inducers and methods (Kelliher et al. 2019; Li, Fu, et al. 2025; Tian et al. 2024; Wang et al. 2019). A total of 35 T0 positive events were selected (Table S2), and T1 LbCas12aV‐homozygous plants were crossed as males with two tester lines (Tester 1: Stiff Stalk; Tester 2: non‐Stiff Stalk). From 1207 F1 ears, we isolated 189 966 embryos (Table 1). Putative haploids were identified by embryo colour sorting (Figure 2d) and confirmed by flow cytometry (Figure 2e) and molecular analysis (Table S3). Overall haploid induction rates (HIR) ranged from 9.6% to 14.55%, with an average of 11.56% (10 908 haploids out of 94 324 F1 embryos) for Tester 1 and 12.71% (12 160 haploids identified from 95 642 F1 embryos) for Tester 2 (Table 1 and Table S4), consistent with previously reported HIR values for this inducer.

**TABLE 1:** Outcross and haploid generation with LbCas12aV‐homo inducer (SYN‐ INBD56).

Vector id	LbCas12aV promoter	Haploid inducer transformed	Event no.	Tester	F1 ear no.	F1 embryo no.	Haploid no.	Haploid induction rate (HIR) %
27 145	*prZmRZDP*	SYN‐ INBD56	6	Tester 1, 2	238	34 712	4816	13.87%
27 146	*prZmVSP*	SYN‐ INBD56	11	Tester 1, 2	424	70 421	8133	11.55%
27 680	*prSoUbi4*	SYN‐ INBD56	9	Tester 1, 2	310	48 956	5801	11.85%
28 255	eBSV‐*prZmVSP*	SYN‐ INBD56	3	Tester 1, 2	83	12 505	1564	12.51%
28 291	eMMV‐*prZmVSP*	SYN‐ INBD56	3	Tester 1, 2	89	13 431	1507	11.22%
28 292	eBSV‐*prZmVSP’*	SYN‐ INBD56	2	Tester 1, 2	63	9941	1247	12.54%
**Total/Mean**					1207	189 966	23 068	12.14%

Genome edited haploids/diploids were detected by next generation sequencing (NGS), then the haploid editing rate (HER) and zygote editing rate (ZER) were calculated accordingly. All tested vectors produced mutations at both target sites in haploids (Figure 2f). The edited haploids were recovered and demonstrated typical phenotypes of *Wx1* and *Gl2* mutants (Figure 2g,h). Haploid editing rates for *Wx1* (HER^*Wx1*^) ranged from 0% to 7.5% across events (Figure 2i and Table S5). The highest HER^*Wx1*^ (7.5%) was obtained with vector 27 146 (*prZmVSP*) when crossed with Tester 2. The same event also gave the highest HER^*Gl2*^ (2.3%) in Tester 2. Vector 27 145 (*prZmRZDP*) produced up to 3.5% HER^*Wx1*^. Notably, vector 28 255 (eBSV‐*prZmVSP*) enabled simultaneous editing of both genes in a single haploid (Table S5). Adding viral enhancers or a stronger TATA box did not further increase HER (vector 28 292; Figure 2i). All detected mutations were deletions of 2–82 bp, with 63.6% being < 15 bp deletions (Tables S6 and S7). These HER values are comparable to or higher than previously reported Cas9‐based HI‐Edit efficiencies (Kelliher et al. 2019; Li, Fu, et al. 2025; Wang et al. 2019).

We also measured zygote editing rates (ZER) using F1 diploid embryos. The ZER^*Wx1*^ ranged from 0% to 75%, and ZER^*Gl2*^ from 0%–18.8% (Figure S4 and Table S5). The event with the highest HER (27146‐E11, Tester 2) also showed the highest ZER (75% for *Wx1* in Tester 1). Notably, across most F1 populations, ZER^*Wx1*^ was consistently higher than ZER^*Gl2*^, mirroring the trend observed for HER (Figure 2i and Figure S4).

### 2.2 Boosting Haploid Editing Rate (HER) by Heat Treatment After Fertilization

**FIGURE 3:**
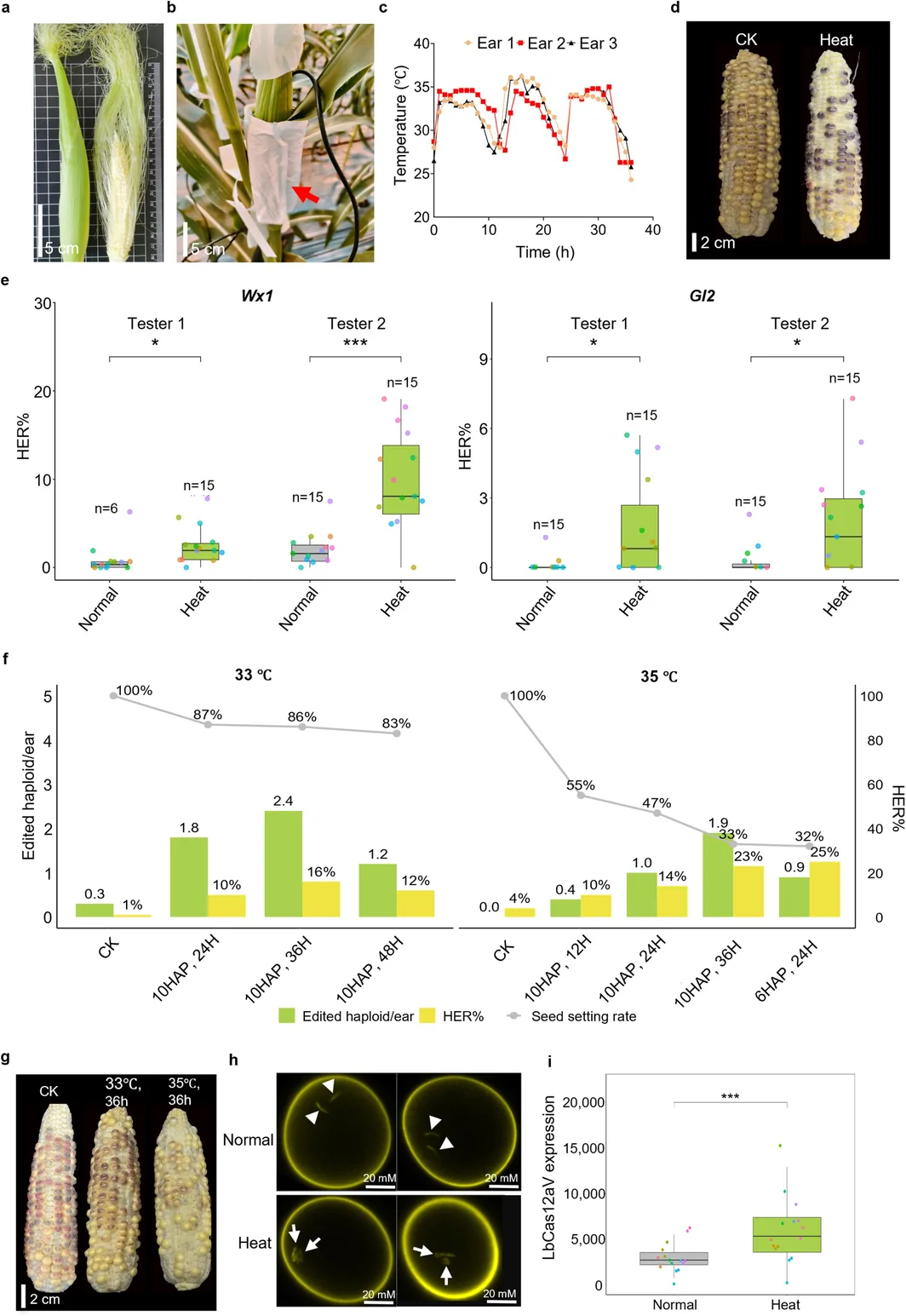
HI‐Edit improvement through heat treatment. (a) The optimal starting point for heat‐treatment was defined based on silk length and speed of pollen tube elongation. (b) Heat treatment during haploid induction. LbCas12aV homozygous inducer plants from selected vectors and events were used as males to cross with Tester 1 and Tester 2. After pollination, the ears were wrapped with a heat pack from 10 h after pollination (HAP); packs were replaced every 12 h. The red arrow indicates heat pack. (c) Ear temperature was measured by the sensor inserted in the husk; the dips in the temperatures represent the heating duration of the packs. (d) After heat treatment, ears were harvested for haploid or diploid identification and editing detection. (e) The haploid editing rate (HER) under normal and heat‐treatment conditions. Different colour dots indicate different events. Sample sizes (n) are indicated above boxes. Boxplots show medians, quartiles and whiskers (1.5× IQR). Asterisks denote significant differences (Welch's *t*‐test: *p < 0.05, **p < 0.01, ***p < 0.001). (f) Heat treatment optimization. LbCas12aV‐homozygous plants of 28 291‐E2 were used as male to cross with Tester 2. Heat treatment was conducted with hot water pack; heating at different hours after pollination (HAP), duration (12, 24, 36, 48 h) and temperature (33°C, 35°C) were tested. Haploid editing rate (HER, yellow bar), seed setting (grey line), and edited haploids per ear (green bar) were evaluated. (g) F1 ears after heat‐treatment. (h) Maize pollen grains after heat treatment. Fresh maize pollen was collected from wildtype greenhouse plants and incubated at 37°C for 1 h. The pollen was stained with 4′,6‐diamidino‐2‐phenylindole (DAPI) to label DNA and visualize the sperm nuclei (white arrowheads, control; arrows, heat treated). (i) LbCas12aV expression after heat treatment. LbCas12aV‐homozygous plants from 15 events of vectors 27 146, 27 680, 28 255, 28 291, 28 292 and 28 825 were grown to V3 stage, and then put into incubators (39°C, 72 h) and normal condition (day/night at 25°C/20°C); then the leaves were sampled for quantitative reverse‐transcriptional real‐time PCR (qRT‐PCR) analysis. 3 plants per event were sampled. Boxplots show medians, quartiles and whiskers (1.5× IQR); different colour dots indicate different events (mean expression, Welch's *t*‐test: *p < 0.05, **p < 0.01, ***p < 0.001).

LbCas12a is originally derived from *Lachnospiraceae bacteria* and exhibits optimal activity at 35°C–37°C, higher than typical plant growth temperatures (Malzahn et al. 2019; Schindele and Puchta 2020). High temperature also affects chromatin states, leading to histone mobilization and changing gene expression (Huang et al. 2023). We hypothesized that a transient post‐pollination heat treatment might enhance haploid editing by increasing Cas12a activity, promoting open chromatin, or up‐regulating transgene expression. To test this, we developed a simple ear heat‐treatment method. Firstly, we defined the starting point of heat treatment—10 h after pollination (HAP), which is the minimum time needed for the pollen tube to reach the embryo sack based on the silk length (Figure 3a) and previous research of fertilization mechanisms in maize (Zhou et al. 2017). Starting at 10 HAP, we wrapped F1 ears with heat packs that maintained the ear temperature at 35°C for 36 h (Figure 3b,c). Controls were grown under normal greenhouse conditions (25°C–30°C day/16°C–20°C night). HI‐Edit efficiency was evaluated in 19 events from six vectors under heat and control. LbCas12aV homozygous plants were crossed with tester 1, generating 4073 haploids (from 322 ears) and 1220 haploids (from 139 ears) under normal and heat‐treatment conditions, respectively (Table S8); while 4066 haploids (from 291 ears, normal condition) and 1552 haploids (from 155 ears, heat‐treated) were generated when crossed with tester 2 (Table S8).

As the result showed in Figure 3d and Table S8, heat treatment reduced seed set by 37% (Tester 1) and 24% (Tester 2). Nevertheless, HER^*Wx1*^ increased substantially. For Tester 1, the average HER^*Wx1*^ rose from 0.76% (control) to 2.13% under heat, with 10 out of 15 events exceeding 1% (versus only 2 events in control). For Tester 2, the average HER^*Wx1*^ increased from 1.52% to 9.15%, and the highest event reached 19.1% (Figure 3e and Table S8). Improvements were even more significant for the harder‐to‐edit *Gl2* target: heat treatment increased average HER^*Gl2*^ by 11‐fold (Tester 1) and 12‐fold (Tester 2), with maximum values of 5.71% and 7.27%, respectively (Figure 3e and Table S8). Simultaneous editing of both *Wx1* and *Gl2* in the same haploid was also observed more frequently under heat (32 haploids, HER^*Wx1* + *Gl2*^ up to 5.45%) compared to control (4 haploids, HER^*Wx1* + *Gl2*^ ≤ 0.9%).

Consistent with the increase in HER, heat treatment also elevated zygote editing rates (ZER) in both tester backgrounds and for both target genes (Figure S5). Under normal conditions, ZER^*Wx1*^ averaged 22.6% across events in Tester 1 and 18.4% in Tester 2; after heat treatment, these values increased to 35.9% and 31.2%, respectively. The effect was even more remarkable for the less efficient *Gl2* locus. Control ZER^*Gl2*^ ranged from 0% to 12.5% depending on the event and tester, whereas heat treatment raised ZER^*Gl2*^ up to 50% in several events, with average values increasing by 2.5‐ to 4‐fold (Figure S5 and Table S9). These results indicate that the improved haploid editing under heat is accompanied by a parallel enhancement of editing events in the zygote.

To balance HER gain against seed set reduction, we optimized heat‐treatment method. Two new methods for applying heat‐treatment were tested: hot water circulation system (hot water pack, Figures S6a‐6c) and hot room treatment (Figure S6d). The hot water pack method was simple to operate (no need to replace packs) and conferred the most accurate and stable temperature control (Figure S6b). With optimized heat‐treatment method (hot water pack), we further investigated heat‐treatment factors including start time for treatment, temperature, and duration. As shown in Figure 3f, the best balance was achieved with 33°C for 36 h starting at 10 HAP, yielding an average HER^*Wx1*^ of 16% and a seed set of 86% of control, resulting in 2.4 edited haploids per ear—the highest HI‐Edit output (Figure 3f,g). Higher temperature (35°C) or longer duration further increased HER (up to 23%) but caused > 67% seed set reduction.

To investigate the attributions of increased haploid editing rate (HER) under heat treatment, we imaged heat‐treated pollen grains on haploid inducer pollen using 4′,6‐diamidino‐2‐phenylindole (DAPI) staining and found that the sperm nuclei appeared larger under heat (37°C for 1 h) compared to control (Figure 3h), consistent with heat‐induced chromatin relaxation (Huang et al. 2023). Moreover, qRT‐PCR showed that heat treatment increased LbCas12aV expression levels by approximately one‐fold (Figure 3i). We identified several heat shock elements (HSEs) in the *prZmVSP* and *prZmRZDP* promoters (e.g., AAATAAAAAATAAAA in *prZmVSP*), which may contribute to heat‐induced transgene expression.

### 2.3 UBA2 Fusion Increased Haploid Editing Rate (HER)

**FIGURE 4:**
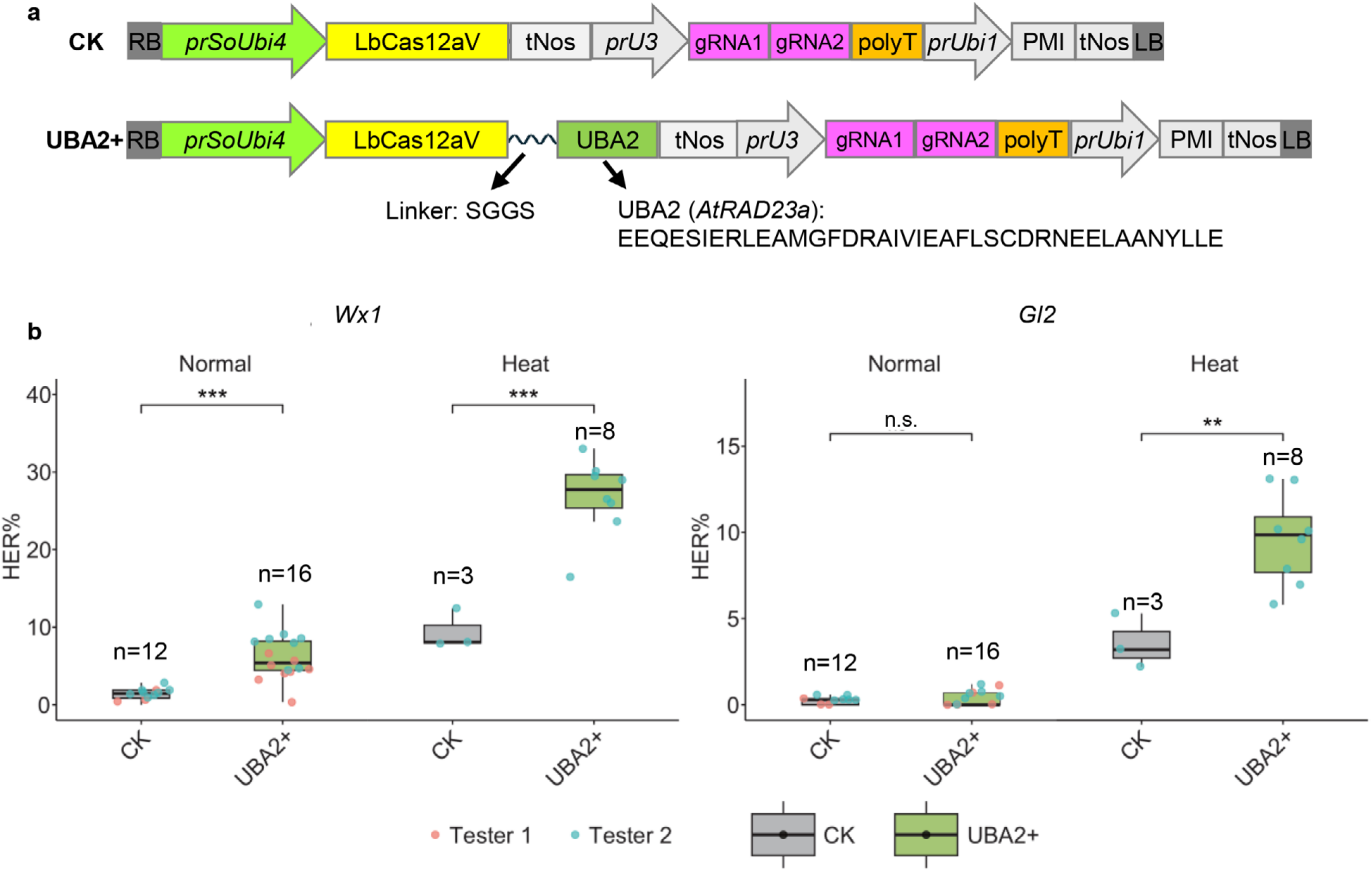
UBA2 fusion boosted haploid editing efficiency. (a) Schematic T‐DNA vectors of UBA2 fusion (UBA2+) and control (CK). LbCas12aV and the C‐terminal UBA2 domain of Arabidopsis RAD23 (*AtRAD23*) are linked by amino acids “SGGS”, driven by sugarcane tetra‐ubiquitin4 promoter (*prSoUbi4*); tNOS, NOS terminator; *prU3*, rice U3 Pol III promoter; gRNA1, guide RNA targeting *Wx1*; gRNA2, guide RNA targeting *Gl2*; *prUbi*, maize ubiquitin promoter; RB/LB, T‐DNA right/left border; PMI (phosphomannose isomerase) was used as a plant selectable marker. (b) The haploid editing rate (HER) of (UBA2+) and control (CK) vectors under normal and heat‐treatment conditions. Sample sizes (n) are indicated above boxes. Different dots indicate different events. Boxplots show medians, quartiles, and whiskers (1.5× IQR). Asterisks denote significant differences (Welch's *t*‐test: *p < 0.05, **p < 0.01, ***p < 0.001, n.s., not significant).

Cas proteins can be rapidly degraded via the ubiquitin‐proteasome system in eukaryotic cells (Chen et al. 2024). In plants, a fusion strategy employing the ubiquitin‐associated domain (UBA) has been used to improve the target protein stability while not compromising protein function (Heessen et al. 2005; Jang et al. 2012). For example, fusing Cas9 with a UBA domain significantly improved editing efficiency in rice (Zheng et al. 2020). We hypothesized that the enhanced Cas protein stability would improve HER in HI‐Edit context because of the narrow editing window before inducer genome elimination. To test the hypotheses, we fused the C‐terminal UBA2 domain of *Arabidopsis thaliana* RAD23 (AtRAD23) to LbCas12aV (vector 28 825). The control vector (27680) expressed unfused LbCas12aV from the same *prSoUbi4* promoter (Figure 4a). The UBA2 fusion vector (28825) was also transformed into haploid inducer SYN‐INBD56; eight (UBA2+) and nine (CK) single‐copy events were evaluated under normal and heat (33°C, 36 h) conditions (Table S10).

Under normal temperature, UBA2 fusion dramatically increased HER^*Wx1*^ compared to CK (Figure 4b). For Tester 1, average HER^*Wx1*^ rose from 0.37% (CK) to 3.97% (UBA2+), a 10‐fold increase. For Tester 2, average HER^*Wx1*^ increased from 1.57% (CK) to 7.81% (UBA2+), a 5‐fold increase. Across both testers, UBA2 fusion improved HER^*Wx1*^ by 6‐fold overall (5.93% vs. 0.98%) and HER^*Gl2*^ by 4.5‐fold (Figure 4b and Table S10).

Heat treatment further amplified the effect of UBA2 fusion. Under heat, the UBA2+ vector gave an average HER^*Wx1*^ of 25.2% (range 16.5%–33%), which was 3‐fold higher than the heat‐treated CK vector and 15‐fold higher than the CK under normal conditions. Similarly, HER^*Gl2*^ reached an average of 8.6% with UBA2+ under heat, representing a 38‐fold improvement over CK at normal temperature (Figure 4b and Table S10). Simultaneous editing of both genes was also highly efficient under the combined treatment (average 7.7% HER^*Wx1* + *Gl2*^). Notably, the maximum HER^*Wx1*^ observed was 33% (event 28 825‐E2, Tester 2), indicating that on average one ear is sufficient to obtain at least two edited haploids.

Zygote editing rates (ZER) followed the same trend. Under heat, UBA2+ events reached ZER^*Wx1*^ values exceeding 75% in several crosses (Table S11). Together, these results demonstrate that combining UBA2‐mediated protein stabilization with a moderate post‐pollination heat treatment provides a robust and scalable HI‐Edit platform, reducing the scale of crosses and cost required to recover edited doubled haploid elite lines.

## 3 Discussion

Haploid induction coupled with genome editing (HI‐Edit) has emerged as a transformative approach to introduce beneficial alleles directly into elite crop germplasm, bypassing the need for trait introgression or direct transformation of recalcitrant varieties (Kelliher et al. 2019; Li, Fu, et al. 2025; Tian et al. 2024). However, the low haploid editing rate (HER) has been a major bottleneck limiting the deployment and scalability of this technology. In this study, we systematically addressed this limitation by optimizing three key factors: (1) the expression level of CRISPR‐Cas in sperm cells and early zygotes, (2) the stability of the Cas protein, and (3) the environmental conditions during fertilization and early embryogenesis. Our combined strategy of using optimized male‐gamete/zygote promoters, post‐pollination heat treatment, and UBA2‐mediated protein stabilization increased HER from below 2% to as high as 33%, representing a 15‐ to 38‐fold improvement depending on the target gene and genetic background.

### 3.1 LbCas12aV Mediated HI‐Edit Through Cell‐Type‐Specific Expressing Editing Machinery

The window of opportunity for HI‐Edit is extremely short—likely less than 48 h and only one or two cell division cycles before the inducer genome is eliminated (Kelliher et al. 2017; Marimuthu et al. 2021). We reasoned that strong expression of the editing machinery in sperm to “carry‐over” and in zygote immediately after fertilization is critical. By mining maize and rice transcriptomic datasets (Anderson et al. 2017; Chen et al. 2017; Hoopes et al. 2019), we identified two promoters, *prZmRZDP* and *prZmVSP*, that drive high expression in sperm cells and early zygotes. These promoters and a constitutive promoter (*prSoUbi4*) driven LbCas12aV conferred efficient HI‐Edit with up to 7.5% HER, which is higher than Cas9 mediated HER (Kelliher et al. 2019; Li, Fu, et al. 2025; Wang et al. 2019). Notably, these promoters also drove strong maternal expression in the zygote, as revealed by reciprocal cross ZER assays (Table S12). This biparental activity may be particularly advantageous because it allows editing of the maternal genome both via CRISPR protein carried over from the sperm and through de novo expression from the zygotic genome before inducer chromosome elimination. Such promoters could also be useful for engineering next‐generation HI‐Edit system or apomixis systems in other crops (Li et al. 2022; Liu et al. 2024).

The Cas enzyme is also important to HI‐Edit. LbCas12a was identified as a potential enzyme for plant editing (Tang et al. 2017). However, the editing activity of wild type LbCas12a in plants was low and could be improved by engineering. In this study, we used an optimized LbCas12a variant‐LbCas12aV, which was engineered previously by adding a long linker and double mutations (D156R‐ Asp to Arg; C965S, Cys to Ser) (Xu et al. 2024). Zhang et al. developed other efficient variants by creating and screening saturation mutagenesis of LbCas12a, in which LbCas12a‐RRV (G146R/D156R/R182V) showed boosted editing efficiency up to 100% in stably transformed rice and poplar plants (Zhang et al. 2023). The plant editing activity of LbCas12a‐RRV could be further improved by inserting introns into the coding sequence (Cheng et al. 2026). These enzymatic optimizations will be beneficial for further improvement of HI‐Edit efficiency.

### 3.2 Heat Treatment: A Simple Environmental Trigger for Robust HI‐Edit

We observed that a transient, mild heat treatment (33°C for 36 h starting 10 h after pollination) substantially increased HER across multiple events and target genes, with improvements of up to 12‐fold at the *Gl2* locus. Several non‐mutually exclusive mechanisms may explain this effect. First, LbCas12a is derived from a mesophilic bacterium with an optimal activity temperature of 35°C–37°C (Banakar et al. 2021; Malzahn et al. 2019); elevating ear temperature likely enhances its enzymatic activity in planta. Second, heat stress is known to increase chromatin accessibility and alter 3D chromatin organization, potentially making target sites more accessible to the editing machinery (Das and Mathur 2023; Huang et al. 2023). Our observation that heat‐treated pollen grains exhibited enlarged sperm nuclei (Figure 3h) is consistent with heat‐induced chromatin relaxation. Third, we detected potential heat shock elements (HSEs) in the *ZmVSP* and *ZmRZDP* promoters, and qRT‐PCR confirmed that heat treatment increased LbCas12aV transcript levels by approximately one‐fold (Figure 3i), suggesting a transcriptional improvement dunder heat treatment. Positive effect of high temperature treatment was also observed in Cas9 mediated genome editing in wheat by using *ZmUbi* promoter (Milner et al. 2020). So, the heat‐treatment could be a universal strategy for improving somatic/haploid editing in plants using Cas9, Cas12a and other editing systems.

Importantly, post‐pollination heat treatment did not adversely affect haploid development or subsequent plant growth (Figure S6e–h). Furthermore, statistical analysis revealed no significant alterations in the haploid induction rate (HIR) between the heat‐treated and control groups (*p* > 0.05; Figure S7). However, excessive heat (≥ 35°C for 36 h) severely reduced seed set, which would offset the gain in HER. By systematically optimizing temperature, duration, and start time, we identified 33°C for 36 h as the best compromise, delivering an average of 2.4 edited haploids per ear—the highest HI‐Edit throughput. This optimized protocol is simple, low‐cost, and readily scalable, making it suitable for both academic research and commercial breeding programs.

### 3.3 UBA2 Fusion Improves Cas Protein Stability and Synergizes With Heat Treatment

Cas proteins are prokaryotic nucleases that can be rapidly degraded by the ubiquitin‐proteasome system in eukaryotic cells (Chen et al. 2024). To counteract this, we fused LbCas12aV with the C‐terminal UBA2 domain of Arabidopsis RAD23, a ubiquitin‐associated domain that protects fusion proteins from proteasomal degradation (Heessen et al. 2005; Jang et al. 2012). Under normal growth conditions, UBA2 fusion increased HER^*Wx1*^ by 6‐fold on average (from 0.98% to 5.93%) and HER^*Gl2*^ by 4.5‐fold. The improvement was even more dramatic when combined with heat treatment: the UBA2+ vector under heat achieved an average HER^*Wx1*^ of 25.2% (up to 33%), representing a 15‐fold increase over the control vector under normal conditions. This level of efficiency dramatically reduces the scale of crosses and embryo handling required and makes it affordable for industry deployments. The quantitative proteomic analysis revealed that the UBA2 fusion significantly increased the accumulation of LbCas12aV protein by ~26% (from 280.72 to 353.67 fmol/μg; Table S13), confirming that enhanced protein stability contributes to the observed HER improvements.

Notably, the UBA2‐fusion effect appeared more significant in HI‐Edit than previously reported for somatic cell editing in rice (Zheng et al. 2020). This is reasonable because the editing window for HI‐Edit is extremely short, making protein stability a particularly limiting factor. In contrast, in stably transformed callus or plants, continuous transcription can compensate for moderate protein instability. Our results therefore suggest that strategies to enhance Cas protein stability are especially valuable for HI‐Edit or cross‐editing applications.

### 3.4 Other Variables and the Utility of ZER as a Pre‐Screening Tool

To investigate whether higher LbCas12aV dosage enhances haploid editing, we compared transgene copy numbers (determined by TaqMan qPCR; Table S2) with HER for 34 independent T0 events across vectors 27 145, 27 146, 27 680, 28 255, 28 291 and 28 292 (Table S5). Copy numbers ranged from 1 to 4, with 28 single‐copy events and six multi‐copy events. Single‐copy events exhibited HER values between 0% and 3.5% with an average of 0.77%, while multi‐copy events showed a broader range (0% to 7.5%) and averaged 2.1% HER. The highest HER (7.5%) was achieved by a multi‐copy event (27146‐E11, four copies), suggesting higher copy events tend to have higher haploid editing efficiency. Notably, there was big variation in HER among the total 42 events across 7 vectors, including some high copy events with no detectable haploid editing (Tables S5, S8, S10), suggesting that insertion site and local chromatin environment play dominant roles, consistent with previous study (Kelliher et al. 2019). The result of flanking sequence analysis revealed a possible correlation between high HER and integration into transcriptionally active genomic regions (gene bodies). As shown in Table S14, the highest‐performing single‐copy event, 27 145‐E5 has its T‐DNA inserted directly within an endogenous gene body on Chromosome 1. Similarly, the multi‐copy event 27 146‐E11 also has its insertion located within a gene body (Chromosome 6). In contrast, events integrated into intergenic, potentially heterochromatic regions (e.g., 27 145‐E2, 27 146‐E5, 27 146‐E8) consistently yielded the lower HER (Table S14), suggesting integration into transcriptionally active gene bodies likely provides a highly accessible chromatin environment and driving active expression of the LbCas12a machinery. Furthermore, we developed ZER assay which showing positive correlation with HER and ZER (*R* ^2^ = 0.70 for Tester 1 and 0.65 for Tester 2; Figure S8). By genotyping as few as 16–48 diploid F1 per event, ZER can be used as a rapid and cheap proxy to pre‐select high‐performing events before committing to large‐scale outcrosses for haploid generation and editing.

### 3.5 Implications for Crop Improvement and Transferability to Other Species

The optimized HI‐Edit framework described here: combining sperm/zygote‐active promoters, post‐pollination heat treatment, and UBA2‐mediated protein stabilization, represents a robust, scalable platform for direct editing of elite maize lines. The maximum HER of 33% achieved at the *Wx1* locus means that an edited haploid can be recovered from less than one cross on average (considering a HIR of ~11%). This efficiency makes HI‐Edit economically viable for both academic laboratories and commercial breeding programs. Moreover, the three strategies target highly conserved biological processes: gamete/zygote gene expression, ubiquitin‐proteasome degradation, and temperature‐dependent enzyme activity and chromatin structure. Therefore, they are likely to be transferable to other crops where haploid induction systems are available or under development.

## 4 Experimental Procedures

### 4.1 Plant Materials and Growth Conditions

The inducer and tester seeds were sown in a 50‐well germination tray and put in a growth chamber with 26°C ± 1°C (day) and 18°C ± 1°C (night), under 300–400 μmol m^−2^ s^−1^ LED light (photoperiod 16:8 (day: night)). V3‐stage seedlings were transplanted into 2‐gal pots filled with substrate and 45 g slow‐release fertilizer (Osmocote 19–6‐12) per pot, the stock plants were grown in a glasshouse with a controlled environment of 25–30 DLI (daily light integral) and 28°C ± 2°C (day) and 18°C ± 2°C (night). HER/ZER trials were conducted in sunlight GH, T1 generation plants were screened for transgene presence using zygosity analysis to identify transgenic‐positive individuals, confirmed transgenic plants and tester plants were transplanted into ground soil supplemented with high‐nitrogen fertilizer, the growth condition in sunlight GH was Day/Night = 25°C–30°C/16°C–20°C, photoperiod was 15 h/9 h (day/night), upon reaching anthesis, crosses were performed using T1 transgenic plants as pollen donors and the testers as the female parent, developing F1 ears were harvested at 4–6 mm embryo stage for subsequent analysis.

### 4.2 Promoter Mining and Vector Construction

Maize RNA‐Seq data (Chen et al. 2017; Dukowic‐Schulze et al. 2014; Hoopes et al. 2019) were analysed. Genes with the highest expression in sperm cells and early zygote (≥ 99th) were screened and candidates were selected (Figure 2a). Selected promoters and terminators were synthesized separately from GenScript (GenScript Biotech Corporation). An intron of Arabidopsis BAF60 homologue was introduced to the maize‐codon optimized Cas12a gene to reduce the toxicity of Cas12a protein to the *E. coli* and Agrobacterium. Cassette expressing gRNAs targeting maize *Wx1* (GRMZM2G024993, gRNA 5′‐GGGAAAGACCGAGGAGAAGATCT‐3′) and *Gl2* (GRMZM2G098239, gRNA 5′‐GTCACAGATCACAAACTTCAAATG‐3′) driven by rice *U3* promoter was synthesized from GenScript as well. A donor sequence of *Gl2* for detecting homologous directed recombination was added in several vectors; it is 859 bp containing 8 bp mutation from ACAAACTT to TAGTGACC in the middle and two protospacers same to the *Gl2* gRNA for donor release. All cassettes were cloned into a binary vector that included a PMI selectable marker. The specific sequence information is provided in Data S1.

### 4.3 Event Generation

Immature embryo of inducer at 9–10 days after pollination was used for transformation. Young embryos of inducer line SYN‐INBD56 were co‐inoculated with *Agrobacterium* strain LBA4404 (pAL4404, pVGW7) containing the binary vectors that express Cas12a and gRNAs targeting *Wx1* and *Gl2*. The transformation with PHOSPHOMANNOSE ISOMERASE (PMI) selection was conducted as described (Zhong et al. 2018).

### 4.4 Event Characterization and Expression Detection

The copy numbers of transgene were detected by targeting the Cas12aV and PMI with a dual plex quantitative real‐time PCR (qPCR) approach, also known as Taqman (Ingham et al. 2001), *ALCOHOL DEHYDROGENASE1* (*ADH1*) was used as the endogenous reference gene (Kelliher et al. 2019). Gene‐specific primers and fluorophore‐labelled probes were designed in Primer Express v3.0 (Applied Biosystems) and synthesized by GenScript. Genomic DNA was extracted from two leaf discs using the Wizard Plant Genomic DNA Extraction Kit (Promega). qPCR was performed on an ABI 7900HT Real‐Time PCR System (Applied Biosystems). Each 10 μL reaction contained 5 μL of 2× qPCR Master Mix (Tiangen), 3 μL of genomic DNA, 0.2 μL of the PMI or Cas12aV TaqMan assay mix (final concentrations: 300 nM primers and 100 nM probe), 0.2 μL of the ADH1 TaqMan assay mix (final concentrations: 300 nM primers and 100 nM probe), and 1.6 μL of nuclease‐free water. Cycling conditions were 95°C for 5 min, followed by 40 cycles of 95°C for 5 s and 60°C for 30 s. Fluorescence signals (FAM and TET) were analysed using SDS v2.4 software (Applied Biosystems), and copy number was calculated using the relative quantitative 2^(‐ΔΔCt) method (Livak and Schmittgen 2001). The same method was used to detect the mutagenesis of target genes through designing and conducting specific Taqman assays targeting *Wx1* and *Gl2* (Kelliher et al. 2019).

The mRNA expression of Cas12aV was detected using a single plex quantitative reverse‐transcriptional real‐time PCR (qRT‐PCR) approach (Abdallah and Bauer 2016) using ENLONGATION FACTOR 1a (EF1a) as the endogenous reference gene. The qPCR reaction mixture comprised reverse transcriptase, 0.3 mM each of forward and reverse primers (Table S10), 0.1 mM of probes, RNA extracted from different tissues, and 2× qPCR Master Mix (Tiangen). Thermal cycling conditions were as follows: reverse transcription at 48°C for 20 min, denaturation at 95°C for 5 min, followed by 40 cycles of 95°C for 5 s for denaturation, and 60°C for 30 s for annealing and extension.

Primers and probes for both copy number, mutagenesis of target genes, and qRT‐PCR analysis were designed utilizing Primer Express 3 software (Applied Biosystems), with details provided in Table S15. Customized fluorescent probes labelled with FAM or TET were synthesized from GenScript (GenScript Biotech Corporation). The PCR samples were loaded onto the 7900HT Fast qPCR system (Applied Biosystems). SDS2.4 software (Applied Biosystems) was used for data analysis.

### 4.5 Haploid/Diploid Identification and Editing Detection

F1 ears were harvested about 16–18 days after outcrossing of Cas‐homo inducer plants with testers. Embryos were extracted and put in Petri dish filled with embryo‐rescue media, the embryos were cultivated in tissue culture room (28°C; 300 μmol m^−2^ s^−1^, 16 h lighting), 24–48 h later the putative haploids and diploids were identified by embryo colour sorting (Figure 1b). Then, haploid or diploid embryos were sampled for genomic DNA extraction and investigated by Taqman assay to detect the transgene presence and mutagenesis of target sites (*Wx1* and *Gl2*). Colour and transgene‐presence sorted haploids were confirmed by flow cytometry (Li et al. 2025). The target gene edited samples were then confirmed by sequencing using PCR amplification and PE250 NGS analysis. Primers for PCR amplification were designed utilizing Geneious Prime software (GraphPad Software LLC) (Table S16). Customized primers were synthesized from GeneWiz (Suzhou GENEWIZ Biotechnology Co. Ltd).

### 4.6 Flow Cytometry

About 200 mg young (5 cm^2^) leaf tissues were sampled and chopped with a sharp blade for 60 s in 500 μL Nuclei Extraction buffer (Sysmex Partec, Muenster, Germany). Samples were then filtered through a 50 μm filter. Afterwards, 2000 μL DAPI buffer was added to each sample and stained for 2 min in the dark. Nuclei suspension was analysed by a CyFlow Space Flow Cytometer (Sysmex Partec, Muenster, Germany) and the corresponding FloMax software. Diploid maize samples were used as controls and the position of their first signal peak was set at ~100 (FL2‐A value). The samples with the first signal peak at ~50 (FL2‐A value) were identified as haploids.

### 4.7 Quantification of Cas12aV Protein

Approximately 100 mg of young corn leaf tissue was harvested, cut into small pieces, and flash‐frozen in liquid nitrogen. The tissues were pulverized using a GenoGrinder at 1100 rpm for 1 min (repeated 3–5 times with intermittent cooling). Total protein was extracted using a plant total protein extraction kit (PE0230, Sigma‐Aldrich) following the manufacturer's instructions. Protein concentration was determined by Bradford assay. For modified Filter‐Aided Sample Preparation (FASP), protein lysates were loaded onto a 10 kDa centrifugal filter. Proteins were reduced with 20 mM dithiothreitol (37°C, 1 h), alkylated with 55 mM iodoacetamide (room temperature, dark, 45 min), washed three times with 50 mM NH_4_HCO_3_, and digested overnight with trypsin (1:50, enzyme‐to‐protein) at 37°C. Peptides were eluted, desalted with C18 StageTips, dried, and resuspended in 0.1% formic acid. Three synthesized isotope‐labelled peptides (NLNNYISLFR, STSIAFR, ELENLEINLR) were spiked as internal standards. Calibration curves were generated using pooled matrix (25–2000 fmol). Samples were analysed on an EASY‐nLC 1200 coupled to an Orbitrap Exploris 240 mass spectrometer. Peptides were separated using a 25‐min linear gradient (20%–100% solvent B). Parallel Reaction Monitoring (PRM) was performed with a full MS scan (300–1500 m/z, resolution 60 000), followed by HCD MS/MS (resolution 30 000, isolation window 1.2 Da, NCE 34.3%). Data were analysed using SpectroDive (v13.4, Biognosys).

### 4.8 Statistical Analysis

Haploid induction rate (HIR), haploid editing rate (HER), zygotic editing rate (ZER) and expression data are presented as box plots showing median values, first and third quartiles, and individual data points. Each experiment was performed with a minimum of three biological replicates, with exact sample sizes indicated in figure legends. Two‐tailed Student's *t*‐tests were performed to compare means between normal and heat treatments, as well as between control (CK) and UBA2+ samples. Statistical significance was set at *p* < 0.05, with significance levels denoted as *p < 0.05, **p < 0.01, ***p < 0.001, and ns for non‐significant differences. Statistical analyses were performed using R Studio (Version 2024.4.2.764).

## Author Contributions

D.L. and T.K. conceptualized the study. J.W. developed and conducted the copy number, target gene mutagenesis, qRT‐PCR, HER, ZER assays, and conducted the data analysis. H.G., H.D. and D.L. developed heat treatment methods, H.G. performed greenhouse plant cultivation, outcrossing, heat‐treatment and F1 ear harvest. T.K., F.Z., Y.Z. and D.L. designed the vectors, Y.J. Y.Z. J.W. and Y.Z. constructed the vectors. J.G. and H.W. mined maize sperm and zygote promoters. X.Z. and Y.L. performed haploid inducer transformation and embryo isolation. H.J. performed T0 event management and outcrossing in Greenhouse. J.W. and W.C. conducted LbCas12 expression assay. Y.J. conducted statistical analysis. J.L., W.S., Q.X. and X.C. provided technical guidance and support. D.L. wrote the manuscript, T.K. and R.E. reviewed and edited the manuscript.

## Conflicts of Interest

T.K. and Qiudeng Que. have filed the patent application PCT/US2017/064512, which describes the HI‐Edit technology, the assignees of PCT/US2017/064512 are Syngenta Participations AG, Basel (CH) and Syngenta Crop Protection LLC, North Carolina (US). J.G., D.L., Wan Shi, J.W., H.J. and T.K. have filed the patent application PCT/CN2023/110941, which covers the maize sperm and zygote promoters for HI‐Edit, the assignees of PCT/CN2023/110941 are Syngenta Crop Protection AG, Basel (CH) and Syngenta Group CO. LTD., Shanghai (CN). D.L., H.G., T.K. and H.D. have filed the patent PCT/CN2023/140616, which covers the heat‐treatment methods after fertilization, UBA2 fusion for the enhancement of haploid editing rate, the assignees of PCT/CN2023/140616 are Syngenta Crop Protection AG, Basel (CH) and Syngenta Group CO. LTD., Shanghai (CN).

## Supporting information


**Figure S1:** Technology concept and advantage of HI‐Edit, compared with conventional process.
**Figure S2:** HI‐Edit promoter validation.
**Figure S3:** Schematic T‐DNA vectors using selected promoters for HI‐Edit test.
**Figure S4:** Zygote editing rate (ZER) on gene targets Wx1 (left), and Gl2 (right) were evaluated by event and tester.
**Figure S5:** The zygote editing rate (ZER) under normal and heat‐treatment conditions.
**Figure S6:** Optimized methods of heat treatment.
**Figure S7:** The haploid induction rate (HIR) showed no significant difference (p > 0.05, n.s., not significant) between normal and heat conditions.
**Figure S8:** Correlation between haploid editing rate (HER) and zygote editing rate (ZER).
**Table S1:** The haploid inducer (SYN‐ INBD56) transformation with LbCas12aV Vectors.
**Table S2:** Copy number analysis of T0 events using Taqman assay.
**Table S3:** Molecular analysis on transgene presence in Putative Haploids (PH*).
**Table S4:** The Haploid Induction Rate (HIR) % of inducer (SYN‐ INBD56) events transformed by LbCas12aV Vectors.
**Table S5:** The haploid editing rate (HER) and zygote editing rate (ZER) of HI‐Edit vectors and events.
**Table S6:** Genotype of target genes in edited haploids by NGS.
**Table S7:** Genotype of target genes in edited F1 diploids.
**Table S8:** The haploid editing rate (HER) under normal and heat treatment conditions.
**Table S9:** The zygote editing rate (ZER) under normal and heat treatment conditions.
**Table S10:** The haploid editing rate (HER) of UBA2 fusion and control vector (27680) under normal and heat treatment conditions.
**Table S11:** The zygote editing rate (ZER) of UBA2 fusion and control vector under normal and heat treatment conditions.
**Table S12:** The ZER assay of reciprocal crosses.
**Table S13:** Quantification of Cas12aV protein in leaf samples from UBA2 fusion and control (CK) vectors.
**Table S14:** HER for different events with different copy number and insertion sites in the genome.
**Table S15:** Primers and probes of Taqman assays used for copy number of events, LbCas12aV expression and absence of CRISPR reagents introgression in haploid samples.
**Table S16:** Primers used for amplifying target genes.

**Data S1:** The specific sequence information.

## Data Availability

The data that supports the findings of this study are available in the Data S1 of this article.
