# Supplementary material for: A Robust Framework for Maize Elite Line Genome Editing Through Enhanced HI‐Edit via LbCas12a Activity Optimization
Source: Plant Biotechnol J. 2026 Jul 4;24(10):5392–404. doi: 10.1111/pbi.70715 (PMC13399594; doi:10.1111/pbi.70715)
Supplement: Supplementary file 2 [file PBI-24-5392-s002.docx]

**Appendix. Sequences of key components used for vector construction.**

>e35S

ACTTTTCAACAAAGGGTATTATCCGGAAACCTCCTCGGATTCCATTGCCCAGCTATCTGTCACTTTATTGTGAAGATAGTGGAAAAGGAAGGTGGCTCCTACAAATGCCATCATTGCGATAAAGGAAAGGCTATCGTTGAAGATGCCTCTGCCGACAGTGGTCCCAAAGATGGACCCCCACCCACGAGGAGCATCGTGGAAAAAGAAGACGTTCCAACCACGTCTTCAAAGCAAGTGGATTGATGTGATATCTCCACTGACGTAAGGGTTGACGAACAATCCCACTATCCTTC

>eBSV

GCCAGAAGATAGAAGATATCCTGGACCTGCAAGATGTCAGCAATGACGATTGAAAGATTCCCAGGATAGCCGGCGGACGTGGTGGACCCAGTCTAGGTGCGATGCTTAGTCACGCACGATGACTCTGTCGGAAGGCATCTTTACTTTCGGCAAACTTTAATAATACTTTAGGAAAAGTATTGTACAAGTTAGGTGCAGAATCAATAATGCACCCAGCTTTAGTCTTGTCTACTGAATTATTGTGTCGGTTGCATTATTGGATGCCTGCGTGCACCCTAAGCAATCCCCGG

>eFMV

AGCTGCTTGTGGGGACCAGACAAAAAAGGAATGGTGCAGAATTGTTAGGCGCACCTACCAAAAGCAACTTTGCCTTTATTGCAAAGATAAAGCAGATTCCTCTAGTACAAGTGGGGAACAAAATAACGTGGAAAAGAGCTGTCCTGACAGCCCACTCACTATTGCGTTTGACGAACGCAGTGACGACCACAAAA

>eMMV

GGATTAATGGATTGATCAACATCCTTACCGCTATGGGTAAGATTGATGAAAAGTCAAAAACAAAAATCAATTATGCACACCAGCATGTGTTGATCACCAGCTATTGTGGGACACCAATTTCGTCCACAGACATCAACATCTTATCGTCCTTTGAAGATAAGATAATAATGTTGAAGATAAGAGTGGGAGCCACCACTAAAACATTGCTTTGTCAAAAGCTAAAAAAGATGATGCCCGACAGCCACTTGTGTGAAGCATGTGAAGCCGGTCCCTCCACTAAGAAAATTAGTGAAGCATCTTCCAGTGGTCCCTCCACTCACAGCTCAATCAGTGAGCAACAGGACGAAGGAAATGACGTAAGCCATGACGTCTAATCCCACA

>LbCas12aV

ATGCCGAAGAAGAAGCGCAAGGTCGGGGGCGGGGGCTCAGGCGGGGGCGGGAGCGGCGGCGGGGGCTCTGGGGGCGGCGGCAGCGGCGGGGGCGGCAGCGGGGGCGGCGGGTCGATGAGCAAGCTGGAGAAGTTCACGAACTGCTACTCCCTCAGCAAGACCCTGAGGTTCAAGGCGATCCCGGTCGGCAAGACCCAGGAGAACATCGACAACAAGCGGCTGCTGGTGGAGGACGAGAAGAGGGCTGAGGACTACAAGGGCGTGAAGAAGCTCCTGGACCGCTACTACCTGTCCTTCATCAACGACGTGCTCCACAGCATCAAGCTCAAGAACCTGAACAACTACATCAGCCTCTTCAGGAAGAAGACGCGCACCGAGAAGGAGAACAAGGAGCTCGAGAACCTGGAGATCAACCTGAGGAAGGAGATCGCCAAGGCGTTCAAGGGCAACGAGGGCTACAAGTCCCTCTTCAAGAAGGACATCATCGAGACGATCCTCCCGGAGTTCCTGGACGACAAGGACGAGATCGCCCTGGTCAACTCCTTCAACGGCTTCACCACGGCGTTCACCGGCTTCTTCAGGAACCGCGAGAACATGTTCAGCGAGGAGGCCAAGTCCACGAGCATCGCGTTCAGGTACCAAGCTGCGAATCTTCGTTTTTTTAAGGAATTCTCGATCTTTATGGTGTATAGGCTCTGGGTTTTCTGTTTTTTGTATCTCTTAGGATTTTGTAAATTCCAGATCTTTCTATGGCCACTTAGTAGTATATTTCAAAAATTCTCCAATCGAGTTCTTCATTCGCATTTTCAGTCATTTTCTCTTCGACGTTGTTTTTAAGCCTGGGTATTACTCCTATTTAGTTGAACTCTGCAGCAATCTTAGAAAATTAGGGTTTTGAGGTTTCGATTTCTCTAGGTAACCGATCTATTGCATTCATCTGAATTTCTGCATATATGTCTTAGATTTCTGATAAGCTTACGATACGTTAGGTGTAATTGAAGTTTATTTTTCAAGAGTGTTATTTTTTGTTTCTGAATTTTTCAGGTGCATCAACGAGAACCTCACCCGCTACATCTCCAACATGGACATCTTCGAGAAGGTCGACGCGATCTTCGACAAGCACGAGGTGCAGGAGATCAAGGAGAAGATCCTGAACAGCGACTACGACGTCGAGGACTTCTTCGAGGGCGAGTTCTTCAACTTCGTCCTCACGCAGGAGGGCATCGACGTGTACAACGCCATCATCGGTGGCTTCGTGACCGAGTCCGGCGAGAAGATCAAGGGCCTGAACGAGTACATCAACCTCTACAACCAGAAGACCAAGCAGAAGCTGCCGAAGTTCAAGCCCCTGTACAAGCAGGTGCTCTCCGACAGGGAGTCCCTCAGCTTCTACGGCGAGGGCTACACGAGCGACGAGGAGGTCCTGGAGGTGTTCCGCAACACCCTCAACAAGAACAGCGAGATCTTCTCCAGCATCAAGAAGCTCGAGAAGCTGTTCAAGAACTTCGACGAGTACTCCAGCGCCGGCATCTTCGTCAAGAACGGCCCGGCGATCTCCACGATCAGCAAGGACATCTTCGGCGAGTGGAACGTGATCCGCGACAAGTGGAACGCCGAGTACGACGACATCCACCTCAAGAAGAAGGCGGTGGTCACCGAGAAGTACGAGGACGACAGGCGCAAGTCCTTCAAGAAGATCGGCTCCTTCAGCCTCGAGCAGCTGCAGGAGTACGCCGACGCGGACCTGAGCGTGGTCGAGAAGCTCAAGGAGATCATCATCCAGAAGGTCGACGAGATCTACAAGGTGTACGGCTCCAGCGAGAAGCTCTTCGACGCGGACTTCGTCCTCGAGAAGTCCCTGAAGAAGAACGACGCCGTGGTCGCGATCATGAAGGACCTCCTGGACTCCGTGAAGAGCTTCGAGAATTACATCAAGGCCTTCTTCGGCGAGGGCAAGGAGACGAACAGGGACGAGTCCTTCTACGGCGACTTCGTCCTGGCCTACGACATCCTCCTGAAGGTGGACCACATCTACGACGCGATCCGCAACTACGTGACCCAGAAGCCGTACAGCAAGGACAAGTTCAAGCTCTACTTCCAGAACCCCCAGTTCATGGGCGGCTGGGACAAGGACAAGGAGACGGACTACAGGGCGACCATCCTGCGCTACGGCAGCAAGTACTACCTCGCCATCATGGACAAGAAGTACGCGAAGTGCCTGCAGAAGATCGACAAGGACGACGTCAACGGCAACTACGAGAAGATCAACTACAAGCTCCTGCCGGGCCCCAACAAGATGCTCCCGAAGGTGTTCTTCTCCAAGAAGTGGATGGCCTACTACAACCCCAGCGAGGACATCCAGAAGATCTACAAGAACGGCACGTTCAAGAAGGGCGACATGTTCAACCTGAACGACTGCCACAAGCTCATCGACTTCTTCAAGGACTCCATCAGCCGCTACCCGAAGTGGTCCAACGCCTACGACTTCAACTTCAGCGAGACCGAGAAGTACAAGGACATCGCGGGCTTCTACCGCGAGGTCGAGGAGCAGGGCTACAAGGTGTCCTTCGAGTCCGCCAGCAAGAAGGAGGTCGACAAGCTGGTGGAGGAGGGCAAGCTCTACATGTTCCAGATCTACAACAAGGACTTCTCCGACAAGAGCCACGGCACGCCCAACCTGCACACCATGTACTTCAAGCTCCTGTTCGACGAGAACAACCACGGCCAGATCAGGCTGTCCGGCGGCGCCGAGCTCTTCATGAGGAGGGCGAGCCTGAAGAAGGAGGAGCTGGTGGTCCACCCCGCTAACAGCCCAATCGCGAACAAGAACCCGGACAACCCCAAGAAGACCACGACCCTGTCCTACGACGTGTACAAGGACAAGAGGTTCAGCGAGGACCAGTACGAGCTCCACATCCCGATCGCGATCAACAAGTGCCCCAAGAACATCTTCAAGATCAACACCGAGGTCCGCGTGCTCCTGAAGCACGACGACAACCCCTACGTGATCGGCATCGACAGGGGCGAGAGGAACCTCCTGTACATCGTGGTCGTGGACGGCAAGGGCAACATCGTGGAGCAGTACTCCCTCAACGAGATCATCAACAACTTCAACGGCATCAGGATCAAGACGGACTACCACAGCCTCCTGGACAAGAAGGAGAAGGAGAGGTTCGAGGCCCGCCAGAACTGGACCTCCATCGAGAACATCAAGGAGCTGAAGGCGGGCTACATCAGCCAGGTCGTGCACAAGATCTGCGAGCTCGTCGAGAAGTACGACGCCGTGATCGCCCTCGAGGACCTGAACTCCGGCTTCAAGAACAGCCGCGTCAAGGTGGAGAAGCAGGTCTACCAGAAGTTCGAGAAGATGCTCATCGACAAGCTGAACTACATGGTGGACAAGAAGTCCAACCCCTCCGCTACGGGCGGCGCGCTGAAGGGCTACCAGATCACCAACAAGTTCGAGAGCTTCAAGTCCATGAGCACTCAGAACGGCTTCATCTTCTACATCCCGGCGTGGCTCACGTCCAAGATCGACCCCAGCACCGGCTTCGTCAACCTCCTGAAGACGAAGTACACCTCCATCGCCGACAGCAAGAAGTTCATCTCCAGCTTCGACCGCATCATGTATGTGCCGGAGGAGGACCTGTTCGAGTTCGCCCTCGACTACAAGAACTTCTCCCGCACGGACGCGGACTACATCAAGAAGTGGAAGCTGTACAGCTACGGCAACCGCATCCGCATCTTCAGGAACCCCAAGAAGAACAACGTCTTCGACTGGGAGGAGGTGTGCCTGACCTCCGCGTACAAGGAGCTCTTCAACAAGTACGGCATCAACTACCAGCAGGGCGACATCAGGGCTCTCCTGTGCGAGCAGAGCGACAAGGCCTTCTACTCCAGCTTCATGGCGCTGATGTCCCTCATGCTGCAGATGAGGAACTCGATCACCGGCAGGACGGACGTGGACTTCCTCATCTCCCCGGTGAAGAACAGCGACGGCATCTTCTACGACTCCAGGAACTACGAGGCCCAGGAGAACGCGATCCTCCCAAAGAACGCGGACGCCAACGGCGCCTACAACATCGCCAGGAAGGTCCTCTGGGCTATCGGCCAGTTCAAGAAGGCGGAGGACGAGAAGCTGGACAAGGTGAAGATCGCCATCAGCAACAAGGAGTGGCTCGAGTACGCCCAGACCTCGGTCAAGCACGGCAGCCCGAAGAAGAAGCGCAAGGTGTCCGGCGGCAGCTCCGGCGGCAGCCCGAAGAAGAAGCGCAAAGTGTGA

>LbCas12a-UBA2

ATGCCGAAGAAGAAGCGCAAGGTCGGGGGCGGGGGCTCAGGCGGGGGCGGGAGCGGCGGCGGGGGCTCTGGGGGCGGCGGCAGCGGCGGGGGCGGCAGCGGGGGCGGCGGGTCGATGAGCAAGCTGGAGAAGTTCACGAACTGCTACTCCCTCAGCAAGACCCTGAGGTTCAAGGCGATCCCGGTCGGCAAGACCCAGGAGAACATCGACAACAAGCGGCTGCTGGTGGAGGACGAGAAGAGGGCTGAGGACTACAAGGGCGTGAAGAAGCTCCTGGACCGCTACTACCTGTCCTTCATCAACGACGTGCTCCACAGCATCAAGCTCAAGAACCTGAACAACTACATCAGCCTCTTCAGGAAGAAGACGCGCACCGAGAAGGAGAACAAGGAGCTCGAGAACCTGGAGATCAACCTGAGGAAGGAGATCGCCAAGGCGTTCAAGGGCAACGAGGGCTACAAGTCCCTCTTCAAGAAGGACATCATCGAGACGATCCTCCCGGAGTTCCTGGACGACAAGGACGAGATCGCCCTGGTCAACTCCTTCAACGGCTTCACCACGGCGTTCACCGGCTTCTTCAGGAACCGCGAGAACATGTTCAGCGAGGAGGCCAAGTCCACGAGCATCGCGTTCAGGTACCAAGCTGCGAATCTTCGTTTTTTTAAGGAATTCTCGATCTTTATGGTGTATAGGCTCTGGGTTTTCTGTTTTTTGTATCTCTTAGGATTTTGTAAATTCCAGATCTTTCTATGGCCACTTAGTAGTATATTTCAAAAATTCTCCAATCGAGTTCTTCATTCGCATTTTCAGTCATTTTCTCTTCGACGTTGTTTTTAAGCCTGGGTATTACTCCTATTTAGTTGAACTCTGCAGCAATCTTAGAAAATTAGGGTTTTGAGGTTTCGATTTCTCTAGGTAACCGATCTATTGCATTCATCTGAATTTCTGCATATATGTCTTAGATTTCTGATAAGCTTACGATACGTTAGGTGTAATTGAAGTTTATTTTTCAAGAGTGTTATTTTTTGTTTCTGAATTTTTCAGGTGCATCAACGAGAACCTCACCCGCTACATCTCCAACATGGACATCTTCGAGAAGGTCGACGCGATCTTCGACAAGCACGAGGTGCAGGAGATCAAGGAGAAGATCCTGAACAGCGACTACGACGTCGAGGACTTCTTCGAGGGCGAGTTCTTCAACTTCGTCCTCACGCAGGAGGGCATCGACGTGTACAACGCCATCATCGGTGGCTTCGTGACCGAGTCCGGCGAGAAGATCAAGGGCCTGAACGAGTACATCAACCTCTACAACCAGAAGACCAAGCAGAAGCTGCCGAAGTTCAAGCCCCTGTACAAGCAGGTGCTCTCCGACAGGGAGTCCCTCAGCTTCTACGGCGAGGGCTACACGAGCGACGAGGAGGTCCTGGAGGTGTTCCGCAACACCCTCAACAAGAACAGCGAGATCTTCTCCAGCATCAAGAAGCTCGAGAAGCTGTTCAAGAACTTCGACGAGTACTCCAGCGCCGGCATCTTCGTCAAGAACGGCCCGGCGATCTCCACGATCAGCAAGGACATCTTCGGCGAGTGGAACGTGATCCGCGACAAGTGGAACGCCGAGTACGACGACATCCACCTCAAGAAGAAGGCGGTGGTCACCGAGAAGTACGAGGACGACAGGCGCAAGTCCTTCAAGAAGATCGGCTCCTTCAGCCTCGAGCAGCTGCAGGAGTACGCCGACGCGGACCTGAGCGTGGTCGAGAAGCTCAAGGAGATCATCATCCAGAAGGTCGACGAGATCTACAAGGTGTACGGCTCCAGCGAGAAGCTCTTCGACGCGGACTTCGTCCTCGAGAAGTCCCTGAAGAAGAACGACGCCGTGGTCGCGATCATGAAGGACCTCCTGGACTCCGTGAAGAGCTTCGAGAATTACATCAAGGCCTTCTTCGGCGAGGGCAAGGAGACGAACAGGGACGAGTCCTTCTACGGCGACTTCGTCCTGGCCTACGACATCCTCCTGAAGGTGGACCACATCTACGACGCGATCCGCAACTACGTGACCCAGAAGCCGTACAGCAAGGACAAGTTCAAGCTCTACTTCCAGAACCCCCAGTTCATGGGCGGCTGGGACAAGGACAAGGAGACGGACTACAGGGCGACCATCCTGCGCTACGGCAGCAAGTACTACCTCGCCATCATGGACAAGAAGTACGCGAAGTGCCTGCAGAAGATCGACAAGGACGACGTCAACGGCAACTACGAGAAGATCAACTACAAGCTCCTGCCGGGCCCCAACAAGATGCTCCCGAAGGTGTTCTTCTCCAAGAAGTGGATGGCCTACTACAACCCCAGCGAGGACATCCAGAAGATCTACAAGAACGGCACGTTCAAGAAGGGCGACATGTTCAACCTGAACGACTGCCACAAGCTCATCGACTTCTTCAAGGACTCCATCAGCCGCTACCCGAAGTGGTCCAACGCCTACGACTTCAACTTCAGCGAGACCGAGAAGTACAAGGACATCGCGGGCTTCTACCGCGAGGTCGAGGAGCAGGGCTACAAGGTGTCCTTCGAGTCCGCCAGCAAGAAGGAGGTCGACAAGCTGGTGGAGGAGGGCAAGCTCTACATGTTCCAGATCTACAACAAGGACTTCTCCGACAAGAGCCACGGCACGCCCAACCTGCACACCATGTACTTCAAGCTCCTGTTCGACGAGAACAACCACGGCCAGATCAGGCTGTCCGGCGGCGCCGAGCTCTTCATGAGGAGGGCGAGCCTGAAGAAGGAGGAGCTGGTGGTCCACCCCGCTAACAGCCCAATCGCGAACAAGAACCCGGACAACCCCAAGAAGACCACGACCCTGTCCTACGACGTGTACAAGGACAAGAGGTTCAGCGAGGACCAGTACGAGCTCCACATCCCGATCGCGATCAACAAGTGCCCCAAGAACATCTTCAAGATCAACACCGAGGTCCGCGTGCTCCTGAAGCACGACGACAACCCCTACGTGATCGGCATCGACAGGGGCGAGAGGAACCTCCTGTACATCGTGGTCGTGGACGGCAAGGGCAACATCGTGGAGCAGTACTCCCTCAACGAGATCATCAACAACTTCAACGGCATCAGGATCAAGACGGACTACCACAGCCTCCTGGACAAGAAGGAGAAGGAGAGGTTCGAGGCCCGCCAGAACTGGACCTCCATCGAGAACATCAAGGAGCTGAAGGCGGGCTACATCAGCCAGGTCGTGCACAAGATCTGCGAGCTCGTCGAGAAGTACGACGCCGTGATCGCCCTCGAGGACCTGAACTCCGGCTTCAAGAACAGCCGCGTCAAGGTGGAGAAGCAGGTCTACCAGAAGTTCGAGAAGATGCTCATCGACAAGCTGAACTACATGGTGGACAAGAAGTCCAACCCCTCCGCTACGGGCGGCGCGCTGAAGGGCTACCAGATCACCAACAAGTTCGAGAGCTTCAAGTCCATGAGCACTCAGAACGGCTTCATCTTCTACATCCCGGCGTGGCTCACGTCCAAGATCGACCCCAGCACCGGCTTCGTCAACCTCCTGAAGACGAAGTACACCTCCATCGCCGACAGCAAGAAGTTCATCTCCAGCTTCGACCGCATCATGTATGTGCCGGAGGAGGACCTGTTCGAGTTCGCCCTCGACTACAAGAACTTCTCCCGCACGGACGCGGACTACATCAAGAAGTGGAAGCTGTACAGCTACGGCAACCGCATCCGCATCTTCAGGAACCCCAAGAAGAACAACGTCTTCGACTGGGAGGAGGTGTGCCTGACCTCCGCGTACAAGGAGCTCTTCAACAAGTACGGCATCAACTACCAGCAGGGCGACATCAGGGCTCTCCTGTGCGAGCAGAGCGACAAGGCCTTCTACTCCAGCTTCATGGCGCTGATGTCCCTCATGCTGCAGATGAGGAACTCGATCACCGGCAGGACGGACGTGGACTTCCTCATCTCCCCGGTGAAGAACAGCGACGGCATCTTCTACGACTCCAGGAACTACGAGGCCCAGGAGAACGCGATCCTCCCAAAGAACGCGGACGCCAACGGCGCCTACAACATCGCCAGGAAGGTCCTCTGGGCTATCGGCCAGTTCAAGAAGGCGGAGGACGAGAAGCTGGACAAGGTGAAGATCGCCATCAGCAACAAGGAGTGGCTCGAGTACGCCCAGACCTCGGTCAAGCACGGCAGCTCCGGCGGCAGCGAAGAGCAGGAGTCTATCGAGAGGCTCGAGGCCATGGGCTTCGACAGGGCTATCGTGATCGAGGCCTTCCTGTCTTGCGATAGGAACGAGGAGCTGGCTGCCAACTACCTCCTGGAGCCGAAGAAGAAGCGCAAGGTGTCCGGCGGCAGCTCCGGCGGCAGCCCGAAGAAGAAGCGCAAAGTGTGA

>prAct1

TACTCGAGGTCATTCATATGCTTGAGAAGAGAGTCGGGATAGTCCAAAATAAAACAAAGGTAAGATTACCTGGTCAAAAGTGAAAACATCAGTTAAAAGGTGGTATAAAGTAAAATATCGGTAATAAAAGGTGGCCCAAAGTGAAATTTACTCTTTTCTACTATTATAAAAATTGAGGATGTTTTTGTCGGTACTTTGATACGTCATTTTTGTATGAATTGGTTTTTAAGTTTATTCGCTTTTGGAAATGCATATCTGTATTTGAGTCGGGTTTTAAGTTCGTTTGCTTTTGTAAATACAGAGGGATTTGTATAAGAAATATCTTTAGAAAAACCCATATGCTAATTTGACATAATTTTTGAGAAAAATATATATTCAGGCGAATTCTCACAATGAACAATAATAAGATTAAAATAGCTTTCCCCCGTTGCAGCGCATGGGTATTTTTTCTAGTAAAAATAAAAGATAAACTTAGACTCAAAACATTTACAAAAACAACCCCTAAAGTTCCTAAAGCCCAAAGTGCTATCCACGATCCATAGCAAGCCCAGCCCAACCCAACCCAACCCAACCCACCCCAGTCCAGCCAACTGGACAATAGTCTCCACACCCCCCCACTATCACCGTGAGTTGTCCGCACGCACCGCACGTCTCGCAGCCAAAAAAAAAAAGAAAGAAAAAAAAGAAAAAGAAAAAACAGCAGGTGGGTCCGGGTCGTGGGGGCCGGAAACGCGAGGAGGATCGCGAGCCAGCGACGAGGCCGGCCCTCCCTCCGCTTCCAAAGAAACGCCCCCCATCGCCACTATATACATACCCCCCCCTCTCCTCCCATCCCCCCAACCCTACCACCACCACCACCACCACCTCCACCTCCTCCCCCCTCGCTGCCGGACGACGAGCTCCTCCCCCCTCCCCCTCCGCCGCCGCCGCGCCGGTAACCACCCCGCCCCTCTCCTCTTTCTTTCTCCGTTTTTTTTTCCGTCTCGGTCTCGATCTTTGGCCTTGGTAGTTTGGGTGGGCGAGAGGCGGCTTCGTGCGCGCCCAGATCGGTGCGCGGGAGGGGCGGGATCTCGCGGCTGGGGCTCTCGCCGGCGTGGATCCGGCCCGGATCTCGCGGGGAATGGGGCTCTCGGATGTAGATCTGCGATCCGCCGTTGTTGGGGGAGATGATGGGGGGTTTAAAATTTCCGCCGTGCTAAACAAGATCAGGAAGAGGGGAAAAGGGCACTATGGTTTATATTTTTATATATTTCTGCTGCTTCGTCAGGCTTAGATGTGCTAGATCTTTCTTTCTTCTTTTTGTGGGTAGAATTTGAATCCCTCAGCATTGTTCATCGGTAGTTTTTCTTTTCATGATTTGTGACAAATGCAGCCTCGTGCGGAGCTTTTTTGTAGGTAGAAG>prOsU3GGGATCTTTAAACATACGAACAGATCACTTAAAGTTCTTCTGAAGCAACTTAAAGTTATCAGGCATGCATGGATCTTGGAGGAATCAGATGTGCAGTCAGGGACCATAGCACAGGACAGGCGTCTTCTACTGGTGCTACCAGCAAATGCTGGAAGCCGGGAACACTGGGTACGTTGGAAACCACGTGATGTGGAGTAAGATAAACTGTAGGAGAAAAGCATTTCGTAGTGGGCCATGAAGCCTTTCAGGACATGTATTGCAGTATGGGCCGGCCCATTACGCAATTGGACGACAACAAAGACTAGTATTAGTACCACCTCGGCTATCCACATAGATCAAAGCTGGTTTAAAAGAGTTGTGCAGATGATCCGTGGC

>prSoUbi4

CATTATGTGGTCTAGGTAGGTTCTATATATAAGAAAACTTGAAATGTTCTAAAAAAAAATTCAAGCCCATGCATGATTGAAGCAAACGGTATAGCAACGGTGTTAACCTGATCTAGTGATCTCTTGCAATCCTTAACGGCCACCTACCGCAGGTAGCAAACGGCGTCCCCCTCCTCGATATCTCCGCGGCGACCTCTGGCTTTTTCCGCGGAATTGCGCGGTGGGGACGGATTCCACGAGACCGCGACGCAACCGCCTCTCGCCGCTGGGCCCCACACCGCTCGGTGCCGTAGCCTCACGGGACTCTTTCTCCCTCCTCCCCCGTTATAAATTGGCTTCATCCCCTCCTTGCCTCATCCATCCAAATCCCAGTCCCCAATCCCATCCCTTCGTAGGAGAAATTCATCGAAGCTAAGCGAATCCTCGCGATCCTCTCAAGGTACTGCGAGTTTTCGATCCCCCTCTCGACCCCTCGTATGTTTGTGTTTGTCGTAGCGTTTGATTAGGTATGCTTTCCCTGTTTGTGTTCGTCGTAGCGTTTGATTAGGTATGCTTTCCCTGTTCGTGTTCATCGTAGTGTTTGATTAGGTCGTGTGAGGCGATGGCCTGCTCGCGTCCTTCGATCTGTAGTCGATTTGCGGGTCGTGGTGTAGATCTGCGGGCTGTGATGAAGTTATTTGGTGTGATCTGCTCGCCTGATTCTGCGGGTTGGCTCGAGTAGATATGATGGTTGGACCGGTTGGTTCGTTTACCGCGCTAGGGTTGGGCTGGGATGATGTTGCATGCGCCGTTGCGCGTGATCCCGCAGCAGGACTTGCGTTTGATTGCCAGATCTCGTTACGATTATGTGATTTGGTTTGGACTTTTTAGATCTGTAGCTTCTGCTTATGTGCCAGATGCGCCTACTGCTCATATGCCTGATGATAATCATAAATGGCTGTGGAACTAACTAGTTGATTGCGGAGTCATGTATCAGCTACAGGTGTAGGGACTAGCTACAGGTGTAGGGACTTGCGTCTAATTGTTTGGTCCTTTACTCATGTTGCAATTATGCAATTTAGTTTAGATTGTTTGTTCCACTCATCTAGGCTGTAAAAGGGACACTGCTTAGATTGCTGTTTAATCTTTTTAGTAGATTATATTATATTGGTAACTTATTACCCCTATTACATGCCATACGTGACTTCTGCTCATGCCTGATGATAATCATAGATCACTGTGGAATTAATTAGTTGATTGTTGAATCATGTTTCATGTACATACCACGGCACAATTGCTTAGTTCCTTAACAAATGCAAATTTTACTGATCCATGTATGATTTGCGTGGTTCTCTAATGTGAAATACTATAGCTACTTGTTAGTAAGAATCAGGTTCGTATGCTTAATGCTGTATGTGCCTTCTGCTCATGCCTGATGATAATCATATATCACTGGAATTAATTAGTTGATCGTTTAATCATATATCAAGTACATACCATGCCACAATTTTTAGTCACTTAACCCATGCAGATTGAACTGGTCCCTGCATGTTTTGCTAAATTGTTCTATTCTGATTAGACCATATATCATGTATTTTTTTTTGGTAATGGTTCTCTTATTTTAAATGCTATATAGTTCTGGTACTTGTTAGAAAGATCTGCTTCATAGTTTAGTTGCCTATCCCTCGAATTAGGATGCTGAGCAGCTGATCCTATAGCTTTGTTTCATGTATCAATTCTTTTGTGTTCAACAGTCAGTTTTTGTTAGATTCATTGTAACTTATGGTCGCTTACTCTTCTGGTCCTCAATGCTTGCAG

>prUbi1

CTGCAGTGCAGCGTGACCCGGTCGTGCCCCTCTCTAGAGATAATGAGCATTGCATGTCTAAGTTATAAAAAATTACCACATATTTTTTTTGTCACACTTGTTTGAAGTGCAGTTTATCTATCTTTATACATATATTTAAACTTTACTCTACGAATAATATAATCTATAGTACTACAATAATATCAGTGTTTTAGAGAATCATATAAATGAACAGTTAGACATGGTCTAAAGGACAATTGAGTATTTTGACAACAGGACTCTACAGTTTTATCTTTTTAGTGTGCATGTGTTCTCCTTTTTTTTTGCAAATAGCTTCACCTATATAATACTTCATCCATTTTATTAGTACATCCATTTAGGGTTTAGGGTTAATGGTTTTTATAGACTAATTTTTTTAGTACATCTATTTTATTCTATTTTAGCCTCTAAATTAAGAAAACTAAAACTCTATTTTAGTTTTTTTATTTAATAATTTAGATATAAAATAGAATAAAATAAAGTGACTAAAAATTAAACAAATACCCTTTAAGAAATTAAAAAAACTAAGGAAACATTTTTCTTGTTTCGAGTAGATAATGCCAGCCTGTTAAACGCCGTCGACGAGTCTAACGGACACCAACCAGCGAACCAGCAGCGTCGCGTCGGGCCAAGCGAAGCAGACGGCACGGCATCTCTGTCGCTGCCTCTGGACCCCTCTCGAGAGTTCCGCTCCACCGTTGGACTTGCTCCGCTGTCGGCATCCAGAAATTGCGTGGCGGAGCGGCAGACGTGAGCCGGCACGGCAGGCGGCCTCCTCCTCCTCTCACGGCACCGGCAGCTACGGGGGATTCCTTTCCCACCGCTCCTTCGCTTTCCCTTCCTCGCCCGCCGTAATAAATAGACACCCCCTCCACACCCTCTTTCCCCAACCTCGTGTTGTTCGGAGCGCACACACACACAACCAGATCTCCCCCAAATCCACCCGTCGGCACCTCCGCTTCAAGGTACGCCGCTCGTCCTCCCCCCCCCCCCCTCTCTACCTTCTCTAGATCGGCGTTCCGGTCCATGGTTAGGGCCCGGTAGTTCTACTTCTGTTCATGTTTGTGTTAGATCCGTGTTTGTGTTAGATCCGTGCTGCTAGCGTTCGTACACGGATGCGACCTGTACGTCAGACACGTTCTGATTGCTAACTTGCCAGTGTTTCTCTTTGGGGAATCCTGGGATGGCTCTAGCCGTTCCGCAGACGGGATCGATTTCATGATTTTTTTTGTTTCGTTGCATAGGGTTTGGTTTGCCCTTTTCCTTTATTTCAATATATGCCGTGCACTTGTTTGTCGGGTCATCTTTTCATGCTTTTTTTTGTCTTGGTTGTGATGATGTGGTCTGGTTGGGCGGTCGTTCTAGATCGGAGTAGAATTCTGTTTCAAACTACCTGGTGGATTTATTAATTTTGGATCTGTATGTGTGTGCCATACATATTCATAGTTACGAATTGAAGATGATGGATGGAAATATCGATCTAGGATAGGTATACATGTTGATGCGGGTTTTACTGATGCATATACAGAGATGCTTTTTGTTCGCTTGGTTGTGATGATGTGGTGTGGTTGGGCGGTCGTTCATTCGTTCTAGATCGGAGTAGAATACTGTTTCAAACTACCTGGTGTATTTATTAATTTTGGAACTGTATGTGTGTGTCATACATCTTCATAGTTACGAGTTTAAGATGGATGGAAATATCGATCTAGGATAGGTATACATGTTGATGTGGGTTTTACTGATGCATATACATGATGGCATATGCAGCATCTATTCATATGCTCTAACCTTGAGTACCTATCTATTATAATAAACAAGTATGTTTTATAATTATTTTGATCTTGATATACTTGGATGATGGCATATGCAGCAGCTATATGTGGATTTTTTTAGCCCTGCCTTCATACGCTATTTATTTGCTTGGTACTGTTTCTTTTGTCGATGCTCACCCTGTTGTTTGGTGTTACTTCTGCAG

>prZmDUO1A

ACCACTAAACGGGAGGTAGTGATTAAGACTAACTTCAGCGGAGACCTTCAAATCATCCCACCCTATATCTAAAGAAGATTTACTCCTCTATATGCTCTAATGATGCCCTCTAAAACATCATCTAAGTATAGAGGACGCTAGTCCATCCTCTATATAGAGTCTCTCTCTGCTCTATCCTTTTTATACTTCAGATGACAGATTAAGTAAAATTTAAAAATATTTTTGAGGGCATAACAAATACATATGTACAAAAATCTAGAGCAAAACATGTCTATAATTTATGTATTTAGTGCATATAGAACAAGGTATAAAAGACATTGTTGGGGAAAATAATATACAAAGGAGGAAATCTTTTATAGAAGTTTGTAAAGAAATAATATAGAGAAAAACTATAGACAATATGGTTGGAAACGTCCAACCAGCACGGTGCAACTGTGAGCCTACGAGGCTTTCCCTGTTCATGTTTTTGAGCTAAGACCGATTTCAACCATTCCCCACATATATCCCTCTCAATCTATACTTTCTATTATACTCTCTCCGATTTGTTTTTATATGACGTTTCCGACAAGTTTGTAAGAATTAAGGAAACATTGATTTAACAATATTGCTCAGCCTCGGACCCACGGTGACGCATGCGAGTGAGTCAGAAGCAAAGCACTGGAGTTGAGAGGAGAGGGGCAGAGATGGAAGACCAGTGAAAATTTGTGAACTGGCGTCATATAAAGAAGACACTTTTATCTTGTGTATATAAACGAACAGAGGGAGTATTTTACTATATTACCTTAAAATATGCTCTCCTATATATAGGACATCTCTAACCACTCTCTATATAAATCCCCTATACAAAATTTATACTAACGTACTACATATTATTATTATCAGGTGAAACTTAAACAAGATTAATATCATAAAGAAATTGATGTAGTCAAAAAAAAATATTGGAAGAGAATAATGCTCACCTACATAAGAGGAGTTTCCTCTCTCCCCTCGTATGGGAGATCTTAGAGCATCTCCAAGAGCTCTCCATAAAACGACTCCTCAAAATCAGTTTTAAGGGACATCTAAATAATAAGTTGGGGTAGATTTTAATCCTTTCTCCAACAGATCCCTTAAAGCGACAGTTTGTTTCTGGGGAGCCCAAAAAACCCCTCATTTGTAGCTACAAATGAGGAAGTTTTAAGGGCTATGAAAAAGTTGGGAGCGCTTTAGGGGAACTGTTGGAGACATGTTTTTGTGTTTTTCCCGAAAAAATAGATTTAGGGGAGACTTCTGAGGAGCTTTTGGAGATGCTCTTAGAGAGCTCTTGGAGGGGAAAATCTTATCAAAGCCTCTCTTAAGTTATCTCAACAACTTACTCACTATTATCTCTTATTTTTATATCACACTTTGTAAATAATGTATTTTACAGTGCAAAGTGGTATTTTACACGGTTATATACACATATGAAGATTGTTGGAGACTGAATAAGCATGTGTCGTCATGAGAACGGAGAGGAGTAATAAAATCACTGATGTACCTGTATTGTACGTATCAAAAGTTTATTTTAATTCCCCAATAGTATTTTCACCTTGTTTCCACATAGCACGTACTCCGAAGTTACTTTGCATCCTTTGTTTGATTTATTTTCCGGTGAAAAATGCAAACAAGACGAAATCGACTTTTACCGGATACATAGGCGGTTGGAACTTGTAAGGGTAGCAATTCGTAAGTTCTTGATCGGACGGCAATAGAACGCCTAGTTTTTTTTTTGCAAGGTTAAAAAATGTTTTCCCGCACCACTTGTCGGAGCATAGACATGGAGTATCTTGGCCCGCGGAAATCGAAGGTCGTGGGGGTTGGGAGGACTAGCGGTTACCGTCCGCGTTCGTGCGCCCGCTCCATCTCTGGCAGCGCCCCCTGGAAATCAAAGCCGTCCGTCGGATCCCCACCGGACGTACTCCCCACAGCGCCACGTGCGGCACTCGCTCGCCCCTGCCCGGGCCGCATAAGGAGCACGACGACGCATCGCTCTCCCGGAACACTTGTCCTGCGAGATCGCACAGCGTCGAGACATTGGCTCGACCACCTGGCGGCGCCCGCCGGCGGGGCGGAGGCAGCGGCAGGAGGGACGCGGCGGGCGGGTGCGAGGCGGTGCGGAAGGGGCACTGGACAGCGGAGGAGGACGGTGTGCTGCTGGAGCACGTGCGCGTGCACGGCCCCCGGGACTGGAGCTTCATTCGATCCAAAGGATTGCTGCCGCGCACCGGCAAGTCCTGCCGCCTCCGCTGGGTGAACAAGCTCAGGCCAGACCTCAAGACGTGCGCGCCAACCCTCCCTTCTGGTTCACTTCTCAGTGCACTGTGGATCCTTAACTGCATTCGCATTGTTGTAACTGATGCGACTCTAGTTGTTAGATTAGAATTTCGTTTTTCGCTTGGGTTTAGGCTGCTGCACTGGTCCCGACGCTTGGTTTGTTGTGTTCTGCATTGTGTGCACGGTTCATTACAAATTCACCATGGTGATCGCCTTGCAACAGCGGCTG

>prZmIPI

ATCAACATTTGCTAATGTTTGGCTCGCAAAATCTTGGCAGCATTGACCGGACTTTATAAGTGTTGGCCAGATTTAACTAAGAATCTAATTATTACTAGTCTGAGGGCCAGTTTGGTAATCCCATTTTTTCGAGAGATTTCTATTTTCCGAAAAAATTACTTCATTTTCCCTGAGAAAATGGAGTTCCCAAACTAACCCTAAACAATATTTCATTTTTCTTCTCAATGTTAATTCAACTCTCATACATGTGTACCTAGCTCATGGTTTTCCGACGGTGACTACCCTGAAATATAACGAGGTTTGAGTTATGCTACAAATGATACTAACCTCAGTTTGACTAAGTTTTCCAGCAGCAACATCATCAGTAATAATAATAATAGTAATAATAATAATAATAATAATATGTGGCAATAAATAAGTATTTTTAAATATCTTATAAAATGGCATATATATACTTACAGATTGTAAACATTGTAATCAGTAATACGATTATCGATAAAGTTATTCAAATCTAGAATAGTTTGAATTAGGACGAGCTAAAATTCGTCATATTTTAGGTAAGTAGAGAATAAACATCTTCAGCATGATATTTGGATGTCAAAAAACTAGCACTAAACAAGATGTGGTGGTTTAACTTCTTGCCCTCTTTAGACAATATATTCACCTTCACATCAAAATTGCCTAATTTATAGATTGCCAAGGGTCTATATGTATTTAGACCATGTTTGGTGAAGTTCCGACACATCAAAACTGGTACTAAACTCGAGCTATTTTTTATCTCCCTCTCAAAATAAATTAGAAACATGTAAAACCACAATTTTTTTAAATTTACTGGCTTTGTCTCCTCCGTACACTGTAGCAAAGAGGTGGAGAAAAAATAAAACCATGCCAAATAGCCCTCCACTGTTTCCCTAGATATTAAATGGTAAATAATTGGTTACTTTTAGTTCAGTGGTCCGTGCAAAATATACATACATGATTTCCATAGTATAACACTATAGCATTAAGCAATGCTTAAATAGACTAACATTTCCATGATCACACTTGAGTATTTTACCATAAACTATGAAATGTTACTAGGAAGGATGTATTAGATGAAGTTTTCTTGAAGAAAGAGCATATGTATATAAGTTCTTAAAAATTATCCACCAATGATGGATAGACACGGAAAAGAAAAGTTATCATAAAAGTAATGTCAGAGAAGGGGACTAATTTTGTCGCTTCCCGAACCGACACTACGTGTAACAATTAAAAGCGAACGTATTTTTAAAAATGACTAACCCTTAAATTTGAGTCGTTGATCTATATCAACATTTCATCCAACCGCTCTCTTTATTTCTTCTTCCTCCCGTCGTCTTCTTCGTCGCGCCAACAGTGCTATAGTTCAATAGTACACCCTCGTCGCGCTAGCTCACTAGAGCTAGCGCCCTCCACTCCACCTAACCCTTCTAATGGTCGTCCTCTGATTGTTGTAGTCCACAATCCCCTCCTCTAATATCATCACTCTATTTGTAGCCTTAGCCTAAGATCGATTTTATAGTGTTTTGATGTTCTTGTTCTCCTGAAATCATCATGTCAATCGAAAAGGGAAGTGAATATGGTAATAAGTGAAAGAGAAACTCATATTTGATGGACCAAATATGATTTGGCAATTCCTGTTCTACTGTGTGTCCGGATCCAAATTTAGTCCGGCACATGTTTTGGCCGCGGGAGGACGAGGAAGGAAATGGTCTCAGAAAACCGGTGGCTGATGCGGACCGGAGAATCTCCGCCCGCAGGCAGGCCCACAGACATTTGGGGCCCCGCCACGCCGTCCGCCAGCCGCCAGCCGCCAGCCGCCAGCCAATCCCTTCCTGTTTCTATTTATTCATCCGCCCGGGCCCCTCCGCACGCCTGCACACATTCCGCGCACACCCCGGCAGCCGCAAACGCCTTCGCCGTCGCGTCCCGCTCCTCCGCCCGCCCGACGCGACCCCTAGGACCTGGAGAGAGAGGTCGGCCTGGCTGCCGCAGTGGTCGACGACGCTGGTCTGGACGCCGTCCAGAAGCGCCTCCTGTTCGAAGACGAGTGAGCCCTCTTTCCTTCCCCGACTCCTTCTCCTTCCTCCAGCTAATTTTCGCTCCTCCTCCACGATCTGCTTGTCCCCCACCTGTTCCGGTTACGCGCATCGATATTTGCTCGATTTTTCTGGCGCCTGGGGGCGGGGCTTTCGTTCGCGGTTGCACAGCTGCCTGCTCCGCCGGATCTCGCCGCGATCGGAACGCGGGCACGGTTTGGGTCACGGAGGCTGTTCAGTATGTTTGACTTTGTGCCAGTGATATACTGTACCACGTTTTAGTCTCACCGTCGCACTTGTTTGATGCGCTGTTTCGTCCTATACTCGTGCTGTGGGGTTAGCCGGACAGGACTGTCCTGCGCCAAAATTCCGGGCATGCCGTGCCTGCCCAGTATGCACTAGGCTCAGTAGGTCGATGTAAGGAGGTAGGTACTATAACTGAACTGTTTTTGTTTTTGCATCTGGAATTCAGGTTCAGTAAGTTTTTTTTAAATGATCTGGTTATGGATGTCCTTTTTGTTTAATTGACGACGGGTAGATGAGTAATTCTTTATGAATCATTTAAGTTGTGGAGTGCATAGTTGATGTGAATAGTGTTCAGTAAATCCTGGACGGTGAGTACTCTCCAAAGTTAGGGCATTGCTATACTTACAAAGTGGCTCTTTTAGGAAGTTATTATAGTACAAAATTTTAAGTGGATTGTGGAGATCCATGTGGGTGAACCTGGATCGAAGAGAAACCTGTAGAATTTGTGGATCAGTTCCTCACGTTTTTCTTATGATATAGTACATGATCTTTTCGCCATATTCAAGTGATTCACTGACAATTCTTCTTGACAATGCTTGCTTGATTTTTTTTCCCACAGCTGCATTTTGGTGGA

>prZmRZDP

AAAGCCTCCAAGCGAGTACCAACACTCCCTTTGAGGCTCGGGGGCTACTGTCGGGGACCATAATTAGGGGTACCCCCAAGACTCCTAAACTCGGCTGGTAACCACCATCAGCACAAAGCTGCAAAGGCCTGATGAGCGCAATTCAGGTCAAGGCTCCATCCACTCAAGGGACACGATCTCGCCTCGCCCGAGCCCAGCCTCGGGCAGGAACAGTAGACCAAGGCAGATTCACGCCTCGCCCGAGGGCATCCTCAAGTAACGGGCGCACCTTCGACTCGCCCGAGGCCCAGCTCGGGCAGGCTTCGCAGTGAAGCAACCTTGGCCAGATCGCCGCGCCAACCGACCGCATCGCAGGAGCATTCAATGCAAGGATCGCCTGACACCTTATCATGACGCGCGCTCCTCAGTCGACAAGGCCGAAGTGACCGCAGTCACTTCGCCCCTCCACTGACTGACCTGACAGGAAAACAGCGTCGCCTACACTGCTCCGACTGTTGTGCCACCCGCCAGGGTGAGGCTGACAGCCGCCGAGTCCAACCTCAGGCGCCATAGGAAGCTCCGCCTCGCCCGACCCTAGGGCTCGGACTCCACCTCGACCTTAGAAGACGGTCTCCGCCTCGCCCGACCCCAGGGCTCGGACTCCACCTCGACCTCGGAAGACGGTCTCCGCCTCGCCCGACCCCAGGGCTCGGACTCCACCTCGACCTCGGAAGACGGTCTCCGCCTCGCCCGACCCCAGGGCTCGGACTCAGCCTCGACCTCGGAGGAGTCACCGCCTCGCCGGACCTTGGGCTCGGACCGACCACGTTACAGGGGGGCCCATCATTACCCTACCCCTAGCTAGCTCAGGCTACGGGGAACAAGACCGGCGTCCCATATGGCTCACCCCGGTAAACAGGTAATGATAGCACCCCGCGTGCTCCATGACGACGGAGGTTCTCAGCCCCTTACGGAAGCAAGGAGACGTCAGCAAGGTCCCGACAGCCCCGACAGCTGTGCTTCTACAGGGCTCAAGTGCTCCTCCGACGGCCACGACGCCACATGCACAGGGCACTAACACCTCTCCGACAGCCACGTCGGCATGTACATAGGACTCTGACTCCTCTCTGCCGGACACGTTAGCACACTGCTATACCCACCATTGTACACCTGGGCCCTCTCCTTACGTCTATAAAAGGAAGGTCCCGGGCCCTCGTACGAGAAGGTGGCCGCGCGGGAGAACGGGCTGACGGGCAGGCTCTCTCTCCCTCGCGAACGCTTGTAACCCCCTACTGCAAGCGCATCTGTCTTGGACGCAGGACAACACGAGCCGCGGTTCCCCTTATTGTTCCCCCTTGTGTTCCGTCTCGCGCCGACCCATCTGGGCTGGGACACGCAACGACAATTTACTTGTCGGTCCAGAGACCCCCGAGGTCGAAACGCCAACTGTAGTGAAAGTTGAATTGACTTCTCTAAAATTTATTTCACAAGGTCTATCTATGCAATAAATGGAAACCGTAGCAACACACGGTCACGTACCTAGTTACACATGAATTAGATGAGATTATAAAAAATCAAGAAAAAAATTGACTTGAGATTTAAAACCACTCAATCTCAATCAATCCACATGAACCGATCAAGCCAAAATAAAGAGTGAGTGAGTTGTGTATAGAAAATATAGTTGAGTTGGGTTCGAATTCCTAATCCGTCCGTTGTACATCGCTAAATGCGAGCATCAAAACCAGCATCAGTTTTCCAAATTCCCACTCCGGATACCGAAACGACGTTCTGGTTCGCAAATGAAGCTTCAGTCAGTACGTGCGCCTATTAAATCGGCCATTTCTCACCCCCACCGAACGGACGGAACGGAACCCCTAGTGCGGTTAGCCAGGATTGAAGACAGAACAAGTTACATAAATGAGTGAACCATTCCACCTCAGAATTATTTTGGCCGCCGGTTCGAGATGAAACGAACGGGCCCTTAGCCTTCTCGCCTGCTTCTCATCTCAAACGCAAACGGA

>prZmTCXC2

TAGGAGGGGTTGAACAGTAAACTCCATACTCCACAAACCAAGTTTAAAAAATAGATTTTGAAGGGGAGATTTTGTGGGCTAATGCAAACATCGTTACATTTTGCGAATCCCTCTTAATAGTACGATGAATCCTATGACTAAAAAATGTAAAGTTTGAAGCTTTGACCGCCTGGAACCACAAGAGCTAATCAAAAAAAATTTGGATAATTTGCGTGATTTTCTGGTTCCTTATGCTTCAATTTTTAATTCCACATGATCTCCTGTACTTCTTTGAATCTAGGATAAGTGAGTACAACATGAAGCACACTTGTTAGATTTTGTTTTGTCATTTTATCACCAAAACCCTCAAGAGGTTGATTGCACTCACAATGCTCCAGTTTATAGAAACTAGATTACCAGTTTCTTAGAAACCAAGAAGCTACCCTCTCCTAGCTAAAACTACTTTTGTCCATTTAATTACATTATGTCCTTGTTGATTTCATGAAATTTACATCTATTGCCACCACTTTTTAAAATAGAGAAACGGTAGGCTAGTCATTATGTATCCAAAAAACTAGAAACTAGTTTCTTAGAAAATAGGTTACAAACATCATCAATCAGTCTTTTTTCATAAACTAGTTTCTAGAAATTGGTTTCTTAAAAACTAAGTTGCTTCCAAACATGATCTTAGTCCATGATTCATTCATGTGATACTACAGTAGAAATTTGCTAATTATGGATTAATTAGGCTTACTACATTTGTCTCATGTTTAGTCTTTGTCTATATAATTAGTTTTGTAAGAGCATCTTCAACAATGTGCCCTATAAAAATGCCCTAAAAATTTAAAATAAATATATTTTATAGAATTCAGGGCACCAACAAAAAAGGCTCCAACAGTAAAGTCCTAAATTAAGTGATTAATCGAAATATGTTAATATACCATGTTCGTTGCAAAGGAAAGTTTTGTAAAGCGTTCGTTCGTTTAGATCTAGATATGAGGCAGTCGAATAAGATGTTATATTTGTGGCACTTTAAGAGTGATCTATATTTTTTGACAGTTTTTTTGTAGCTTGAGCTCTATAATTTAATTTGGTACTAAACCACTGTTGGAGATACTCTAAACATATATCTAACAAGATCTGCTATATAGTTATGTACCCTAAATTTAGCGTCCACAACGTCCTCTACCGGCTCCAACAATTTACTGCAAACAACCGCTAAAATTTTTAGAGTGCATCCTCTAACCGATAAATGTGGAGGGCGTCATTCGTCCGCTGGACTAGTTTGAAAACTTAAATCTCCTTCAAAATTAAATGAGATTAAAGAGGAAATTAGTTTATTTTCACATTAATCCCCTATAATCTTGAAGGAGATTTGAGGTTCTCAAAGTAGCTCTAAATGTGCATGGCGAGAAAATTCGTGACAGGAGTATGGCGCAGGAGTGTGGGTCCACATGCTTCGGCCCACCGCCCACTGGACGGACTTCGAAACGTTTACCTAGTTTGGAGAAGTATTGATACAGATGATGAGATTTATAAAACACGTTGATATAGAGAAAGATATCCTTTAGAAGTTACCGTAAAGAACAGAAATATTTTTTTTTTAATTTGGAGAAGAATATTGATACAGAGGCCTAGATTTATAAGAAGTTGATATAGAGAAAGATATCCTTTAGAAGGTAATGTAAATAACAGAAACATTTCTTTCGAGCTAATTTGAGAACTATATTTTCTAAGAGATAAAATTTAGGAGACAACATAATAGACTTTATATTTGGTACTCGCAGTTACATCTCATATGGTACCGATTAAACCAACCAAACGCACCCTAAAAAATCTCGGTAGATAATTGTTGGAAATAGTAGGGTAGCCTATAAAGCGTAGAGATTTGAATCCAGCCTGAAGCATAGCCGCGTTGCCGTGCGTTAGAGGCGGCGGTGAGTAGGCAAGCGATACTAGCTAGGAGAGGAGACTGCAGGCACCGGCGGCGCGGGTGATCTCTCACGGCATCGCCGGAGCAGCTGGACGCTTCAGATAGCCCCCCATCCGGGCGACCGTCATCCTCCGCCGTCGCGCTCTACGGGGTTCGTTCCTACTCACACGCCCCCCGCCGGGGTGTGCCGCTTTTGTCTCAAGTGCTAAAAGGATTTGGGATCATAATTATGTATCCACGGGTTACGGGTGGGCTGACGATTGACATATGCAAGCCGTTTTTTTTTTTTTTTGTTAGCTCGTCTTCCCGGGTTACTTCATGTTGTCTGTAATGGCCAATTTCGCTGGGTTCATCGGTTCGAGTTTTGGGCTTCGTCTTTATGGGGGAGGTTAGGGTTTAGGATGAACCGCGATTGATGCATGCATTGTCATGTAAACGGTTTGCATGACTTGATGACTGCATAGAATTTTTTCCATGCTTCTGCAGTCTCCGGTCTCAGAACTAGTGTTTTCCCTTCCACTGATGTATGTGGTCATGTGGGACATTGCCTTGCATTTTGGTGGTCCACCGCGCGAATCAAAGCATTTCTCACAGTTTTCAGTTTTTGAAACTTTCAGTGCTCCTACGAATTATTTATAGGGTTCTGGCGGCACAATCGGAAGTGACTTAGTTGTGTTTCAGGAGCCAGGAGGGGATGTTTCGGTTAATTGACTCTGCAAATTGTTTGGAAATGCAGCTCTTTCTATTGAAACTTAAGGTTTAGATCCGTGTCGTGCAGCTGTTGCTCTTCTTCTCCTATCTGTGCATTGACTGCTGTCTAGTTCGTCAGCGTATGAATTTTCATATTGAAACGGATTATGACCGCTATCTCCTACAAATGCAAATCATGTGAGTAAATAGGTTTTATGTGGGTGCTTTATGTTGAAAGTGCCTGCTTTAGATCTACTAGCAATATTAGGCCCTCGCTTGCACTGCTTGTAGAGTCCGGTCTACCCCAACCAATATTATCAAATATGACTGTGTGTCGTCGTAATACACAAGCATTGCACGTACTAGTAAGATTGTTATTTCATGGTTAAGTTTTTTGAGTCTTGAGTTTCTAAGGTTTTACATTTACAGGACTCTCCTATCTTC

>prZmVSP

GGTCAGCGTGGACGCTGAATGGGCCGGCCCACGCGCGTGGGTGGAGATAGGGTGAGCCGAAGCAGGGGCTTCAGCCCAACTGGTGGTGTATCTTTTCTTTATTTTATTTTTTTCTATTTTCTTGTTTTTATTTTCTACCACAAAGTTGCCTTCAATCCCATTAATAATGTGACCCAAAATCCACATCAATGCAACAATAGAAACAAAAACCAGCATGGTGCATATCCTATTGATAGTTCATCGTTCAAGTTAATCACTTTTAAACATGAATACTTTTATGCTCAAATAGGAATCCATATCACACAATTGTATTATTTTTTTATCTATACTTTGGGTATTACAAAAGATAAGACTCAAAACATGTCACATTTGATTGTAGTGCCATTTGAGACATTTTTGCTCTTCTACGTGTATATAAATGGACACGTGCAAAAGATATGAGGTTACGGAAGAGCAACAAAATGGTATTAAAACCCAAGTGTGATATATTTTAAATCCTATTTTTAGGAAGTCAATATTACAAATACCAAAAGCATGAAAAACGTCGTTAGGGGTGAAAGTAGGTATCCAAATTGTTTGAACAAACTAGCATATAGCTCGTGCTAACGCTACAATCCTGTGAAGGGGTGGGGAGGGGTCGACGACGAAGGCAGCGACAAGAGGGGAGGTGTTGATACGCGGCGTTGACGCGAGTGAAGGGGGGAACACGGTGGGGACGTGGGAGAGAGGGGGACGTTGGGGCAGCGAGGTCGCAGGGAGGGGATGAAGGATGATCCAAATATCGTCCATTGCATTTGAGTAAGCAAGTGATGATTATGCAGTGATCTAAATCGTTGGTTTTGATGATGTGTTAAGTCTGGATATAATTTGTTTGGTGGATTTAATGGAGGTTATAAGTGTGGGGTGATTTTGTTGGATGAGTTTTGTAAAGTTTAAACTGGTGGATTATATAGTTATATAGATTTAATATTTTAAAATAGATATGCATGAAATTTGATGTTAATCTTTTCTTATGTTATCAAGCACATTATATTTTTTTACAAATTGTTTTATACATTATTTGCGCCCTACAACAAAAAAGTGTAAAACACTAGTTTTACGGTTCCATGGCCAACGTGCTCTCCCACATTTAAATAGTAAAAATGATAAAATTATGTACGAGGATTCGAACCATGATTGTTGGCTGTAAACCCACAATCTGGGTACGATTTAAGTTTATTCATAAAACGCAAAACAAGTATATCTTTTTGTTATATATTCTATATAAGCATAAATAAAAAACCATAGCAACATATATGTACTTTGCTTTTTAAGAAAATATATGATAAAATTTAAGTTTAGTTTTTTATAATTCTTCATAAGTTTTAATACTAAAAACAATAATATAATTTTTTATAGCAGGTTCTATATCATATTTATACATAATCAAATAAAAAATAAAAAATCGATATTAGAATATCCGTTTTCGTCCCTCCACAACGTTTAACCCAAAGTAACCGTCCCGTGTTTCCGTTTGCTTCGCCTGGTCACCTTCCGTGGTGCCTGTGGCTGTGTACGCGGTCGTCTCCATCCGACCCATCAATCATCCAAAAACTAAACCAAATCTTCCCCATCGATCCCAACTTCCAGTCGATCTCCTCGCTCGGCTGTTGCCTCCCAAAGCCGTGCCTTACAACGAGATTCCTCCCCTCCTCTCTCCAATAACCCTAGCCTCCTCCGCCGCCGGCGACGAATCCGGTAAGCGTCCTCGCTTCTGCTAGATCCACCGCCTCCAGATCGGTGCGGCCCGCGACTAGGGAGAGGGTCGGTCGCGATCCGCCGTTCCTCTGCCTTAGGTCATCGCGGTCGGTTAGTCGGCACCCGTGGACAAGCTCTCCTTTTCCACTCCAGTTATTTTTGGGTTGTGCTTGCAATTGCTACTTGATGAGTTTCTCGAATTAGCTTGTGAATGCCGTTGTTTTGCCTCGCAGGCGTTTTGAGTTTCTGTTGCCAGCGTAGCCCTGTCCCTGTTCAATAGGATCTTCGGGAAGCCCAAGGAGCAGGCCAACTCCACTGCCTTGGCCACTCTGGACAAGTTAACTGAGGTGACCTTTCTGTCTTCTTTATTTATTTGCTGTCGTATGATTACCTTGGCTTATACTTACATCAAGGTGTTTTTTTGGGCACGTATTGATGTTTGCTTATTTTGGCTGCCTAAATATAATTCCCACCCTTCTACTTAAGTGTCTTGGAACTCTCACATTTTGTTAACTTCATCTCAACTTATGGGATGCTTAAGTTCTTCTCATGACATTTGTATGTTCCATCTATTTATGATCAGTTAGTACAATAGGCTGACCATTCTTTGATTTGCTCATGAAGTTCTAACGTTTTACCTATTAGGAAGGGACCAGTATCGTTAACTCTTTACATATGTTTTGCCAACTAGGAAACTATGCTCTTGTTATAGCTGGCAATTTGGCACATGTAATGCCTATGCTAGTAAACGAGAAAGTTCTAAAAGCTCACATTATTTATATTTAGACTCTTGATCTGCTG

>prZmVSP'

GGTCAGCGTGGACGCTGAATGGGCCGGCCCACGCGCGTGGGTGGAGATAGGGTGAGCCGAAGCAGGGGCTTCAGCCCAACTGGTGGTGTATCTTTTCTTTATTTTATTTTTTTCTATTTTCTTGTTTTTATTTTCTACCACAAAGTTGCCTTCAATCCCATTAATAATGTGACCCAAAATCCACATCAATGCAACAATAGAAACAAAAACCAGCATGGTGCATATCCTATTGATAGTTCATCGTTCAAGTTAATCACTTTTAAACATGAATACTTTTATGCTCAAATAGGAATCCATATCACACAATTGTATTATTTTTTTATCTATACTTTGGGTATTACAAAAGATAAGACTCAAAACATGTCACATTTGATTGTAGTGCCATTTGAGACATTTTTGCTCTTCTACGTGTATATAAATGGACACGTGCAAAAGATATGAGGTTACGGAAGAGCAACAAAATGGTATTAAAACCCAAGTGTGATATATTTTAAATCCTATTTTTAGGAAGTCAATATTACAAATACCAAAAGCATGAAAAACGTCGTTAGGGGTGAAAGTAGGTATCCAAATTGTTTGAACAAACTAGCATATAGCTCGTGCTAACGCTACAATCCTGTGAAGGGGTGGGGAGGGGTCGACGACGAAGGCAGCGACAAGAGGGGAGGTGTTGATACGCGGCGTTGACGCGAGTGAAGGGGGGAACACGGTGGGGACGTGGGAGAGAGGGGGACGTTGGGGCAGCGAGGTCGCAGGGAGGGGATGAAGGATGATCCAAATATCGTCCATTGCATTTGAGTAAGCAAGTGATGATTATGCAGTGATCTAAATCGTTGGTTTTGATGATGTGTTAAGTCTGGATATAATTTGTTTGGTGGATTTAATGGAGGTTATAAGTGTGGGGTGATTTTGTTGGATGAGTTTTGTAAAGTTTAAACTGGTGGATTATATAGTTATATAGATTTAATATTTTAAAATAGATATGCATGAAATTTGATGTTAATCTTTTCTTATGTTATCAAGCACATTATATTTTTTTACAAATTGTTTTATACATTATTTGCGCCCTACAACAAAAAAGTGTAAAACACTAGTTTTACGGTTCCATGGCCAACGTGCTCTCCCACATTTAAATAGTAAAAATGATAAAATTATGTACGAGGATTCGAACCATGATTGTTGGCTGTAAACCCACAATCTGGGTACGATTTAAGTTTATTCATAAAACGCAAAACAAGTATATCTTTTTGTTATATATTCTATATAAGCATAAATAAAAAACCATAGCAACATATATGTACTTTGCTTTTTAAGAAAATATATGATAAAATTTAAGTTTAGTTTTTTATAATTCTTCATAAGTTTTAATACTAAAAACAATAATATAATTTTTTATAGCAGGTTCTATATCATATTTATACATAATCAAATAAAAAATAAAAAATCTATATAAGAATATCCGTTTTCGTCCCTCCACAACGTTTAACCCAAAGTAACCGTCCCGTGTTTCCGTTTGCTTCGCCTGGTCACCTTCCGTGGTGCCTGTGGCTGTGTACGCGGTCGTCTCCATCCGACCCATCAATCATCCAAAAACTAAACCAAATCTTCCCCATCGATCCCAACTTCCAGTCGATCTCCTCGCTCGGCTGTTGCCTCCCAAAGCCGTGCCTTACAACGAGATTCCTCCCCTCCTCTCTCCAATAACCCTAGCCTCCTCCGCCGCCGGCGACGAATCCGGTAAGCGTCCTCGCTTCTGCTAGATCCACCGCCTCCAGATCGGTGCGGCCCGCGACTAGGGAGAGGGTCGGTCGCGATCCGCCGTTCCTCTGCCTTAGGTCATCGCGGTCGGTTAGTCGGCACCCGTGGACAAGCTCTCCTTTTCCACTCCAGTTATTTTTGGGTTGTGCTTGCAATTGCTACTTGATGAGTTTCTCGAATTAGCTTGTGAATGCCGTTGTTTTGCCTCGCAGGCGTTTTGAGTTTCTGTTGCCAGCGTAGCCCTGTCCCTGTTCAATAGGATCTTCGGGAAGCCCAAGGAGCAGGCCAACTCCACTGCCTTGGCCACTCTGGACAAGTTAACTGAGGTGACCTTTCTGTCTTCTTTATTTATTTGCTGTCGTATGATTACCTTGGCTTATACTTACATCAAGGTGTTTTTTTGGGCACGTATTGATGTTTGCTTATTTTGGCTGCCTAAATATAATTCCCACCCTTCTACTTAAGTGTCTTGGAACTCTCACATTTTGTTAACTTCATCTCAACTTATGGGATGCTTAAGTTCTTCTCATGACATTTGTATGTTCCATCTATTTATGATCAGTTAGTACAATAGGCTGACCATTCTTTGATTTGCTCATGAAGTTCTAACGTTTTACCTATTAGGAAGGGACCAGTATCGTTAACTCTTTACATATGTTTTGCCAACTAGGAAACTATGCTCTTGTTATAGCTGGCAATTTGGCACATGTAATGCCTATGCTAGTAAACGAGAAAGTTCTAAAAGCTCACATTATTTATATTTAGACTCTTGATCTGCTG

>tNOS

GATCGTTCAAACATTTGGCAATAAAGTTTCTTAAGATTGAATCCTGTTGCCGGTCTTGCGATGATTATCATATAATTTCTGTTGAATTACGTTAAGCATGTAATAATTAACATGTAATGCATGACGTTATTTATGAGATGGGTTTTTATGATTAGAGTCCCGCAATTATACATTTAATACGCGATAGAAAACAAAATATAGCGCGCAAACTAGGATAAATTATCGCGCGCGGTGTCATCTATGTTACTAGATC

>tUbi1

GTCATGGGTCGTTTAAGCTGCCGATGTGCCTGCGTCGTCTGGTGCCCTCTCTCCATATGGAGGTTGTCAAAGTATCTGCTGTTCGTGTCATGAGTCGTGTCAGTGTTGGTTTAATAATGGACCGGTTGTGTTGTGTGTGCGTACTACCCAGAACTATGACAAATCATGAATAAGTTTGATGTTTGAAATTAAAGCCTGTGCTCATTATGTTCTGTCTTTCAGTTGTCTCCTAATATTTGCCTCCAGGTACTGGCTATCTACCGTTTCTTACTTAGGAGGTGTTTGAATGCACTAAAACTAATAGTTAGTGGCTAAAATTAGTTAAAACATCCAAACACCATAGCTAATAGTTGAACTATTAGCTATTTTTGGAAAATTAGTTAATAGTGAGGTAGTTATTTGTTAGCTAGCTAATTCAACTAACAATTTTTAGCCAACTAACAATTAGTTTCAGTGCATTCAAACACCCCCTTAATGTTAACGTGGTTCTATCTACCGTCTCCTAATATATGGTTGATTGTTCGGTTTGTTGCTATGCTATTGGGTTCTGATTGCTGCTAGTTCTTGCTGAATCCAGAAGTTCTCGTAGTATAGCTCAGATTCATATTATTTATTTGAGTGATAAGTGATCCAGGTTATTACTATGTTAGCTAGGTTTTTTTTACAAGGATAAATTATCTGTGATCATAATTCTTATGAAAGCTTTATGTTTCCTGGAGGCAGTGGCATGCAATGCATGACAGCAACTTGATCACACCAGCTGAGGTAGATACGGTAACAAGGTTCTTAAATCTGTTCACCAAATCATTGGAGAACACACATACACATTCTTGCCAGTCTTGGTTAGAGAAATTTCATGACAAAATGCCAAAGCTGTCTTGACTCTTCACTTTTGGCCATGAGTCGTGACTTAGTTTGGTTTAATGGACCGGTTCTCCTAGCTTGTTCTACTCAAAACTGTTGTTGATGCGAATAAGTTGTGATGGTTGATCTCTGGATTTTGTTTTGCTCTCAATAGTGGACGAGATTAGATAG

>tZmDUO1A

CTCTGGATATTTGAGGTTGCCCTTTCTTAACGATGAGTGCCGTAGCCCTTGGCCTTGGCCTTGCTTGACCATTCTTTTCCACGATATCTGTCGCCTGACCATTTCCACTCGATTCACCCGACGGTCAGAGCGACTGGACAGCGTGCCATGCCTTTTCCTGTCTCTCTACAGGAAAATATATAATGTATTTATTGTGATCTATAACAATATATTTCAGTCTTTTATATTACCGTATTATAGAAAAAGTGTAATTTATTCTTTTGCCAATAATTACTAACAGATTTGGACTAGTAGATGAAGATCTCGTGTGAAACTCTGAATAGAAGTTGTGAGTGTGTTTATTATGGCTCTAGAGTAAAACTTTAAACGAGAGTTGGTCGGGGGTATTCAAACTCTATCGAGTTTTGAGAACAGCCGTATAATTTTTTGAGTAACTCTTTTGTTGTTCTAAAAATCCTTAAAAAGCAACATAAATATAAAACTTGAAGGTATTTACCCTTCAATGTCACTGCTTATGTAACACACCAGGTGTTCGTCACGTGCTAAATAACAATTTCGAACTCAAGCAATTAAATTGGTGAGTATAATAAACCCTAAGTCACATTCATTTCTGTCTATTTCGATGATATCAAGAATCTTGTAAAGTTCGGAAATTATTTCGTCACACTAAATAATGAGTCCAAGTTCTGTTCGATCGTAAAGTGAATATACAAATTCGAATAATCAACTAGTCTCGTGATTTTGTTACAAACAAATCCAGTAGAACTCGACGACATAAGTTAGAGTAAAATAAAGTGAGCGGATAAATCTAATCGAGCCCGAACTAACTGAAGCCGATTTAATCACTATCTAAATTGGAACTTTGATCTGAAAACAAAAATATAATGTCATATTTAAAACGCGCTTGAAGTTCATAATCTAGATTGTGCGCGTTATCAAACTGAATTACGTACGAGGGTAATAATCGGTGTTCGGATAGTTAGCAGTTGGAAATATCTAA

>tZmIPI

GGGGCCGCCGGCCGGCCGGCTCCGATGACCTCACCACCTGTTGATGTTGCTGCTGCTGCTGCACTGCATGTTTATCAAAAGTTATCGCTCCTGCTCGCGGAAAGTGAGCTTGACTGTTGCCGGGGTGGAAGTGTCGTTTTGGACTGAAGATGAGTGCCGCGGAGGGGTTTGTTGTTTGTTTGTTTGTTTGTTCGGTGACCGAATCGCGAGTTGGACGCCTGTTTAATCCGTGCTTATACATCGTCTGAGTAAACAGCAATAAGAGGGACATCCGTAAGCTCTTTCCGTAACTGCTTTTGCGACTCGTCTGCTGCCACTGCAGTTCCATTTCTGCATATTTTTTTGCAGAAGATCCTTGTGCAGCCTAGGCTAGCGGCAACGTGACTGGGAGGTAGGGAAGGGAACGACGCTTGTGCTTGTGCATGTTGGGTTGCTTACTTGCTTCTTGAGCTCTTTGCTTGTGGTTGTGGCTGATTTATTTTTTTTTTTAAAAAATAACTTTAGGGGCTCGTTTATCTCAGTATAACATGGGGGTTTGGGTTTATTTGCTTGTGACTGAACTTCTGTTTATGAACTTAAGGGGCTGGCTTATCTGATTATTCTAACATGAGGTTCAATAGTATCACGAACCACAACATCCAGTTAGGCCTCGTTTGGATCATTAGGATTGAATTCATTCTAATATATTTATATACTATTGTAGACTATATGAGAGAATACTTATCTGTATTTCTGTTGTAGGAGAGCGGAAATACGATCTGAAGAGCGTTTACAAATATAAATATGATGTTGTGAGAGAGTAATCCGAAGAGTGTATTATAAGTTAAATAGTCTAAATGCAAGACCTTAGAAATGCTACTTAGATCATTAAACTAAAACTTGTTTAATGAAACTGTAAAGTGACTAGTATCCAAAATGAATCAACTCTGTCTAATTCCTTTATTGCTCTGTTATTTATACATGAGGGGATGCCTTGAGGACCCGGCGTATAGAGAAGACA

>tZmRZDP

GTTCAGTACTAAACAAATACCTGCGTAGTGTTGTTTTGTTTTTGTTCAATTTAGAAGTCCACTGCAATGCTCGAAATATTTAAGGTTCTTTTTAAAGAAGATGATGAGAATTAAGGTCCTCGTTTGTATGAGTTCTCTATTGTAGGTTCTTCATTGACTCAATCAAAAGTTTAACCAACCAAACTTAGTTAAAGCACTTCGTCGTAAACCAAAATCAAGGCAAAAATGAAGCCAAAGTCAGTTTTAGCTAGAAACTGAAACCACGCAAAAACATGATTTCTTATCATTATTAAGAAGCTCTAATCGTACTAGAGAGTAGAGACCTTAACTTATAAGGTCTAGAAATCTTGGTTTTATCGGATAGATTCAGTGTCGAAGACTCATATATAAACAGGCCTAGAAAAAGAATAGAAAAATACGAGCTTAAATTTAATAGATCAAGGCACATGTGCAGCTCGTTTTTGTTGAGATTTTTTTGAAGCAATGTAATTGTCTTGAATCATTTCCTTTGCATGGAAATATTCTTTTGGCCGAGAATTTGAGGATTTATGATTTTGTAGGTGTGGCTTTGAGTTTTGCTATGGATGTGGCAAGAAATGGACGACTTCTCATTCCCGTTGCACAACAGCCTGAGATTCGATGGATGCCATCTTGGACCCAAGCACAAGGCCAACATCATGGTGGTTTTATATATCAATTTTAAGTGTCATTTTTATTTCCTTTAGTTTCTAAGTATTTTTATTAATAACTTGTCAATCATACCGCTTCATATAGTCCCTCCGTTCGTTTTTGTCCTACGTATTTGCTCACTTCACGGAGACCAAGAAAATGGATGCAAACACGTAAGACTGCGTTAAAACCACAACGCTCGTTTGGGCTTCTCAAGCGCAAGCCCACCAAAACAAACTCTGTTGCTCCCTCCATTCATTTTTCTGTTTGGTCGCTAACGAGGCACGCCACTACAGGACTTGTCTTCTTTGCCAAGTGCCACAGACACTAG

>tZmTCXC2

ACAGAGTTCATCAGCATATGACAACTGAGGTACGCGTTTGCTGTTTTCCTTTCTTCTCTCTGATGTTTCTGTTTACAAAACTAAATTGATCATGCAGTTTCTTTTTTTTTCAGTCGACAGCTGATCCCTTCTGGTACCCCTGGACCCTGGTTTTGACAAAGAGTTGAAAGAACTTGTTTCAGCACCTCTACCATTTAGAAATATAGTTGCGCTATCTTGACAGGCCGATAACAATTCTGCTAAATGTGTTTGACCTGTCGCCGCCTAAGGTCAGTATGTACATATTTCCACACCTCGTGGGGAAAAACTACATACGTATCGAATAATGAATGTTTGAGTTTAGCACAGTAGTCAATTCAGTTACATAAGTTTGTGACCAAACATCACTGCTGTTATTTCCAATCATTTTCCTACCCCTATTAACTATAAGCAATGCTGGGTTGTCTTGGCTTTTTTGGTATCAGCGGTCCGTCATTTGCAACTAAATGAATGGATCTAGAACCCCTACGTTCATCGCTGAATATTTGAGCAGTAGCATTACCTCAAGAAAAAAATTAATAAACTTATTATGAGCTCATCCAAAGACGTTAATACATATTAC

>tZmVSP

TCAGGTTTCCAATGAAGAGGTGGAATGTGTATATGAATGGCGTTATGCTACCAAGATGAAAGTTGAGTCAGGATTTATTATATTTGCCATGTGTATAATCTTGGGGGATATTTGGCAAGCAGAAAAAAAGCTGCTCCAGGCAGTGTTTGTTTCAGTGATTTTGGGCTCTGTTTTCCACAAACAGTTTAATGTCGCCCGGTTAGCTCTAAGCTAACTATTCAGGTCACCAACGTGCATTGTTTGTCACAGTATTTTTCATTTACCCATATTGTCTGGAGCCATGTAAGAGTGTTGGAACGTATAAAGCCTCAGGGTACACATTTGATGTGCCGGTTTGCAGCTATTTCGACCTCAGTCAGCATGTTTCATCGTTGCAACTGAATTACCAGTATTGTTTTTGTACCGAGCAGTGTGTTTCGCCTGAGAATGACGGTCGAACATGTAGGCATGAACACTGAAAGCATTAGGCTCTAGTCTTGGTTTCTATGATTCATAACAATAAGATTATTGTAATTAATGTG
