# Supplementary material for: A Robust Framework for Maize Elite Line Genome Editing Through Enhanced HI‐Edit via LbCas12a Activity Optimization
Source: Plant Biotechnol J. 2026 Jul 4:10.1111/pbi.70715. Online ahead of print. doi: 10.1111/pbi.70715 (PMC13399594; doi:10.1111/pbi.70715)
Supplement: Supplementary file 1 — Figure S1: Technology concept and advantage of HI‐Edit, compared with conventional process. Figure S2: HI‐Edit promoter validation. Figure S3: Schematic T‐DNA vectors using selected promoters for HI‐Edit test. Figure S4: Zygote editing rate (ZER) on gene targets Wx1 (left), and Gl2 (right) were evaluated by event and tester. Figure S5: The zygote editing rate (ZER) under normal and heat‐treatment conditions. Figure S6: Optimized methods of heat treatment. Figure S7: The haploid induction rate (HIR) showed no significant difference (p > 0.05, n.s., not significant) between normal and heat conditions. Figure S8: Correlation between haploid editing rate (HER) and zygote editing rate (ZER). Table S1: The haploid inducer (SYN‐ INBD56) transformation with LbCas12aV Vectors. Table S2: Copy number analysis of T0 events using Taqman assay. Table S3: Molecular analysis on transgene presence in Putative Haploids (PH*). Table S4: The Haploid Induction Rate (HIR) % of inducer (SYN‐ INBD56) events transformed by LbCas12aV Vectors. Table S5: The haploid editing rate (HER) and zygote editing rate (ZER) of HI‐Edit vectors and events. Table S6: Genotype of target genes in edited haploids by NGS. Table S7: Genotype of target genes in edited F1 diploids. Table S8: The haploid editing rate (HER) under normal and heat treatment conditions. Table S9: The zygote editing rate (ZER) under normal and heat treatment conditions. Table S10: The haploid editing rate (HER) of UBA2 fusion and control vector (27680) under normal and heat treatment conditions. Table S11: The zygote editing rate (ZER) of UBA2 fusion and control vector under normal and heat treatment conditions. Table S12: The ZER assay of reciprocal crosses. Table S13: Quantification of Cas12aV protein in leaf samples from UBA2 fusion and control (CK) vectors. Table S14: HER for different events with different copy number and insertion sites in the genome. Table S15: Primers and probes of Taqman assays used for copy number of events, [file PBI-9999-0-s001.pdf]

Supporting Information

A robust framework for maize elite line genome editing through enhanced HI-Edit via LbCas12a activity optimization

Dawei Liang[1,2†*], Huanhuan Guo[2†], Juan Wei[2†], Fugui Zhu[2], Yuguo Zhang[2], Julie Green[3], Yun Ji[2], Huaibing Jin[2], Xiujuan Zhang[2], Huaping Gui[2], Hongmei Dan[2], Yubo Liu[2], Yu Zhang[2], Han Wang[2], Yutong Jiang[2], Lizhao Geng[2], Jian Lv[5], Wen Cai[2], Weibin Song[1], Timothy Kelliher[4], Qi Xie[2], Xi Chen[2,*], Rachel Egger[3,*]

*Corresponding authors: dawei.liang@syngenta.com, xi.chen@syngenta.com, Rachel.Egger@syngenta.com

Contents

Figure S1....... ……….Page 3

Figure S2....... ……….Page 4

Figure S3.... ....Page 5

Figure S4..... ………...Page 6

Figure S5.............. .....Page 7

Figure S6.............. .....Page 8

Figure S7.............. .....Page 9

Figure S8.............. .....Page 10

Table S1......................Page 11

Table S2......................Page 12

Table S3…………........Page 13

Table S4.......................Page 14

Table S5.......................Page 15

Table S6.......................Page 16

Table S7.......................Page 17

Table S8…………........Page 18

Table S9.......................Page 19

Table S10.....................Page 20

Table S11.....................Page 21

Table S12.....................Page 22

Table S13.....................Page 23

Table S14.....................Page 24

Table S15.....................Page 25

Table S16.....................Page 26

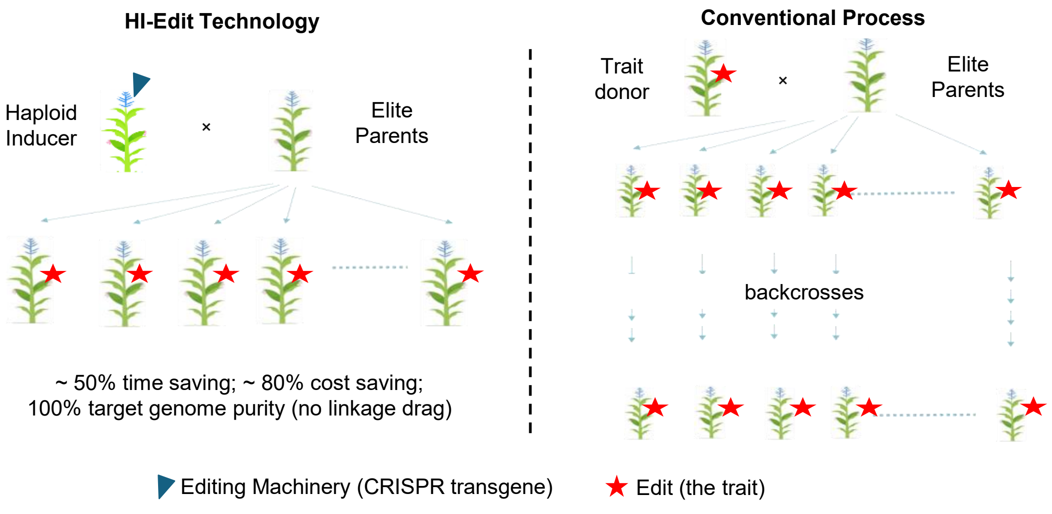

Figure S1. Technology concept and advantage of HI-Edit, compared with conventional process.

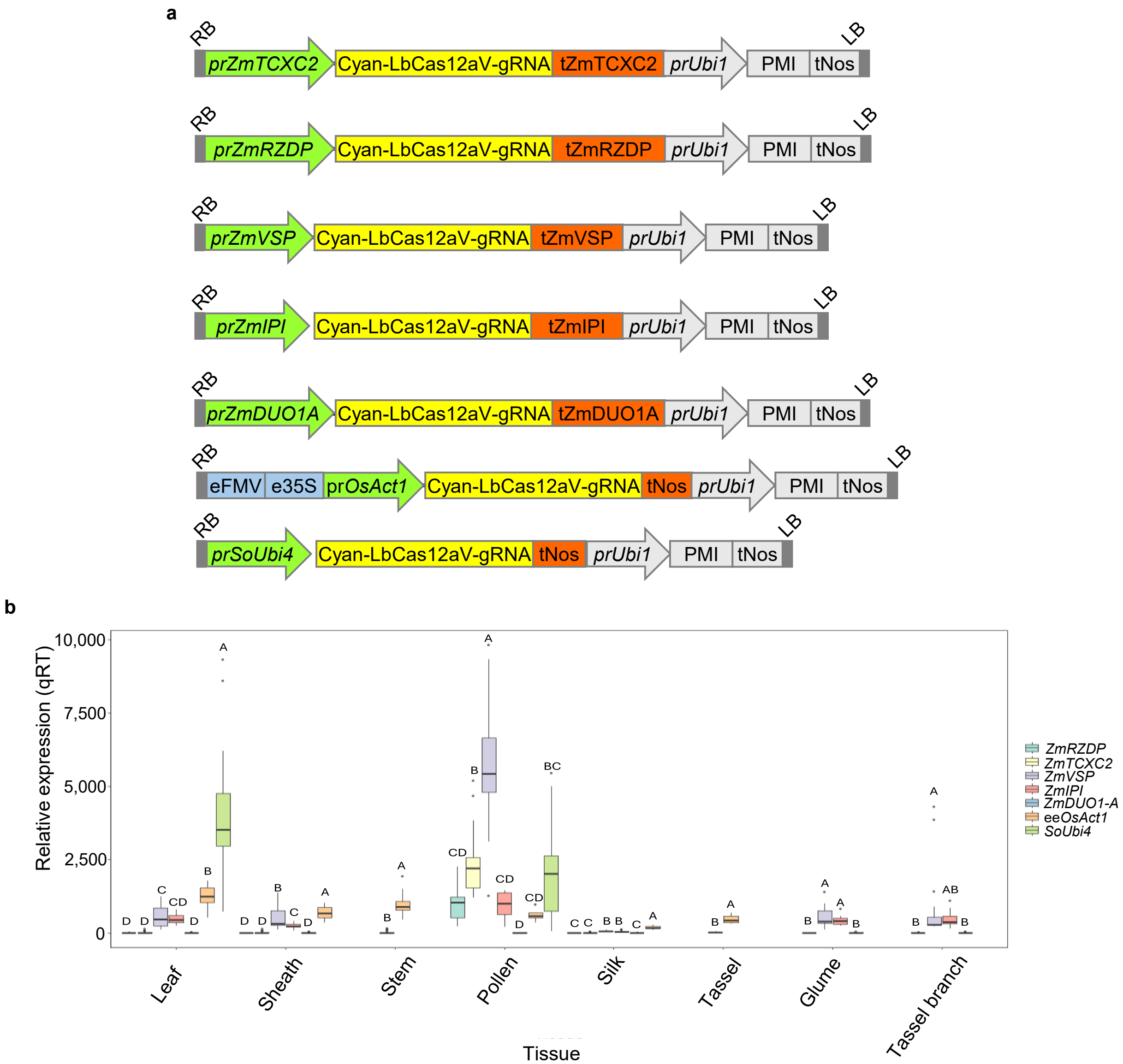

Figure S2. HI-Edit promoter validation.

(a) Binary vectors for HI-Edit promoter test. LbCas12aV was fused with cyan fluorescent protein (CFP), single transcript unit (STU) design was used for Cas and gRNA expression targeting *Gl2*. Cyan-LbCas12aV expression was driven by selected promoters and corresponding terminators. *prOsAct1* was combined with two enhancers (eFMV, enhancer from figwort mosaic virus; e35S, cauliflower mosaic virus 35S enhancer).

(b) LbCas12aV expression driven by different promoters. Leaf, pollen and other tissues were sampled for expression detection based on quantitative reverse-transcriptional real-time PCR (qRT-PCR), Different letters indicate significant differences (Fisher's LSD, P <0.05).

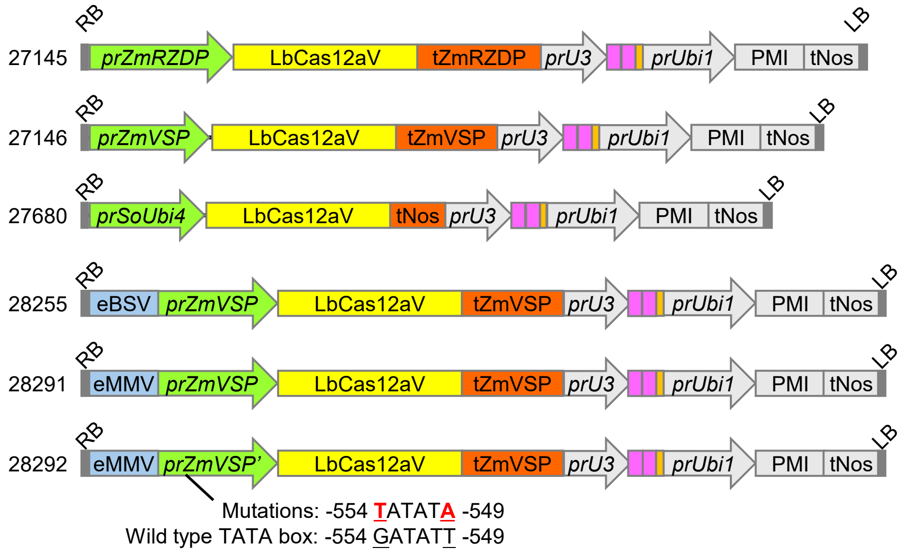

Figure S3. Schematic T-DNA vectors using selected promoters for HI-Edit test. LbCas12aV was driven by *prZmRZDP* (27145), pr*ZmVSP* (27146), *prSoUbi4* (27680), eBSV-pr*ZmVSP* (28255, eBSV- enhancer from banana streak virus), *eMMV-prZmVSP* (28291, eMMV- enhancer from mirabilis mosaic virus), and *eBSV-prZmVSP'* (28292, with TATA box mutations for higher expression). tNOS, NOS terminator; *prU3*, rice *U3* Pol III promoter; purple boxes indicate gRNAs targeting *Gl2* and *Wx1*; orange box indicates ployT; *prUbi*, maize ubiquitin promoter; RB/LB, T-DNA right/left boarder; *PMI* (phosphomannose isomerase) was used as plant selectable marker.

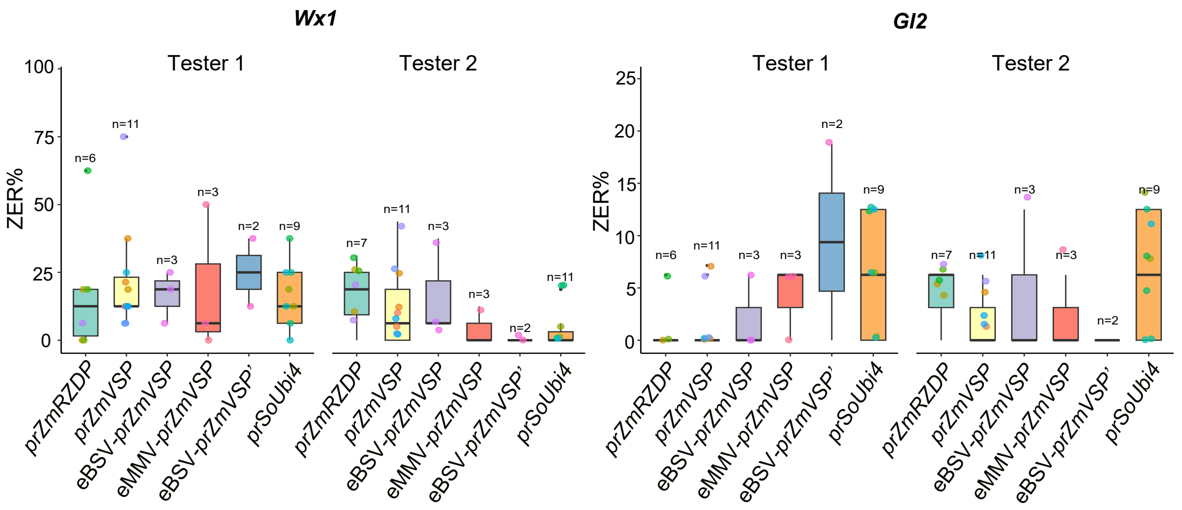

Figure S4. Zygote editing rate (ZER) on gene targets *Wx1* (left), and *Gl2* (right) were evaluated by event and tester. Events used for outcross were generated from vectors which LbCas12aV were driven by promoters of *ZmRZDP* (pr*ZmRZDP*), *ZmVSP* (pr*ZmVSP*), combining pr*ZmVSP* with viral enhancers (eBSV-pr*ZmVSP*, eMMV-pr*ZmVSP*), and TATA box mutations of *ZmVSP* (pr*ZmVSP'*), *SoUbi4* (*prSoUbi4*). Different color dots indicate different events. Sample sizes (n) are indicated above boxes. Boxplots show medians, quartiles and whiskers (1.5× IQR).

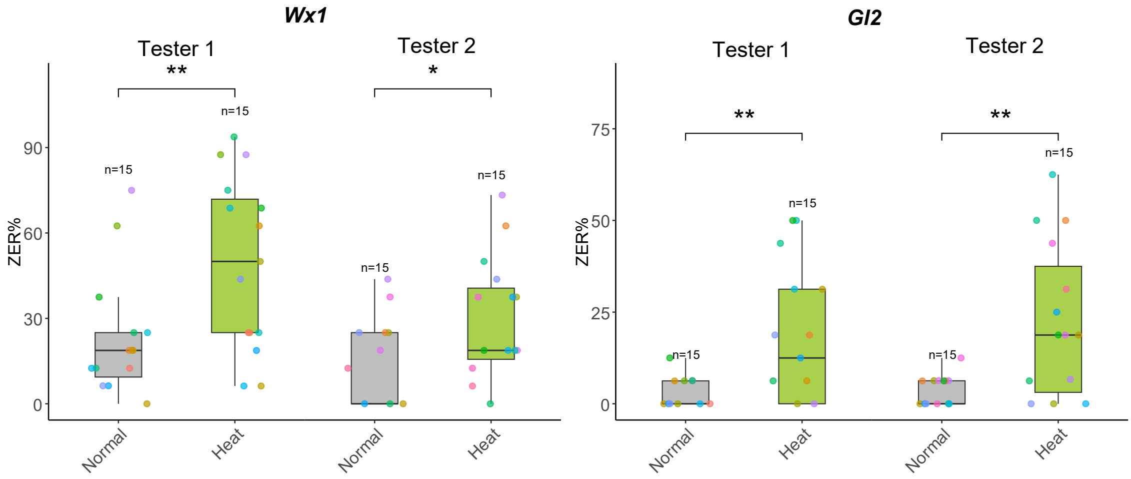

Figure S5. The zygote editing rate (ZER) under normal and heat-treatment conditions. Different color dots indicate different events. Sample sizes (n) are indicated above boxes. Boxplots show medians, quartiles and whiskers (1.5× IQR). Asterisks denote significant differences (Welch's *t*-test: ***$P < 0.001$, **$P < 0.01$, *$P < 0.05$).

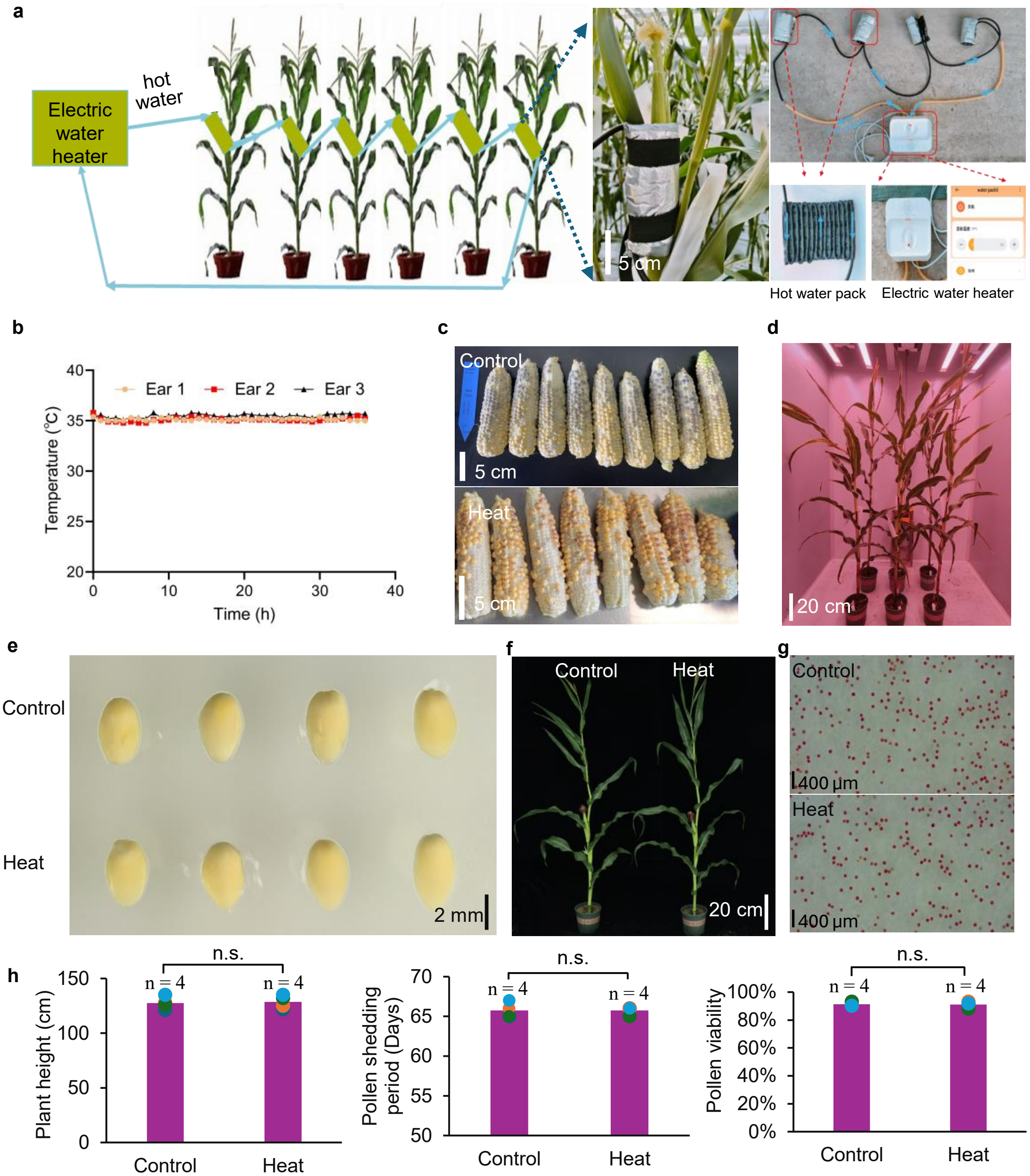

Figure S6. Optimized methods of heat treatment.

(a) Hot water pack system for heat treatment. The ears were wrapped with hot water pack, electric water heater was used for heating water and maintaining temperature.

(b) The temperature of the ears was stable and accurate, which was measured by inserting a senor into the husk.

(c) Ears after heat treatment (hot water pack).

(d) Heat treatment by putting plants after fertilization into a hot room.

(e) Morphology of haploid embryos recovered from heat-treated and control ears.

(f) Doubled haploid plants of tester1 derived from heat treatment and control embryos.

(g) Pollen viability from doubled haploid plants of tester1 derived from heat-treated and control embryos.

(h) Comparison of agronomic traits between doubled haploid plants of tester1 derived from heat-treated and control embryos. The table shows plant height, pollen shedding period, and pollen viability (n = 4 per group). Pollen viability was determined by TTC (2,3,5-triphenyltetrazolium chloride) staining. No statistically significant differences were detected between the two groups for any trait (Student's *t*-test, $P > 0.05$, n.s., not significant).

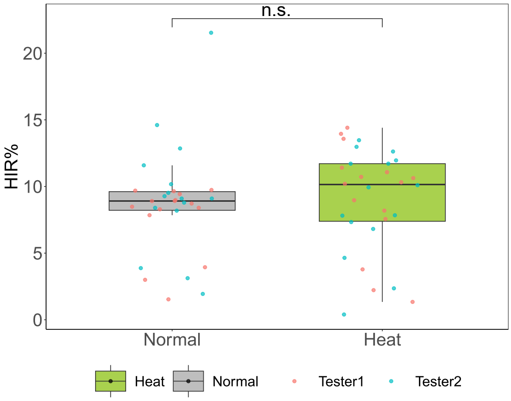

Figure S7. The haploid induction rate (HIR) showed no significant difference (*P* > 0.05, n.s., not significant.) between normal and heat conditions. LbCas12aV-homozygous events were used as male to outcross with Tester 1 and Tester 2 ears. F1 young embryos were isolated and haploids were identified. HIR was calculated accordingly. Boxplots show medians, quartiles and whiskers (1.5× IQR). The dots represent events, red and blue dots indicates outcrossing with Tester 1 and Tester 2, respectively.

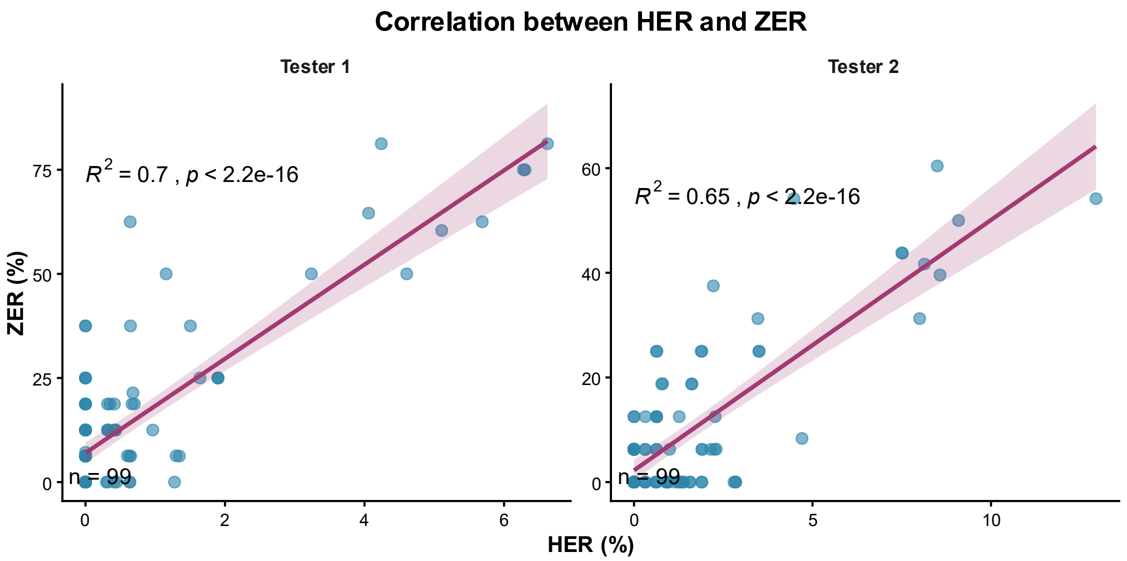

Figure S8. Correlation between haploid editing rate (HER) and zygote editing rate (ZER). Scatter plots showing the positive correlation between ZER and HER for Tester 1 (left) and Tester 2 (right). Each point represents an individual editing event targeting either *Wx1* or *Gl2* (n = 99 per tester). The solid line represents the linear regression fit, with shaded area indicating 95% confidence interval. R^2 indicates the proportion of HER variance explained by ZER. Both correlations are highly significant (Tester 1: $R^2 = 0.70$, $P < 0.0001$; Tester 2: $R^2 = 0.65$, $P < 0.0001$).

Table S1. The haploid inducer (SYN- INBD56) transformation with LbCas12aV Vectors

Experiment ID	Vector ID	LbCas12aV promoter	Variety	Start Date	End Date	Selectable Marker	Explant No.	Positive event No.	Transformation Frequency (TF%)
CT Exp.1	27145	*prZmRZDP*	SYN- INBD56	2022/3/31	2022/8/10	PMI	150	2	1.33%
CT Exp.2	27145	*prZmRZDP*	SYN- INBD56	2022/6/3	2022/9/22	PMI	940	25	2.66%
CT Exp.3	27145	*prZmRZDP*	SYN- INBD56	2022/5/30	2022/9/2	PMI	550	12	2.18%
CT Exp.4	27145	*prZmRZDP*	SYN- INBD56	2022/6/6	2022/10/6	PMI	795	20	2.52%
CT Exp.5	27145	*prZmRZDP*	SYN- INBD56	2022/7/1	2022/10/6	PMI	980	50	5.10%
CT Exp.6	27145	*prZmRZDP*	SYN- INBD56	2022/8/26	2022/11/17	PMI	680	28	4.12%
CT Exp.7	27146	*prZmVSP*	SYN- INBD56	2021/12/20	2022/5/11	PMI	255	4	1.57%
CT Exp.8	27146	*prZmVSP*	SYN- INBD56	2022/1/4	2022/9/7	PMI	125	3	2.40%
CT Exp.9	27146	*prZmVSP*	SYN- INBD56	2022/1/11	2022/4/20	PMI	690	49	7.10%
CT Exp.10	27146	*prZmVSP*	SYN- INBD56	2022/8/11	2022/11/16	PMI	1,206	23	1.91%
CT Exp.11	27146	*prZmVSP*	SYN- INBD56	2022/8/26	2022/11/9	PMI	1,230	49	3.98%
CT Exp.12	27680	*prSoUbi4*	SYN- INBD56	2022/9/8	2022/11/16	PMI	1,232	54	4.38%
CT Exp.13	27680	*prSoUbi4*	SYN- INBD56	2022/10/24	2023/2/22	PMI	375	25	6.67%
CT Exp.14	27146	*prZmVSP*	SYN- INBD56	2022/8/29	2022/12/27	PMI	764	18	2.36%
	28255	*eBSV-prZmVSP*	SYN- INBD56	2022/10/18	2023/1/30	PMI	1,016	67	6.59%
CT Exp.15	28255	*eBSV-prZmVSP*	SYN- INBD56	2022/12/7	2023/3/29	PMI	390	30	7.69%
CT Exp.16	28255	*eBSV-prZmVSP*	SYN- INBD56	2022/12/12	2023/3/21	PMI	1,010	38	3.76%
CT Exp.17	28291	*eMMV-prZmVSP*	SYN- INBD56	2023/2/3	2023/3/20	PMI	550	174	31.64%
CT Exp.18	28292	*eBSV-prZmVSP’*	SYN- INBD56	2022/12/19	2023/3/21	PMI	1,012	50	4.94%
CT Exp.19	28292	*eBSV-prZmVSP’*	SYN- INBD56	2022/12/20	2023/3/21	PMI	450	55	12.22%
**Total / Mean**							**14,400**	**776**	**5.76%**

Table S2. Taqman copy number of selected T0 events for HI-Edit test

Vector	Event	Taqman qPCR assay			
		LbCas12aV 3633 Copy_Number	LbCas12aV 3633 raw_copy	PMI 1393 Copy_Number	PMI 1393 raw_copy
	1-copy control	1.01	1	0.94	1.00
	1-copy control	0.99	1	0.88	1.00
	1-copy control	1.03	1	0.84	1.00
	1-copy control	0.86	1	0.84	1.00
	wild type	0.00	0.00	0.00	0.00
	wild type	0.00	0.00	0.00	0.00
27145	E1	1	1.05	0 or 1	0.42
27145	E2	1	0.87	1	0.84
27145	E3	>2	3.62	>2	2.92
27145	E4	1	1.05	1	0.96
27145	E5	2	1.83	2	2.05
27145	E6	1	0.97	1	0.74
27146	E1	1	1.05	1	1.05
27146	E2	1	0.95	1	1.01
27146	E3	1	0.95	1	0.88
27146	E4	1	0.92	1	0.94
27146	E5	1	0.93	1	0.97
27146	E6	1	1.08	1	1.09
27146	E7	1	0.94	1	0.92
27146	E8	1	1.17	1	0.82
27146	E9	1	1.03	1	0.83
27146	E10	1	0.96	1	0.86
27146	E11	>2	3.33	2	2.20
27680	E1	1	0.93	1	1.07
27680	E2	1	0.93	1	0.95
27680	E3	1	1.08	1	0.94
27680	E4	1	1.01	1	0.98
27680	E5	1	0.96	1	0.99
27680	E6	1	1.15	1	0.99
27680	E7	1	0.89	1	1.01
27680	E8	1	1.07	2	1.72
27680	E9	1	0.98	1	1.01
28255	E1	1	1.03	1	1.10
28255	E2	2	1.72	2	2.14
28255	E3	1	1.03	1	1.03
28291	E1	1	1.20	1	0.93
28291	E2	2	1.70	1 or 2	1.59
28291	E3	1	0.96	1	0.99
28292	E1	1	1.00	1	1.12
28292	E2	1 or 2	1.52	1	1.00
28292	E2	1 or 2	1.52	1	1.00

Table S3. Molecular analysis on transgene presence in Putative Haploids (PH*)

Vector ID	LbCas12aV promoter	Sample ID	LbCas12aV 3633 Copy Number	LbCas12aV 3633 Raw copy
		1-copy control	1	1.10
		1-copy control	1	1.04
		1-copy control	1	1.20
		1-copy control	1	1.12
		1-copy control	1	1.09
		1-copy control	1	1.05
		1-copy control	1	1.03
		1-copy control	1	1.03
		wild type	0	0.00
		wild type	0	0.00
		wild type	0	0.00
		wild type	0	0.00
27145	*prZmRZDP*	Tester 1/E3-PH01	0	0.00
27145	*prZmRZDP*	Tester 1/E3-PH02	0	0.04
27145	*prZmRZDP*	Tester 1/E3-PH03	0	0.01
27145	*prZmRZDP*	Tester 1/E3-PH04	0	0.01
27145	*prZmRZDP*	Tester 1/E3-PH05	0	0.00
27145	*prZmRZDP*	Tester 1/E2-PH01	0	0.00
27145	*prZmRZDP*	Tester 1/E2-PH02	0	0.00
27145	*prZmRZDP*	Tester 1/E2-PH03	0	0.00
27145	*prZmRZDP*	Tester 1/E2-PH04	0	0.00
27145	*prZmRZDP*	Tester 1/E2-PH05	0	0.00
27145	*prZmRZDP*	Tester 1/E6-PH01	0	0.00
27145	*prZmRZDP*	Tester 1/E6-PH02	0	0.00
27145	*prZmRZDP*	Tester 1/E6-PH03	0	0.00
27145	*prZmRZDP*	Tester 1/E6-PH04	0	0.00
27145	*prZmRZDP*	Tester 1/E6-PH05	0	0.00
27146	*prZmVSP*	Tester 1/E11-PH01	0	0.00
27146	*prZmVSP*	Tester 1/E11-PH02	0	0.00
27146	*prZmVSP*	Tester 1/E11-PH03	0	0.00
27146	*prZmVSP*	Tester 1/E11-PH04	0	0.00
27146	*prZmVSP*	Tester 1/E11-PH05	0	0.01
27146	*prZmVSP*	Tester 1/E10-PH01	0	0.00
27146	*prZmVSP*	Tester 1/E10-PH02	0	0.00
27146	*prZmVSP*	Tester 1/E10-PH03	0	0.00
27146	*prZmVSP*	Tester 1/E10-PH04	0	0.00
27146	*prZmVSP*	Tester 1/E10-PH05	0	0.00
27146	*prZmVSP*	Tester 1/E9-PH01	0	0.00
27146	*prZmVSP*	Tester 1/E9-PH02	0	0.00
27146	*prZmVSP*	Tester 1/E9-PH03	0	0.00
27146	*prZmVSP*	Tester 1/E9-PH04	0	0.00
27146	*prZmVSP*	Tester 1/E9-PH05	0	0.00
27680	*prSoUbi4*	Tester 1/E6-PH01	0	0.00
27680	*prSoUbi4*	Tester 1/E6-PH02	0	0.00
27680	*prSoUbi4*	Tester 1/E6-PH03	0	0.00
27680	*prSoUbi4*	Tester 1/E6-PH04	0	0.00
27680	*prSoUbi4*	Tester 1/E6-PH05	1	0.90
27680	*prSoUbi4*	Tester 1/E8-PH01	0	0.00
27680	*prSoUbi4*	Tester 1/E8-PH02	0	0.00
27680	*prSoUbi4*	Tester 1/E8-PH03	0	0.00
27680	*prSoUbi4*	Tester 1/E8-PH04	0	0.00
27680	*prSoUbi4*	Tester 1/E8-PH05	0	0.00
27680	*prSoUbi4*	Tester 1/E7-PH01	0	0.00
27680	*prSoUbi4*	Tester 1/E7-PH02	0	0.00
27680	*prSoUbi4*	Tester 1/E7-PH03	0	0.00
27680	*prSoUbi4*	Tester 1/E7-PH04	0	0.00
27680	*prSoUbi4*	Tester 1/E7-PH05	0	0.00
28291	*eMMV-prZmVSP*	Tester 1/E2-PH01	0	0.00
28291	*eMMV-prZmVSP*	Tester 1/E2-PH02	0	0.00
28291	*eMMV-prZmVSP*	Tester 1/E2-PH03	0	0.00
28291	*eMMV-prZmVSP*	Tester 1/E1-PH01	0	0.00
28291	*eMMV-prZmVSP*	Tester 1/E1-PH02	0	0.01
28291	*eMMV-prZmVSP*	Tester 1/E1-PH03	0	0.00
28291	*eMMV-prZmVSP*	Tester 1/E3-PH01	0	0.00
28291	*eMMV-prZmVSP*	Tester 1/E3-PH02	0	0.00
28291	*eMMV-prZmVSP*	Tester 1/E3-PH03	0	0.00
28255	*eBSV-prZmVSP*	Tester 1/E2-PH01	0	0.04
28255	*eBSV-prZmVSP*	Tester 1/E2-PH02	0	0.00
28255	*eBSV-prZmVSP*	Tester 1/E2-PH03	0	0.00
28255	*eBSV-prZmVSP*	Tester 1/E3-PH01	0	0.00
28255	*eBSV-prZmVSP*	Tester 1/E3-PH02	0	0.01
28255	*eBSV-prZmVSP*	Tester 1/E3-PH03	0	0.00
28255	*eBSV-prZmVSP*	Tester 1/E1-PH01	0	0.01
28255	*eBSV-prZmVSP*	Tester 1/E1-PH02	0	0.00
28255	*eBSV-prZmVSP*	Tester 1/E1-PH03	0	0.00
28292	*eMMV-prZmVSP'*	Tester 1/E1-PH01	0	0.00
28292	*eMMV-prZmVSP'*	Tester 1/E1-PH02	0	0.00
28292	*eMMV-prZmVSP'*	Tester 1/E1-PH03	0	0.00
28292	*eMMV-prZmVSP'*	Tester 1/E2-PH01	0	0.00
28292	*eMMV-prZmVSP'*	Tester 1/E2-PH02	0	0.00
28292	*eMMV-prZmVSP'*	Tester 1/E2-PH03	0	0.00

PH*: putative haploids by color sorting

Table S4. The Haploid Induction Rate (HIR) % of inducer (SYN- INBD56) events transformed by LbCas12aV Vectors

Trial ID	Vector ID	LbCas12aV promoter	Event No.	Tester	F1 ear No.	F1 embryo No.	Haploid No.	Haploid Induction Rate (HIR) %
#1	27145	*prZmRZDP*	6	Tester 1	99	16,143	2,255	13.97%
				Tester 2	107	16,001	2,273	14.21%
	27146	*prZmVSP*	11	Tester 1	188	31,370	3,501	11.16%
				Tester 2	193	33,362	4,034	12.09%
	27680	*prSoUbi4*	9	Tester 1	136	22,707	2,594	11.42%
				Tester 2	141	23,941	2,931	12.24%
#2	27145	*prZmRZDP*	3	Tester 1	23	1,702	182	10.69%
				Tester 2	9	866	106	12.24%
	27146	*prZmVSP*	3	Tester 1	20	1,837	177	9.64%
				Tester 2	23	3,852	421	10.93%
	27680	*prSoUbi4*	3	Tester 1	21	948	91	9.60%
				Tester 2	12	1,360	185	13.60%
	28255	*eBSV-prZmVSP*	3	Tester 1	49	7,516	838	11.15%
				Tester 2	34	4,989	726	14.55%
	28291	*eMMV-prZmVSP*	3	Tester 1	47	7,024	681	9.70%
				Tester 2	42	6,407	826	12.89%
	28292	*eBSV-prZmVSP'*	2	Tester 1	30	5,077	589	11.60%
				Tester 2	33	4,864	658	13.53%

Table S5. The haploid editing rate (HER) and zygote editing rate (ZER) of HI-Edit vectors and events

Vector ID	LbCas12aV promoter	Event ID	Tester	Haploid No.	HER_*Wx1*	HER_*Gl2*	Both target edited _Haploid No.	HER_*Wx1* + Gl2	F1 hybrid	ZER_*Wx1*	ZER_*Gl2*
27145	*prZmRZDP*	E1	Tester1	316	0.00%	0.00%	0	0.00%	16	0.00%	0.00%
27145	*prZmRZDP*	E2	Tester1	317	0.32%	0.00%	0	0.00%	16	18.75%	0.00%
27145	*prZmRZDP*	E3	Tester1	316	0.00%	0.32%	0	0.00%	16	0.00%	0.00%
27145	*prZmRZDP*	E4	Tester1	300	0.67%	0.00%	0	0.00%	16	18.75%	0.00%
27145	*prZmRZDP*	E5	Tester1	312	0.64%	0.00%	0	0.00%	16	62.50%	6.25%
27145	*prZmRZDP*	E6	Tester1	42	0.00%	0.00%	0	0.00%	16	6.25%	0.00%
27145	*prZmRZDP*	E1	Tester2	316	0.63%	0.00%	0	0.00%	16	12.50%	6.25%
27145	*prZmRZDP*	E2	Tester2	312	0.64%	0.00%	0	0.00%	16	25.00%	6.25%
27145	*prZmRZDP*	E3	Tester2	319	0.00%	0.00%	0	0.00%	16	0.00%	0.00%
27145	*prZmRZDP*	E4	Tester2	314	3.50%	0.00%	0	0.00%	16	25.00%	6.25%
27145	*prZmRZDP*	E5	Tester2	317	3.47%	0.00%	0	0.00%	16	31.25%	6.25%
27145	*prZmRZDP*	E6	Tester2	129	0.78%	0.00%	0	0.00%	16	18.75%	6.25%
27146	*prZmVSP*	E1	Tester1	311	0.96%	0.00%	0	0.00%	16	12.50%	0.00%
27146	*prZmVSP*	E2	Tester1	317	0.32%	0.00%	0	0.00%	16	12.50%	0.00%
27146	*prZmVSP*	E3	Tester1	294	0.68%	0.00%	0	0.00%	14	21.43%	7.14%
27146	*prZmVSP*	E4	Tester1	310	0.65%	0.00%	0	0.00%	16	37.50%	0.00%
27146	*prZmVSP*	E5	Tester1	249	0.00%	0.00%	0	0.00%	16	18.75%	0.00%
27146	*prZmVSP*	E6	Tester1	90	0.00%	0.00%	0	0.00%	16	25.00%	0.00%
27146	*prZmVSP*	E7	Tester1	302	0.33%	0.00%	0	0.00%	16	12.50%	0.00%
27146	*prZmVSP*	E8	Tester1	315	0.32%	0.00%	0	0.00%	16	12.50%	0.00%
27146	*prZmVSP*	E9	Tester1	314	0.00%	0.00%	0	0.00%	16	6.25%	0.00%
27146	*prZmVSP*	E10	Tester1	309	0.65%	0.00%	0	0.00%	16	6.25%	0.00%
27146	*prZmVSP*	E11	Tester1	223	6.28%	1.35%	0	0.00%	16	75.00%	6.25%
27146	*prZmVSP*	E1	Tester2	316	1.27%	0.32%	0	0.00%	16	12.50%	0.00%
27146	*prZmVSP*	E2	Tester2	320	0.31%	0.00%	0	0.00%	16	6.25%	0.00%
27146	*prZmVSP*	E3	Tester2	317	0.60%	0.00%	0	0.00%	16	12.50%	0.00%
27146	*prZmVSP*	E4	Tester2	319	0.64%	0.31%	0	0.00%	16	25.00%	6.25%
27146	*prZmVSP*	E5	Tester2	320	0.61%	0.00%	0	0.00%	16	0.00%	0.00%
27146	*prZmVSP*	E6	Tester2	56	0.00%	0.00%	0	0.00%	16	0.00%	6.25%
27146	*prZmVSP*	E7	Tester2	320	0.60%	0.00%	0	0.00%	16	0.00%	0.00%
27146	*prZmVSP*	E8	Tester2	318	0.62%	0.00%	0	0.00%	16	6.25%	0.00%
27146	*prZmVSP*	E9	Tester2	316	0.95%	0.95%	0	0.00%	16	0.00%	0.00%
27146	*prZmVSP*	E10	Tester2	319	1.88%	0.00%	0	0.00%	16	25.00%	0.00%
27146	*prZmVSP*	E11	Tester2	133	7.52%	2.26%	0	0.00%	16	43.75%	6.25%
27680	*prSoUbi4*	E1	Tester1	223	0.00%	0.45%	0	0.00%	16	12.50%	0.00%
27680	*prSoUbi4*	E2	Tester1	264	0.00%	0.00%	0	0.00%	16	18.75%	6.25%
27680	*prSoUbi4*	E3	Tester1	318	0.00%	0.00%	0	0.00%	16	37.50%	12.50%
27680	*prSoUbi4*	E4	Tester1	315	0.63%	0.00%	0	0.00%	16	0.00%	0.00%
27680	*prSoUbi4*	E5	Tester1	315	0.00%	0.00%	0	0.00%	16	6.25%	12.50%
27680	*prSoUbi4*	E6	Tester1	316	1.90%	0.00%	0	0.00%	16	25.00%	6.25%
27680	*prSoUbi4*	E7	Tester1	232	0.43%	0.00%	0	0.00%	16	12.50%	0.00%
27680	*prSoUbi4*	E8	Tester1	162	0.00%	0.00%	0	0.00%	16	0.00%	0.00%
27680	*prSoUbi4*	E9	Tester1	317	0.00%	0.00%	0	0.00%	16	25.00%	12.50%
27680	*prSoUbi4*	E1	Tester2	145	1.38%	0.00%	0	0.00%	16	0.00%	6.25%
27680	*prSoUbi4*	E2	Tester2	315	1.90%	0.32%	0	0.00%	16	6.25%	12.50%
27680	*prSoUbi4*	E3	Tester2	319	1.57%	0.63%	0	0.00%	16	0.00%	6.25%
27680	*prSoUbi4*	E4	Tester2	309	1.62%	0.00%	0	0.00%	16	18.75%	6.25%
27680	*prSoUbi4*	E5	Tester2	316	1.90%	0.63%	0	0.00%	16	0.00%	12.50%
27680	*prSoUbi4*	E6	Tester2	316	2.85%	0.32%	0	0.00%	16	0.00%	0.00%
27680	*prSoUbi4*	E7	Tester2	317	1.26%	0.00%	0	0.00%	16	0.00%	0.00%
27680	*prSoUbi4*	E8	Tester2	185	0.00%	0.00%	0	0.00%	15	0.00%	0.00%
27680	*prSoUbi4*	E9	Tester2	318	0.94%	0.00%	0	0.00%	16	0.00%	12.50%
28255	*eBSV-prZmVSP*	E1	Tester1	241	0.41%	0.00%	0	0.00%	16	18.75%	0.00%
28255	*eBSV-prZmVSP*	E2	Tester1	244	1.64%	0.41%	1	0.41%	16	25.00%	0.00%
28255	*eBSV-prZmVSP*	E3	Tester1	316	0.63%	0.00%	0	0.00%	16	6.25%	6.25%
28255	*eBSV-prZmVSP*	E1	Tester2	186	2.15%	0.00%	0	0.00%	16	6.25%	0.00%
28255	*eBSV-prZmVSP*	E2	Tester2	225	2.22%	0.00%	0	0.00%	16	37.50%	12.50%
28255	*eBSV-prZmVSP*	E3	Tester2	301	1.00%	0.00%	0	0.00%	16	6.25%	0.00%
28291	*eMMV-prZmVSP*	E1	Tester1	257	0.00%	0.00%	0	0.00%	16	6.25%	6.25%
28291	*eMMV-prZmVSP*	E2	Tester1	173	1.16%	0.00%	0	0.00%	16	50.00%	6.25%
28291	*eMMV-prZmVSP*	E3	Tester1	235	1.28%	0.00%	0	0.00%	16	0.00%	0.00%
28291	*eMMV-prZmVSP*	E1	Tester2	314	0.64%	0.00%	0	0.00%	16	0.00%	0.00%
28291	*eMMV-prZmVSP*	E2	Tester2	308	2.27%	0.00%	0	0.00%	16	12.50%	6.25%
28291	*eMMV-prZmVSP*	E3	Tester2	175	1.14%	0.00%	0	0.00%	16	0.00%	0.00%
28292	*eBSV-prZmVSP'*	E1	Tester1	266	1.50%	0.00%	0	0.00%	16	37.50%	0.00%
28292	*eBSV-prZmVSP'*	E2	Tester1	288	0.00%	0.00%	0	0.00%	16	12.50%	18.75%
28292	*eBSV-prZmVSP'*	E1	Tester2	232	0.00%	0.00%	0	0.00%	16	0.00%	0.00%
28292	*eBSV-prZmVSP'*	E2	Tester2	313	0.00%	0.00%	0	0.00%	16	0.00%	0.00%

Table S6. Genotype of target genes in edited haploids by NGS

Gene target	Samples and Editing analysis	Total Read Number	Sequence
Wx1	Tester 2 (WT)	N/A	GCCTGGTCGCTGGTTTCAGG**TTTG**GGGAAAGACCGAGGAGAAGATCTACGGGCCTG
Wx1	27145-E4-Tester2-01_(-15,_93.8%)	8,741	GCCTGGTCGCTGGTTTCAGGTTTGGGGAAAGAC---------------CGGGCCTG
Wx1	27145-E4-Tester2-02_(-21,_93.1%)	10,153	GCCTGGTCGCTGGTTTCAGGTTTGGGGAA---------------------GGCCTG
Wx1	27145-E4-Tester2-03_(-3,_37.7%)	7,377	GCCTGGTCGCTGGTTTCAGGTTTGGGGAAAGACCGAGG---AGATCTACGGGCCTG
Wx1	27145-E4-Tester2-04_(-26,_34%)	5,493	GCCTGGTCGCTGGTTTCAGGTTT--------------------------GGGCCTG
Wx1	27145-E4-Tester2-05-1_(-13,_50%)	9,570	GCCTGGTCGCTGGTTTCAGGTTTGGGGAAAGACCGA-------------GGGCCTG
Wx1	27145-E4-Tester2-05-2_(-34,_25.5%)	9,570	GCCTGGTCGCTGGTTT----------------------------------GGCCTG
Wx1	27145-E4-Tester2-06-1_(-11,_54%)	11,148	GCCTGGTCGCTGGTTTCAGGTTTGGGGAAAGACCGAG-----------CGGGCCTG
Wx1	27145-E4-Tester2-06-2_(-7,_27.4%)	11,148	GCCTGGTCGCTGGTTTCAGGTTTGGGGAAAGACCGAGGA-------TCCGGGCCTG
Wx1	27145-E4-Tester2-07-1_(-19,_33.1%)	10,030	GCCTGGTCGCTGGTTTCAGGTTTGGGGAAAGACCGAG-------------------
Wx1	27145-E4-Tester2-07-2_(-19,_11%)	10,030	GCCTGGTCGCTGGTTTCAGGTTTGGGGAAAGACCGAG-------------------
Wx1	27145-E4-Tester2-08_(-26,_53.8%)	7,745	GCCTGGTCGCTGGTTTCAGGTT--------------------------CGGGCCTG
Wx1	27145-E4-Tester2-09_(-12,_93.7%)	10,218	GCCTGGTCGCTGGTTTCAGGTTTGGGGAAAGACCG------------ACGGGCCTG
Wx1	27145-E4-Tester2-10_(-72,_95.9%)	11,820	--TACGGGCCTG
Wx1	27145-E4-Tester2-11_(-13,_92.5%)	8,392	GCCTGGTCGCTGGTTTCAGGTTTGGGGAAAGACCGA-------------GGGCCTG
Wx1	27145-E5-Tester2-01-1_(-12,_45.6%)	10,354	GCCTGGTCGCTGGTTTCAGGTTTGGGGAAAGAC------------CTACGGGCCTG
Wx1	27145-E5-Tester2-01-2_(-25,_26.1%)	10,354	GCCTGGTCGCTGGTTTCAGGTTTGGGGAAAGA------------------------
Wx1	27145-E5-Tester2-01-3_(-10,_15.7%)	10,354	GCCTGGTCGCTGGTTTCAGGTTTGGGGAAAGACCGA----------CACTGGCCTG
Wx1	27145-E5-Tester2-02-1_(-12,_35.2%)	8,938	GCCTGGTCGCTGGTTTCAGGTTTGGGGAAAGACCGAG------------GGGCCTG
Wx1	27145-E5-Tester2-02-2_(-8,_33.4%)	8,938	GCCTGGTCGCTGGTTTCAGGTTTGGGGAAAGACCGAGGA--------ACGGGCCTG
Wx1	27145-E5-Tester2-03-1_(-36,_44.9%)	7,182	GCCTGGTCGCTGGTTTCAGGTTTGG------------------------------
Wx1	27145-E5-Tester2-03-2_(-13,_39.4%)	7,182	GCCTGGTCGCTGGTTTCAGGTTTGGGGAAAGACCGA-------------GGGCCTG
Wx1	27145-E5-Tester2-04_(-14,_22.8%)	10,559	GCCTGGTCGCTGGTTTCAGGTTTGGGGAAAGACCGA--------------GGCCTG
Wx1	27145-E5-Tester2-05_(-26,_29.6%)	9,850	GCCTGGTCGCTGGTTTCAGGTTT--------------------------GGGCCTG
Wx1	27145-E5-Tester2-06_(-16,_95.9%)	11,180	GCCTGGTCGCTGGTTTCAGGTTTGGGGAAAGACC----------------GGCCTG
Wx1	27145-E5-Tester2-07_(-48,_57.3%)	8,826	GCCTGGTCGCTGGTTTCAGGTTTGGGGAAAGACCG---------------------
Wx1	27145-E5-Tester2-08_(-15,_18.5%)	12,004	GCCTGGTCGCTGGTTTCAGGTTTGGGGAAAGAC---------------CGGGCCTG
Wx1	27145-E5-Tester2-09_(-21,_79.4%)	10,333	GCCTGGTCGCTGGTTTCAGGTTTGGGGAA---------------------GGCCTG
Wx1	27145-E5-Tester2-10-1_(-3,_62.6%)	8,681	GCCTGGTCGCTGGTTTCAGGTTTGGGGAAAGACCGAGG---AGATCTACGGGCCTG
Wx1	27145-E5-Tester2-10-2_(-26,_19.2%)	8,681	GCCTGGTCGCTGGTTTCAGG--------------------------TACGGGCCTG
Wx1	27145-E5-Tester2-11_(-7,_52.2%)	9,319	GCCTGGTCGCTGGTTTCAGGTTTGGGGAAAGACCGAGGAG-------ACGGGCCTG
Wx1	27146-E11-Tester2-01_(-13,_93.2%)	13,216	GCCTGGTCGCTGGTTTCAGGTTTGGGGAAAGACCG-------------CGGGCCTG
Wx1	27146-E11-Tester2-02_(-2,_60.2%)	11,198	GCCTGGTCGCTGGTTTCAGGTTTGGGGAAAGACCGAG--GAAGATCTACGGGCCTG
Wx1	27146-E11-Tester2-03_(-12,_60.5%)	10,961	GCCTGGTCGCTGGTTTCAGGTTTGGGGAAAGACCGAG------------GGGCCTG
Wx1	27146-E11-Tester2-04_(-2,_91.9%)	9,155	GCCTGGTCGCTGGTTTCAGGTTTGGGGAAAGACCGAG--GAAGATCTACGGGCCTG
Wx1	27146-E11-Tester2-05_(-12,_91.9%)	8,576	GCCTGGTCGCTGGTTTCAGGTTTGGGGAAAGACCG------------ACGGGCCTG
Wx1	27146-E11-Tester2-06-1_(-3,_41.2%)	13,597	GCCTGGTCGCTGGTTTCAGGTTTGGGGAAAGACCGAGG---AGATCTACGGGCCTG
Wx1	27146-E11-Tester2-06-2_(-8,_40.3%)	13,597	GCCTGGTCGCTGGTTTCAGGTTTGGGGAAAGACCGAGG--------TACGGGCCTG
Wx1	27146-E11-Tester2-07_(-12,_80.2%)	9,633	GCCTGGTCGCTGGTTTCAGGTTTGGGGAAAGACCGAG------------GGGCCTG
Wx1	27146-E11-Tester2-08_(-5,_90.8%)	9,423	GCCTGGTCGCTGGTTTCAGGTTTGGGGAAAGACCGAG-----GATCTACGGGCCTG
Wx1	27146-E11-Tester2-09_(-7,_36.1%)	10,562	GCCTGGTCGCTGGTTTCAGGTTTGGGGAAAGACCGAGGAG-------ACGGGCCTG
Wx1	27146-E11-Tester2-10_(-9,_91.2%)	8,600	GCCTGGTCGCTGGTTTCAGGTTTGGGGAAAGACCGAGG---------ACGGGCCTG
Wx1	27680-E6-Tester2-01_(-9,_36.8%)	12,429	GCCTGGTCGCTGGTTTCAGGTTTGGGGAAAGACCGAGG---------ACGGGCCTG
Wx1	27680-E6-Tester2-02_(-14,_92.4%)	9,064	GCCTGGTCGCTGGTTTCAGGTTTGGGGAAAGACCG--------------GGGCCTG
Wx1	27680-E6-Tester2-03_(-33,_88.5%)	14,944	GCCTGGTCGCTGGTTTCA---------------------------------GCCTG
Wx1	27680-E6-Tester2-04_(-24,_88.8%)	15,503	GCCTGGTCGCTGGTTTCAGGTTTGG-----------------------GGGCCTG
Wx1	27680-E6-Tester2-05-1_(-72,_21.4%)	11,354	--TACGGGCCTG
Wx1	27680-E6-Tester2-05-2_(-18,_15.6%)	11,354	GCCTGGTCGCTGGTTTCAGGTTTGGGGAA------------------ACGGGCCTG
Wx1	27680-E6-Tester2-05-3_(-26,_11.9%)	11,354	GCCTGGTCGCTGGTTTCAGGTTT--------------------------GGGCCTG
Wx1	27680-E6-Tester2-06-1_(-12,_32%)	9,722	GCCTGGTCGCTGGTTTCAGGTTTGGGGAAAGACCGAG------------GGGCCTG
Wx1	27680-E6-Tester2-07-1_(-13,_53.6%)	8,697	GCCTGGTCGCTGGTTTCAGGTTTGGGGAAAGACCGA-------------GGGCCTG
Wx1	27680-E6-Tester2-07-2_(-82,_10.4%)	8,697	GCCTGGTCGCTGGTTTCAGGTTTGGGGAAAGACCGA--------------------
Wx1	27680-E6-Tester2-08-1_(-12,_36%)	10,014	GCCTGGTCGCTGGTTTCAGGTTTGGGGAAAGACCGAG------------GGGCCTG
Wx1	27680-E6-Tester2-08-2_(-11,_16.2%)	10,014	GCCTGGTCGCTGGTTTCAGGTTTGGGGAAAGACCGAG-----------CGGGCCTG
Wx1	27680-E6-Tester2-08-3_(-15,_10.2%)	10,014	GCCTGGTCGCTGGTTTCAGGTTTGGGGAAAGAC---------------CGGGCCTG
Wx1	27680-E6-Tester2-09_(-6,_87.7%)	8,029	GCCTGGTCGCTGGTTTCAGGTTTGGGGAAAGACCGAGGAG------TACGGGCCTG
Gl2	Tester 2 (WT)	N/A	TTTTTTATTTATCGTTTC**TTTG**GTCACAGATCACAAACTTCAAATGCGGTGGGCTGGC
Gl2	27146-E11-Tester2-01_(-9,_84.6%)	6,494	TTTTTTATTTATCGTTTCTTTGGTCACAGATCACAAAC---------GGTGGGCTGGC
Gl2	27146-E11-Tester2-02_(-41,_89.5%)	9,132	TTTTTTATTTATCGTTTCTTTGG-----------------------------------
Gl2	27146-E11-Tester2-03_(-1,_92.6%)	10,336	TTTTTTATTTATCGTTTCTTTGGTCACAGATCACAA-CTTCAAATGCGGTGGGCTGGC
Gl2	27680-E6-Tester2-01_(-6,_89.9%)	7,684	TTTTTTATTTATCGTTTCTTTGGTCACAGATCACAAACT------GCGGTGGGCTGGC

Table S7. Genotype of target genes in edited F1 diploids

Gene target	Samples and Editing analysis	Total Read Number	Sequence
Wx1	Tester1(WT)	N/A	GCCTGGTCGCTGGTTTCAGG**TTTG**GGGAAAGACCGAGGAGAAGATCTACGGGCCTG
Wx1	Tester2 (WT)	N/A	GCCTGGTCGCTGGTTTCAGG**TTTG**GGGAAAGACCGAGGAGAAGATCTACGGGCCTG
Wx1	27145-E4-Tester2-01- allele 1_(-9,_57.6%)	6,961	GCCTGGTCGCTGGTTTCAGGTTTGGGGAAAGACCGAGG---------ACGGGCCTG
Wx1	27145-E4-Tester2-01- allele 2_(-1,_31.3%)	6,961	GCCTGGTCGCTGGTTTCAGGTTTGGGGAAAGACCGA-GAGAAGATCTACGGGCCTG
Wx1	27145-E4-Tester2-02- allele 1_(-9,_51.5%)	10,458	GCCTGGTCGCTGGTTTCAGGTTTGGGGAAAGACCGAGG---------ACGGGCCTG
Wx1	27145-E4-Tester2-02- allele 2_(-4,_34.8%)	10,458	GCCTGGTCGCTGGTTTCAGGTTTGGGGAAAGACCGAG----AGATCTACGGGCCTG
Wx1	27145-E4-Tester2-03- allele 1_(-5,_50.2%)	7,592	GCCTGGTCGCTGGTTTCAGGTTTGGGGAAAGACCGAG-----GATCTACGGGCCTG
Wx1	27145-E4-Tester2-03- allele 2_(-3,_35.4%)	7,592	GCCTGGTCGCTGGTTTCAGGTTTGGGGAAAGACCGAGG---AGATCTACGGGCCTG
Wx1	27145-E4-Tester2-04- allele 1_(-9,_51.1%)	11,264	GCCTGGTCGCTGGTTTCAGGTTTGGGGAAAGACCGAGG---------ACGGGCCTG
Wx1	27145-E4-Tester2-04- allele 2_(-8,_30.7%)	11,264	-------ATTGAGATGAGATCTGATCACTCGATACGCAATTACCACCCCATCCTGA
Wx1	27145-E5-Tester2-01- allele 1_(-15,_53.2%)	12,700	GCCTGGTCGCTGGTTTCAGGTTTGGGGAAAGAC---------------CGGGCCTG
Wx1	27145-E5-Tester2-01- allele 2_(-6,_30.4%)	12,700	GCCTGGTCGCTGGTTTCAGGTTTGGGGAAAGACCGAGGAGA------ACGGGCCTG
Wx1	27145-E5-Tester2-02- allele 1_(-5,_51.1%)	9,556	GCCTGGTCGCTGGTTTCAGGTTTGGGGAAAGACCGAGGAG-----CTACGGGCCTG
Wx1	27145-E5-Tester2-02- allele 2_(-13,_31.2%)	9,556	GCCTGGTCGCTGGTTTCAGGTTTGGGGAAAGACCGA-------------GGGCCTG
Wx1	27145-E5-Tester2-03- allele 1_(-5,_46.8%)	12,679	GCCTGGTCGCTGGTTTCAGGTTTGGGGAAAGACCGAGGAG-----CTACGGGCCTG
Wx1	27145-E5-Tester2-03- allele 2_(-12,_37.7%)	12,679	GCCTGGTCGCTGGTTTCAGGTTTGGGGAAAGACCGAG------------GGGCCTG
Wx1	27145-E5-Tester2-04- allele 1_(-5,_49.5%)	9,699	GCCTGGTCGCTGGTTTCAGGTTTGGGGAAAGACCGAGGAG-----CTACGGGCCTG
Wx1	27145-E5-Tester2-04- allele 2_(-5,_34.7%)	9,699	GCCTGGTCGCTGGTTTCAGGTTTGGGGAAAGACCGAGGAGA-----TACGGGCCTG
Wx1	27145-E5-Tester2-05- allele 1_(-5,_42.6%)	10,477	GCCTGGTCGCTGGTTTCAGGTTTGGGGAAAGACCGAGGAG-----CTACGGGCCTG
Wx1	27145-E5-Tester2-05- allele 2_(-51,_38.1%)	10,477	---GCCTG
Wx1	27146-E11-Tester2-01- allele 1_(-15,_54.6%)	6,355	GCCTGGTCGCTGGTTTCAGGTTTGGGGAAAGAC---------------CGGGCCTG
Wx1	27146-E11-Tester2-01- allele 2_(-12,_30.7%)	6,355	GCCTGGTCGCTGGTTTCAGGTTTGGGGAAAGACCGAGGA------------GCCTG
Wx1	27146-E11-Tester2-02- allele 1_(-10,_59.4%)	5,040	GCCTGGTCGCTGGTTTCAGGTTTGGGGAAAGACCGAG----------ACGGGCCTG
Wx1	27146-E11-Tester2-02- allele 2_(-11,_30.7%)	5,040	GCCTGGTCGCTGGTTTCAGGTTTGGGGAAAGACCGAGGA-----------GGCCTG
Wx1	27146-E11-Tester2-03- allele 1_(-10,_49.2%)	10,601	GCCTGGTCGCTGGTTTCAGGTTTGGGGAAAGACCGAG----------ACGGGCCTG
Wx1	27146-E11-Tester2-03- allele 2_(-2,_33.8%)	10,601	GCCTGGTCGCTGGTTTCAGGTTTGGGGAAAGACCGAG--GAAGATCTACGGGCCTG
Wx1	27146-E11-Tester2-04- allele 1_(-15,_54.7%)	5,095	GCCTGGTCGCTGGTTTCAGGTTTGGGGAAAGAC---------------CGGGCCTG
Wx1	27146-E11-Tester2-04- allele 2_(-4,_31.2%)	5,095	GCCTGGTCGCTGGTTTCAGGTTTGGGGAAAGACCGAGGAGA----TTACGGGCCTG
Wx1	27146-E11-Tester2-05- allele 1_(-15,_50.5%)	6,274	GCCTGGTCGCTGGTTTCAGGTTTGGGGAAAGAC---------------CGGGCCTG
Wx1	27146-E11-Tester2-05- allele 2_(-6,_33.5%)	6,274	GCCTGGTCGCTGGTTTCAGGTTTGGGGAAAGACCGAGGAGA---TCT---GGCCTG
Wx1	27146-E11-Tester2-06- allele 1_(-10,_55.6%)	3,606	GCCTGGTCGCTGGTTTCAGGTTTGGGGAAAGACCGAG----------ACGGGCCTG
Wx1	27146-E11-Tester2-06- allele 2_(-16,_36.1%)	3,606	GCCTGGTCGCTGGTTTCAGGTTTGGGGAAAGACC----------------GGCCTG
Wx1	27146-E11-Tester2-07- allele 1_(-15,_53.3%)	4,966	GCCTGGTCGCTGGTTTCAGGTTTGGGGAAAGAC---------------CGGGCCTG
Wx1	27146-E11-Tester2-07- allele 2_(-11,_34.6%)	4,966	GCCTGGTCGCTGGTTTCAGGTTTGGGGAAAGACCGAG-----------CGGGCCTG
Gl2	Tester1(WT)	N/A	TTTTTTATTTATCGTTTC**TTTG**GTCACAGATCACAAACTTCAAATGCGGTGGGCTGGC
Gl2	Tester2 (WT)	N/A	TTTTTTATTTATCGTTTC**TTTG**GTCACAGATCACAAACTTCAAATGCGGTGGGCTGGC
Gl2	27145-E4-Tester2-01- allele 1_(-9,_64.9%)	9,095	TTTTTTATTTATCGTTTCTTTGGTCACAGATCACAAAC---------GGTGGGCTGGC
Gl2	27145-E4-Tester2-01- allele 2_(-5,_16.3%)	9,095	TTTTTTATTTATCGTTTCTTTGGTCACAGATCACAAACTT-----GCGGTGGGCTGGC
Gl2	27145-E5-Tester2-01- allele 1_(-6,_68.8%)	11,853	TTTTTTATTTATCGTTTCTTTGGTCACAGATCACAAACTTC------GGTGGGCTGGC
Gl2	27146-E11-Tester2-01- allele 1_(-9,_93.3%)	13,658	TTTTTTATTTATCGTTTCTTTGGTCACAGATCACAA---------GCGGTGGGCTGGC

Table S8. The haploid editing rate (HER) under normal and heat treatment conditions

				Normal condition (Control)								Heat treatment							
Tester	Trial ID	Vector ID	Event ID	F1 Ear No.	Kernels /ear	Haploid No.	HIR%	HER_ *Wx1*	HER_ *Gl2*	Both target edit haploid No.	HER_ *Wx1* + *Gl2*	F1 Ear No.	Kernels /ear	Haploid No.	HIR%	HER_ *Wx1*	HER_ *Gl2*	Both target edit haploid No.	HER_ *Wx1* + *Gl2*
Tester 1	#1	27145	E2	20	159	317	9.97%	0.32%	0.00%	0	0.00%	7	121	115	13.58%	0.87%	0.00%	0	0.00%
Tester 1	#1	27145	E5	20	161	312	9.69%	0.64%	0.00%	0	0.00%	7	115	116	14.41%	2.59%	0.86%	0	0.00%
Tester 1	#1	27146	E4	22	154	310	9.15%	0.65%	0.00%	0	0.00%	9	122	112	10.20%	0.89%	0.00%	0	0.00%
Tester 1	#1	27146	E6	22	267	90	1.53%	0.00%	0.00%	0	0.00%	6	150	20	2.22%	5.00%	5.00%	1	5.00%
Tester 1	#1	27146	E7	21	181	302	7.95%	0.33%	0.00%	0	0.00%	8	138	126	11.41%	0.00%	0.00%	0	0.00%
Tester 1	#1	27680	E2	20	147	264	8.98%	0.00%	0.00%	0	0.00%	7	126	91	10.32%	2.20%	1.10%	0	0.00%
Tester 1	#1	27680	E3	21	157	318	9.65%	0.00%	0.00%	0	0.00%	8	112	125	13.95%	2.40%	1.60%	0	0.00%
Tester 1	#2	27145	E3	22	171	316	8.40%	0.00%	0.30%	0	0.00%	12	93	123	11.07%	0.81%	0.81%	1	0.81%
Tester 1	#2	27145	E4	20	154	300	9.74%	0.70%	0.00%	0	0.00%	11	54	53	8.97%	5.66%	3.77%	2	3.77%
Tester 1	#2	27146	E9	20	180	314	8.72%	0.00%	0.00%	0	0.00%	9	62	59	10.63%	1.69%	0.00%	0	0.00%
Tester 1	#2	27146	E10	22	179	309	7.85%	0.60%	0.00%	0	0.00%	11	117	105	8.19%	1.90%	0.00%	0	0.00%
Tester 1	#2	27146	E11	23	246	223	3.94%	6.30%	1.30%	2	0.90%	11	185	77	3.78%	7.79%	5.19%	1	1.30%
Tester 1	#2	27680	E6	25	149	316	8.48%	1.90%	0.00%	0	0.00%	10	46	35	7.56%	2.86%	5.71%	1	2.86%
Tester 1	#2	27680	E7	21	124	232	8.91%	0.40%	0.00%	0	0.00%	11	44	52	10.72%	1.92%	0.00%	0	0.00%
Tester 1	#2	27680	E8	23	235	162	3.00%	0.00%	0.00%	0	0.00%	12	68	11	1.34%	0.00%	0.00%	0	0.00%
Tester 2	#1	27145	E2	21	157	312	9.46%	0.64%	0.00%	0	0.00%	5	97	57	11.71%	12.28%	0.00%	0	0.00%
Tester 2	#1	27146	E7	20	182	320	8.79%	0.60%	0.00%	0	0.00%	7	89	81	12.98%	4.94%	0.00%	0	0.00%
Tester 2	#1	27680	E3	16	215	319	9.27%	1.57%	0.63%	0	0.00%	7	86	76	12.62%	7.89%	2.63%	1	1.32%
Tester 2	#2	27145	E3	21	167	319	9.10%	0.00%	0.00%	0	0.00%	9	139	85	6.81%	0.00%	0.00%	0	0.00%
Tester 2	#2	27145	E4	22	66	314	21.54%	3.50%	0.00%	0	0.00%	9	97	102	11.71%	6.86%	0.00%	0	0.00%
Tester 2	#2	27145	E6	25	266	129	1.94%	0.80%	0.00%	0	0.00%	14	199	11	0.39%	18.18%	0.00%	0	0.00%
Tester 2	#2	27146	E9	22	158	316	9.09%	0.90%	0.90%	0	0.00%	13	172	226	10.10%	7.52%	1.33%	2	0.88%
Tester 2	#2	27146	E10	22	173	319	8.40%	1.90%	0.00%	0	0.00%	10	161	192	11.96%	5.21%	0.52%	0	0.00%
Tester 2	#2	27146	E11	18	237	133	3.12%	7.50%	2.30%	1	0.75%	11	180	92	4.65%	15.22%	5.43%	5	5.43%
Tester 2	#2	27680	E6	19	163	316	10.17%	2.80%	0.30%	1	0.32%	12	114	185	13.47%	12.43%	2.16%	4	2.16%
Tester 2	#2	27680	E7	25	155	317	8.19%	1.30%	0.00%	0	0.00%	13	65	62	7.33%	8.06%	3.23%	2	3.23%
Tester 2	#2	27680	E8	21	227	185	3.88%	0.00%	0.00%	0	0.00%	12	255	72	2.36%	0.00%	0.00%	0	0.00%
Tester 2	#2	28255	E2	10	154	225	14.61%	2.22%	0.00%	0	0.00%	11	105	90	7.81%	16.67%	3.33%	3	3.33%
Tester 2	#2	28291	E2	15	160	308	12.85%	2.27%	0.00%	0	0.00%	11	101	110	9.94%	19.09%	7.27%	6	5.45%
Tester 2	#2	28292	E1	14	143	232	11.59%	0.00%	0.00%	0	0.00%	11	129	111	7.84%	9.91%	2.70%	3	2.70%

Table S9. The zygote editing rate (ZER) under normal and heat treatment conditions

Tester	Trial ID	Vector ID	Event ID	Control (normal condition) F1 diploid No.	ZER_*Wx1*	ZER_*Gl2*	Heat treatment F1 diploid No.	ZER_*Wx1*	ZER_*Gl2*
Tester 1	#1	27145	E2	16	18.75%	0.00%	16	25.00%	18.75%
Tester 1	#1	27145	E5	16	62.50%	6.25%	16	87.50%	0.00%
Tester 1	#1	27146	E4	16	37.50%	0.00%	16	25.00%	0.00%
Tester 1	#1	27146	E6	16	25.00%	0.00%	16	68.75%	31.25%
Tester 1	#1	27146	E7	16	12.50%	0.00%	16	6.25%	0.00%
Tester 1	#1	27680	E2	16	18.75%	6.25%	16	62.50%	6.25%
Tester 1	#1	27680	E3	16	37.50%	12.50%	16	68.75%	50.00%
Tester 1	#2	27145	E3	16	0.00%	0.00%	16	6.25%	31.25%
Tester 1	#2	27145	E4	16	18.80%	0.00%	16	50.00%	0.00%
Tester 1	#2	27146	E9	16	6.30%	0.00%	16	18.75%	12.50%
Tester 1	#2	27146	E10	16	6.30%	0.00%	16	43.75%	18.75%
Tester 1	#2	27146	E11	16	75.00%	6.30%	16	87.50%	0.00%
Tester 1	#2	27680	E6	16	25.00%	6.30%	16	93.75%	6.25%
Tester 1	#2	27680	E7	16	12.50%	0.00%	16	75.00%	43.75%
Tester 1	#2	27680	E8	16	0.00%	0.00%	16	25.00%	50.00%
Tester 2	#1	27145	E2	16	25.00%	6.25%	16	62.50%	50.00%
Tester 2	#1	27146	E7	16	0.00%	0.00%	16	18.75%	0.00%
Tester 2	#1	27680	E3	16	0.00%	6.25%	16	18.75%	18.75%
Tester 2	#2	27145	E3	16	0.00%	0.00%	16	0.00%	18.75%
Tester 2	#2	27145	E4	16	25.00%	6.30%	16	37.50%	0.00%
Tester 2	#2	27145	E6	16	18.80%	6.30%	16	18.75%	18.75%
Tester 2	#2	27146	E9	16	0.00%	0.00%	16	37.50%	25.00%
Tester 2	#2	27146	E10	16	25.00%	0.00%	16	43.75%	0.00%
Tester 2	#2	27146	E11	16	43.80%	6.30%	15	73.33%	6.67%
Tester 2	#2	27680	E6	16	0.00%	0.00%	16	0.00%	6.25%
Tester 2	#2	27680	E7	16	0.00%	0.00%	16	50.00%	50.00%
Tester 2	#2	27680	E8	15	0.00%	0.00%	16	18.75%	62.50%
Tester 2	#2	28255	E2	16	37.50%	12.50%	16	37.50%	43.75%
Tester 2	#2	28291	E2	16	12.50%	6.25%	16	6.25%	31.25%
Tester 2	#2	28292	E1	16	0.00%	0.00%	16	12.50%	0.00%

Table S10. HER of UBA2 fusion and control vector under normal and heat treatment conditions

			Normal condition									Heat treatment								
Vector ID	Event ID	Tester	F1 Ear No.	Kernels /ear	Haploid No.	*Wx1* edited haploid No.	HER_ *Wx1*	*Gl2* edited haploid No.	HER_ *Gl2*	Both target edit haploid No.	HER_ *Wx1 + Gl2*	F1 Ear No.	Kernels /ear	Haploid No.	*Wx1* edited haploid No.	HER_ *Wx1*	*Gl2* edited haploid No.	HER_ *Gl2*	Both target edit haploid No.	HER_ *Wx1 + Gl2*
28825 (UBA2+)	E1	Tester 1	14	155	285	1	0.35%	0	0.00%	0	0.0%	-	-	-	-	-	-	-	-	-
28825 (UBA2+)	E2	Tester 1	14	134	211	12	5.69%	0	0.00%	0	0.0%	-	-	-	-	-	-	-	-	-
28825 (UBA2+)	E3	Tester 1	15	205	278	9	3.24%	0	0.00%	0	0.0%	-	-	-	-	-	-	-	-	-
28825 (UBA2+)	E4	Tester 1	15	161	271	11	4.06%	3	1.10%	1	0.4%	-	-	-	-	-	-	-	-	-
28825 (UBA2+)	E5	Tester 1	14	193	283	12	4.24%	0	0.00%	0	0.0%	-	-	-	-	-	-	-	-	-
28825 (UBA2+)	E6	Tester 1	9	155	152	7	4.61%	0	0.00%	0	0.0%	-	-	-	-	-	-	-	-	-
28825 (UBA2+)	E7	Tester 1	12	98	151	10	6.62%	1	0.70%	1	0.7%	-	-	-	-	-	-	-	-	-
28825 (UBA2+)	E8	Tester 1	14	137	235	12	5.11%	0	0.00%	0	0.0%	-	-	-	-	-	-	-	-	-
28825 (UBA2+)	E1	Tester 2	10	198	255	12	4.71%	0	0.00%	0	0.0%	11	119	242	40	16.53%	14	5.80%	12	5.0%
28825 (UBA2+)	E2	Tester 2	10	191	259	22	8.49%	0	0.00%	0	0.0%	9	82	100	33	33.00%	13	13.00 %	12	12.0%
28825 (UBA2+)	E3	Tester 2	9	239	264	24	9.09%	2	0.80%	1	0.4%	12	131	152	44	28.95%	12	7.90%	11	7.2%
28825 (UBA2+)	E4	Tester 2	10	195	275	22	8.00%	0	0.00%	0	0.0%	11	111	185	48	25.95%	13	7.00%	12	6.5%
28825 (UBA2+)	E5	Tester 2	11	227	245	21	8.57%	1	0.40%	1	0.4%	10	61	73	22	30.14%	7	9.60%	6	8.2%
28825 (UBA2+)	E6	Tester 2	10	172	209	17	8.13%	1	0.50%	1	0.5%	8	82	61	18	29.51%	8	13.10 %	8	13.1%
28825 (UBA2+)	E7	Tester 2	10	165	268	12	4.48%	2	0.70%	1	0.4%	8	89	89	21	23.60%	9	10.10 %	7	7.9%
28825 (UBA2+)	E8	Tester 2	10	158	170	22	12.94%	2	1.20%	2	1.2%	8	65	98	26	26.53%	10	10.20 %	9	9.2%
27680 (CK)	E1	Tester 1	20	207	223	0	0.00%	1	0.40%	0	0.0%	-	-	-	-	-	-	-	-	-
27680 (CK)	E2	Tester 1	20	147	264	0	0.00%	0	0.00%	0	0.0%	-	-	-	-	-	-	-	-	-
27680 (CK)	E3	Tester 1	21	157	318	0	0.00%	0	0.00%	0	0.0%	-	-	-	-	-	-	-	-	-
27680 (CK)	E4	Tester 1	16	232	315	2	0.63%	0	0.00%	0	0.0%	-	-	-	-	-	-	-	-	-
27680 (CK)	E5	Tester 1	13	228	315	0	0.00%	0	0.00%	0	0.0%	-	-	-	-	-	-	-	-	-
27680 (CK)	E6	Tester 1	25	149	316	6	1.90%	0	0.00%	0	0.0%	-	-	-	-	-	-	-	-	-
27680 (CK)	E7	Tester 1	21	124	232	1	0.43%	0	0.00%	0	0.0%	-	-	-	-	-	-	-	-	-
27680 (CK)	E8	Tester 1	23	235	162	0	0.00%	0	0.00%	0	0.0%	-	-	-	-	-	-	-	-	-
27680 (CK)	E9	Tester 1	20	173	317	0	0.00%	0	0.00%	0	0.0%	-	-	-	-	-	-	-	-	-
27680 (CK)	E1	Tester 2	23	129	145	2	1.38%	0	0.00%	0	0.0%	-	-	-	-	-	-	-	-	-
27680 (CK)	E2	Tester 2	20	163	315	6	1.90%	1	0.30%	1	0.3%	-	-	-	-	-	-	-	-	-
27680 (CK)	E3	Tester 2	16	215	319	5	1.57%	2	0.60%	0	0.0%	7	112	76	6	7.89%	4	5.30%	3	3.9%
27680 (CK)	E4	Tester 2	24	171	309	5	1.62%	0	0.00%	0	0.0%	-	-	-	-	-	-	-	-	-
27680 (CK)	E5	Tester 2	20	160	316	6	1.90%	2	0.60%	0	0.0%	-	-	-	-	-	-	-	-	-
27680 (CK)	E6	Tester 2	19	163	316	9	2.85%	1	0.30%	1	0.3%	12	114	185	23	12.43%	4	2.20%	4	2.2%
27680 (CK)	E7	Tester 2	25	155	317	4	1.26%	0	0.00%	0	0.0%	13	67	62	5	8.06%	2	3.20%	2	3.2%
27680 (CK)	E8	Tester 2	21	227	185	0	0.00%	0	0.00%	0	0.0%	12	254	72	0	0.00%	-	-	-	-
27680 (CK)	E9	Tester 2	17	183	318	3	0.94%	0	0.00%	0	0.0%	-	-	-	-	-	-	-	-	-

Table S11. ZER of UBA2 fusion and control vector under normal and heat treatment conditions

Vector ID	Event ID	Tester	Normal condition (CK)		Heat treatment	
			F1 diploid No.	ZER_*Wx1*	F1 diploid No.	ZER_*Wx1*
28825 (UBA2+)	E1	Tester 1	48	18.75%	48	52.08%
28825 (UBA2+)	E2	Tester 1	48	62.50%	48	18.75%
28825 (UBA2+)	E3	Tester 1	48	50.00%	48	79.17%
28825 (UBA2+)	E4	Tester 1	48	64.58%	48	25.00%
28825 (UBA2+)	E5	Tester 1	48	81.25%	48	60.42%
28825 (UBA2+)	E6	Tester 1	48	50.00%	48	68.75%
28825 (UBA2+)	E7	Tester 1	48	81.25%	48	75.00%
28825 (UBA2+)	E8	Tester 1	48	60.42%	48	75.00%
28825 (UBA2+)	E1	Tester 2	48	8.33%	-	-
28825 (UBA2+)	E2	Tester 2	48	60.42%	-	-
28825 (UBA2+)	E3	Tester 2	48	50.00%	-	-
28825 (UBA2+)	E4	Tester 2	48	31.25%	-	-
28825 (UBA2+)	E5	Tester 2	48	39.58%	-	-
28825 (UBA2+)	E6	Tester 2	48	41.67%	-	-
28825 (UBA2+)	E7	Tester 2	48	54.17%	-	-
28825 (UBA2+)	E8	Tester 2	48	54.17%	-	-
27680 (CK)	E1	Tester 1	16	12.50%	-	-
27680 (CK)	E2	Tester 1	16	18.75%	-	-
27680 (CK)	E3	Tester 1	16	37.50%	16	18.75%
27680 (CK)	E4	Tester 1	16	0.00%	-	-
27680 (CK)	E5	Tester 1	16	6.25%	-	-
27680 (CK)	E6	Tester 1	16	25.00%	16	0.00%
27680 (CK)	E7	Tester 1	16	12.50%	16	50.00%
27680 (CK)	E8	Tester 1	16	0.00%	16	18.75%
27680 (CK)	E9	Tester 1	16	25.00%	-	-
27680 (CK)	E1	Tester 2	16	0.00%	-	-
27680 (CK)	E2	Tester 2	16	6.25%	-	-
27680 (CK)	E3	Tester 2	16	0.00%	-	-
27680 (CK)	E4	Tester 2	16	18.75%	-	-
27680 (CK)	E5	Tester 2	16	0.00%	-	-
27680 (CK)	E6	Tester 2	16	0.00%	-	-
27680 (CK)	E7	Tester 2	16	0.00%	-	-
27680 (CK)	E8	Tester 2	15	0.00%	-	-
27680 (CK)	E9	Tester 2	16	0.00%	-	-

Table S12. The ZER assay of reciprocal crosses

Vector	Outcross (Female / male)	As Male F1#	As Male ZER_*Wx1*	Outcross (Female / male)	As Female F1#	As Female ZER_*Wx1*
27145 (*prZmRZDP*)	Tester 1 / E4	16	19%	E436-1 / Tester 1	48	90%
27145 (*prZmRZDP*)	Tester 2 / E4	16	25%	E436-1 / Tester 2	48	52%
27145 (*prZmRZDP*)	Tester 1 / E5	16	63%	E515-1 / Tester 1	48	79%
27145 (*prZmRZDP*)	Tester 2 / E5	16	31%	E515-1 / Tester 2	48	52%
27146 (*prZmVSP*)	Tester 1 / E10	16	6%	E103-1 / Tester 1	48	13%
27146 (*prZmVSP*)	Tester 2 / E10	16	25%	E103-1 / Tester 2	42	10%
27146 (*prZmVSP*)	Tester 1 / E11	16	75%	E1110-1 / Tester 1	48	90%
27146 (*prZmVSP*)	Tester 2 / E11	16	44%	E1110-1 / Tester 2	48	75%
27680 (*prSoUbi4*)	Tester 1 / E3	16	38%	E310-1 / Tester 1	48	88%
27680 (*prSoUbi4*)	Tester 2 / E3	16	0%	E310-1 / Tester 2	48	63%
27680 (*prSoUbi4*)	Tester 1 / E6	16	25%	E64-5-1 / Tester 1	48	67%
27680 (*prSoUbi4*)	Tester 2 / E6	16	0%	E64-5-1 / Tester 2	48	0%
27680 (*prSoUbi4*)	Tester 1 / E7	16	13%	E75-1 / Tester 1	48	71%
27680 (*prSoUbi4*)	Tester 2 / E7	16	0%	E75-1 / Tester 2	48	58%

Table S13. Quantification of Cas12aV protein in leaf samples from UBA2 fusion and control (CK) vectors

Vector ID	Event ID	LbCAS12aV (fmol/µg)	STDEV	CV (%)
27680 (CK)	E1	361.70	9.20	2.54
27680 (CK)	E2	331.27	6.62	2.00
27680 (CK)	E3	262.16	5.12	1.95
27680 (CK)	E4	276.08	5.05	1.83
27680 (CK)	E5	304.59	8.84	2.90
27680 (CK)	E6	246.55	2.13	0.86
27680 (CK)	E7	257.65	8.97	3.48
27680 (CK)	E8	208.34	8.30	3.99
27680 (CK)	E9	278.16	21.60	7.77
28825 (UBA2+)	E4	369.26	31.34	8.49
28825 (UBA2+)	E5	411.40	130.09	31.62
28825 (UBA2+)	E6	285.75	24.45	8.56
28825 (UBA2+)	E7	371.28	0.71	0.19
28825 (UBA2+)	E8	330.66	6.39	1.93

Table S14. HER for different events with different copy number and insertion sites in the genome

Vector ID	Event ID	Copy Number	Location in Maize Reference genome	Inserted in gene	Inserted in potential promoter	Inserted in potential terminator	HER _Wx1
27145 (*prZmRZDP*)	E1	1	Chr1:190,113,187-190,088,451	No	No	Yes	0.63%
27145 (*prZmRZDP*)	E2	1	Chr5:207,745,203-207,745,312	No	No	No	0.64%
27145 (*prZmRZDP*)	E5	1	Chr1:236,168,339-236,168,347	Yes	—	—	3.47%
27145 (*prZmRZDP*)	E6	1	Chr6:158,094,768-158,096,763	No	No	Yes	0.78%
27146 (*prZmVSP*)	E1	1	Chr5:185,175,005-185,189,111	No	No	No	1.27%
27146 (*prZmVSP*)	E5	1	Chr2:146,124,153-146,124,048	No	No	No	0.61%
27146 (*prZmVSP*)	E7	1	Chr8:19,866,063-19,866,009	Yes	—	—	0.60%
27146 (*prZmVSP*)	E8	1	Left: Chr8:169,084,796<br>Right: Chr1:51,519,279.	No	No	No	0.62%
27146 (*prZmVSP*)	E9	1	Chr5:1,335,161-1,335,120	Yes	No	No	0.95%
27146 (*prZmVSP*)	E10	1	Chr4:176,924,943-176,926,008	Yes	—	—	1.88%
27146 (*prZmVSP*)	E11	4	Chr6:166,351,473	Yes	—	—	7.52%

Table S15. Primers and probes of Taqman assays used for copy number of events, LbCas12aV expression and absence of CRISPR reagents introgression in haploid samples

Gene	Assay ID	Primer/Probe	Sequence (5'-3')	Amplicon Size
PMI	*1393*	Forward	GCTGTAAGAGCTTACTGAAAAAATTAACA	72 bp
		Reverse	CGATCTGCAGGTCGACGG	
		Probe	FAM-TCTCTTGCTAAGCTGGGAGCTCGATCC-BHQ-1	
LbCas12aV	*3633*	Forward	CAACCTCTACAACCAGAAGACCAA	74 bp
		Reverse	TGTCGGAGAGCACCTGCTT	
		Probe	FAM-AAGCTGCCGAAGTTCAAGCCCCTG-BHQ-1	
Waxy1	*3644*	Forward	GCCTGGTCGCTGGTTTCAG	82 bp
		Reverse	GTTGTCCCTGTAGTCCGTTCCA	
		Probe	FAM-AGACCGAGGAGAAGAT-MGB	
Glossy2	*3658*	Forward	CACGGTACATGCCCTAACTACG	129 bp
		Reverse	TGTCGCCGATGAGGTGC	
		Probe	FAM-CCGCATTTGAAGTTTGT-MGB	
ADH1	*1091*	Forward	AGGTGTGGATCGGGCTGTT	74 bp
		Reverse	CATCGTGGACGCATTCGA	
		Probe	TET-ACTGGCAGCATCCAAGCCATGGTCT-BHQ-1	
EF1a	*1526*	Forward	GCGCCGTCACCGTATCC	68 bp
		Reverse	GCTCGTCGGGCGTCAGTA	
		Probe	TET-ATCAGAGGCGAGCAGAAACCACACCAC-BHQ-1	

Table S16. Primers used for amplifying target genes

Gene	Primer/Probe	Sequence (5'-3')	Amplicon Size
Wx1	Forward	AGATGGGAGACGGGTACGAGACGG	410 bp
	Reverse	GTATGGGTTGTTGTTGAGGCTCAGG	
Gl2	Forward	GCTGACGTGGAAGGAGTAGCA	421 bp
	Reverse	ACACCGTGTCTTCGTCAAAATACA	
